# Supplementary material for: Differences in persistence between dogs and wolves in an unsolvable task in the absence of humans
Source: PeerJ. 2018 Nov 27;6:e5944. doi: 10.7717/peerj.5944 (PMC6266929; doi:10.7717/peerj.5944)
Supplement: Supplemental Information 2 [file peerj-06-5944-s002.docx]

Supplementary Material

Differences in persistence between dogs and wolves in an unsolvable task in the absence of humans.

Akshay Rao^1,2* +^, Lara Bernasconi^2,3 +^, Martina Lazzaroni^1,2^, Sarah Marshall-Pescini^1,2^, Friederike Range^1,2^

**+** These authors contributed equally to this research

*** Correspondence:** Akshay Rao: [akshay.rao@vetmeduni.ac.at](mailto:akshay.rao@vetmeduni.ac.at)

Due to the length of this file, the contents have been organized with collapsible sections.

To expand a section, click the triangular button to the left of the heading text.

# Supplementary Results

#### Descriptive statistics of each correlate in dogs and wolves.

| **Variable** | **Statistic** | **Ball** | | **Pipe** | |
| --- | --- | --- | --- | --- | --- |
|  |  | **Dogs** | **Wolves** | **Dogs** | **Wolves** |
| Contact Latency (Seconds) | Min | 0.6 | 0.4 | 0.6 | 0.2 |
|  | Max | 2.0 | 2.0 | 2.4 | 3.2 |
|  | Mean | 1.0 | 1.2 | 1.1 | 1.3 |
|  | Median | 0.9 | 1.2 | 1.0 | 1.0 |
|  | Std. Dev | 0.4 | 0.7 | 0.5 | 0.9 |
| Persistence (Seconds) | Min | 0.0 | 14.6 | 0.0 | 0.2 |
|  | Max | 282.8 | 940.8 | 821.4 | 950.6 |
|  | Mean | 29.4 | 319.0 | 97.3 | 239.9 |
|  | Median | 4.1 | 169.2 | 2.4 | 45.7 |
|  | Std. Dev | 73.3 | 299.2 | 244.2 | 356.8 |
| Motor Diversity | Min | 0 | 6 | 0 | 1 |
|  | Max | 13 | 17 | 14 | 14 |
|  | Mean | 3 | 10 | 3 | 7 |
|  | Median | 3 | 9 | 2 | 6 |
|  | Std. Dev | 3 | 3 | 4 | 5 |

#### Between-species comparison of intra-individual consistency in problem-solving behaviours (plot)

#### Results for analyses for persistence including outliers

The results from the model built using the Gamma distribution (AIC = 500.6998) differed from the results from models built using the Box-Cox T Original (AIC = 505.0368), Weibull (AIC = 509.0868) and Log-Normal (AIC = 826.7205) distributions (results from these three distributions were the same). This implies that these models may not be robust and that the following results should be interpreted with caution.

When modelled using the Box-Cox T Original distribution, wolves were more persistent than dogs (GAMLSS: t = 2.21, P = 0.032) in their manipulation of both objects (i.e. the interaction between species and object was not significant, GAMLSS: t = -1.65, P = 0.10). Subjects’ persistence did not differ between objects (GAMLSS: t = -0.36, P = 0.72) and was not affected by age (GAMLSS: t = 1.07, P = 0.29).

When modelled using the Gamma distribution, dogs and wolves did not differ significantly in their persistence in manipulating either object (GAMLSS: t = 1.34, P = 0.19) (i.e., the interaction between species and object was not significant, GAMLSS: t = -1.81, P = 0.08). Subjects’ persistence did not differ between objects (GAMLSS: t = 1.46, P – 0.15) and was not affected by age (GAMLSS: t = 0.92, P = 0.36).

# Complete GAMLSS Model Information

## Persistence Models

#### Response Variable Distribution Checks

###### Persistence.Distribution$fit

| Distribution | AIC | Errors in Plot? |
| --- | --- | --- |
| BCPEo | 444.935388 | Yes |
| GG | 455.096973 | Yes |
| GB2 | 457.614243 | Yes |
| GIG | 457.950489 | Yes |
| IGAMMA | 547.4639 | No |
| GA | 458.178068 | No |
| BCCGo | 463.276357 | No |
| BCTo | 465.276357 | No |
| WEI2 | 469.313586 | No |
| WEI | 469.313586 | No |
| WEI3 | 469.313586 | No |
| LOGNO2 | 494.746702 | No |
| LOGNO | 494.746702 | No |

##### Plots


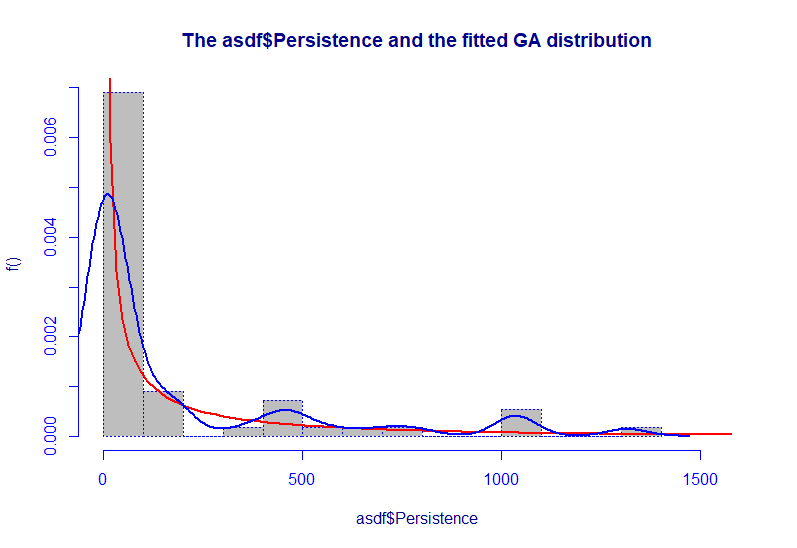

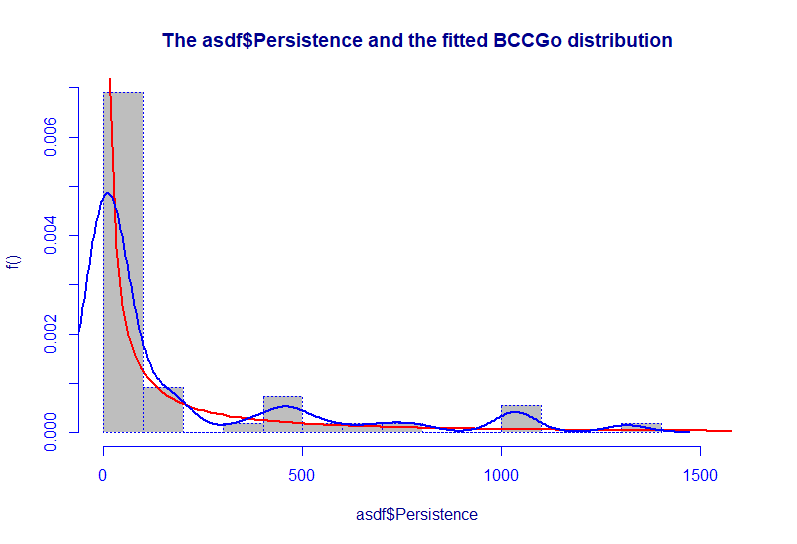


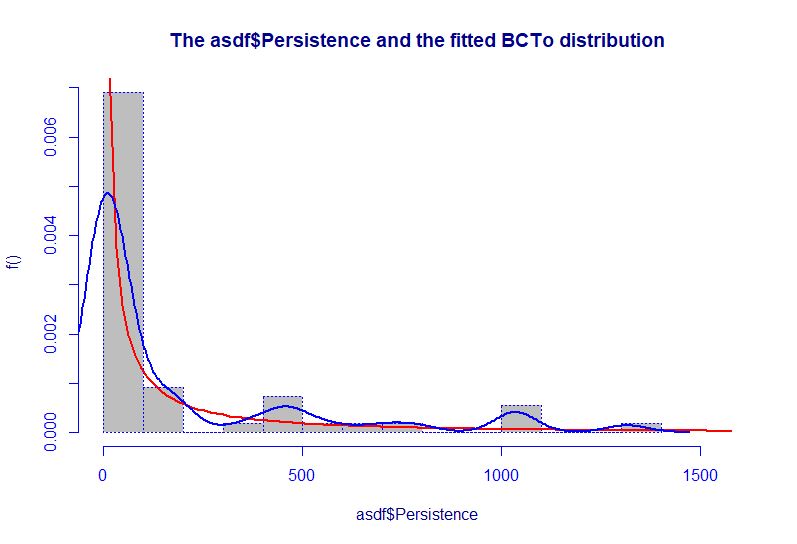

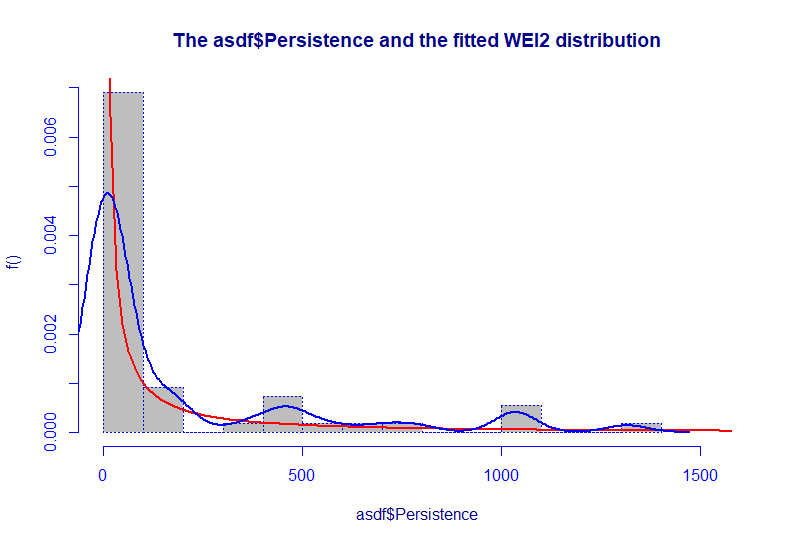


#### Model Distribution Selection

Persistence.DISTRIBUTION <- gamlss(Persistence ~ Species*Object + Age,

random = ~1|Individual, family = "DISTRIBUTION",
data = asdf)

| Model | df | AIC | Residuals outside CI |
| --- | --- | --- | --- |
| Persistence.GA | 6 | 471.144609 | 3 |
| Persistence.BCTo | 8 | 472.382229 | 4 |
| Persistence.WEI | 6 | 478.48115 | 14 |
| Persistence.WEI3 | 6 | 478.481189 | 14 |
| Persistence.WEI2 | 6 | 478.481915 | 13 |
| Persistence.LOGNO2 | 6 | 780.468216 | 23 |
| Persistence.LOGNO | 6 | 780.468216 | 23 |
| Persistence.BCCGo | 7 | 9375.03115 | - |

###### plot(Persistence.GA) & wp(Persistence.GA)


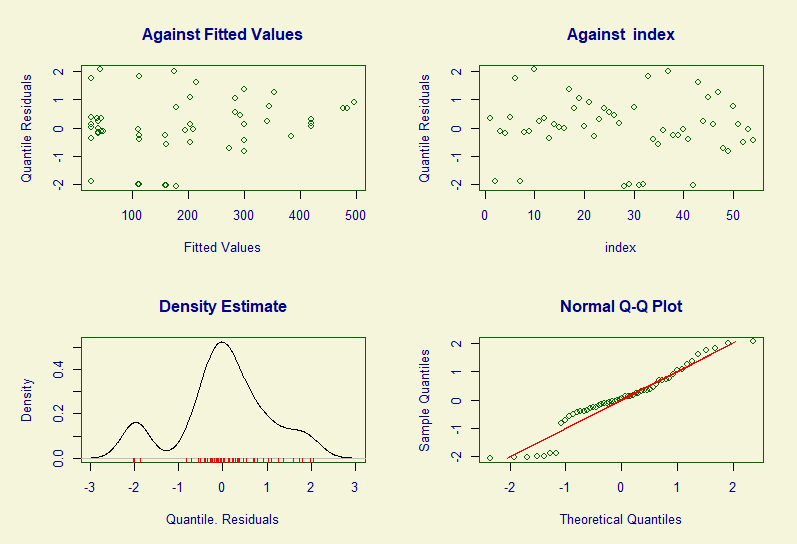

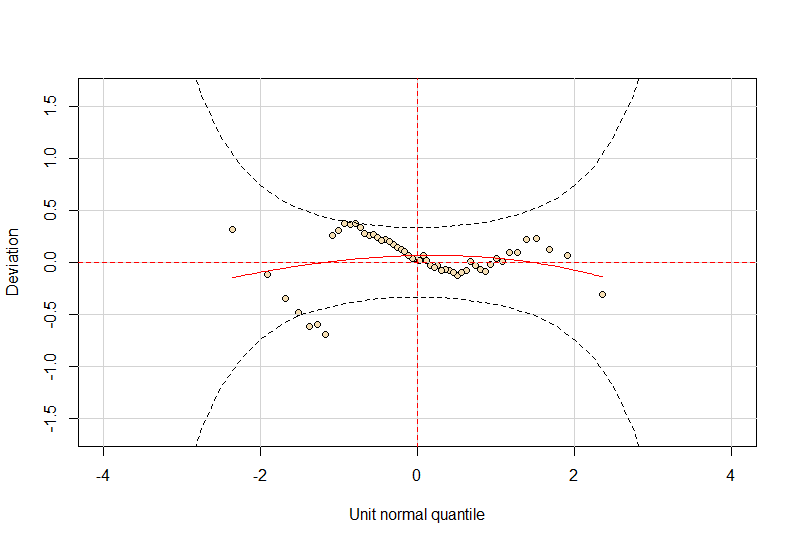


###### plot(Persistence.BCTo) & wp(Persistence.BCTo)


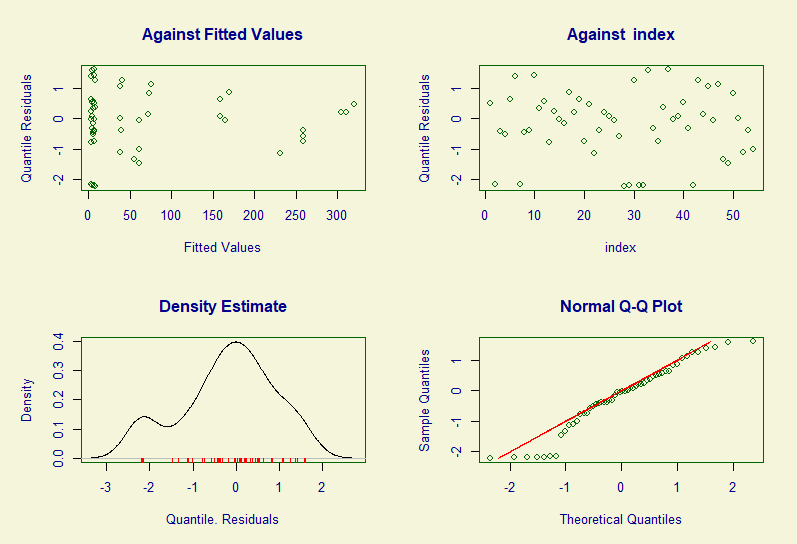

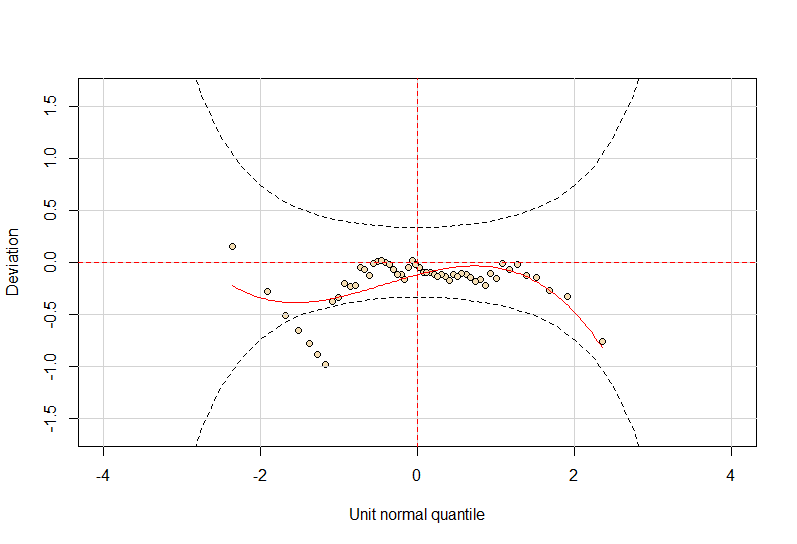


###### plot(Persistence.WEI) & wp(Persistence.WEI)


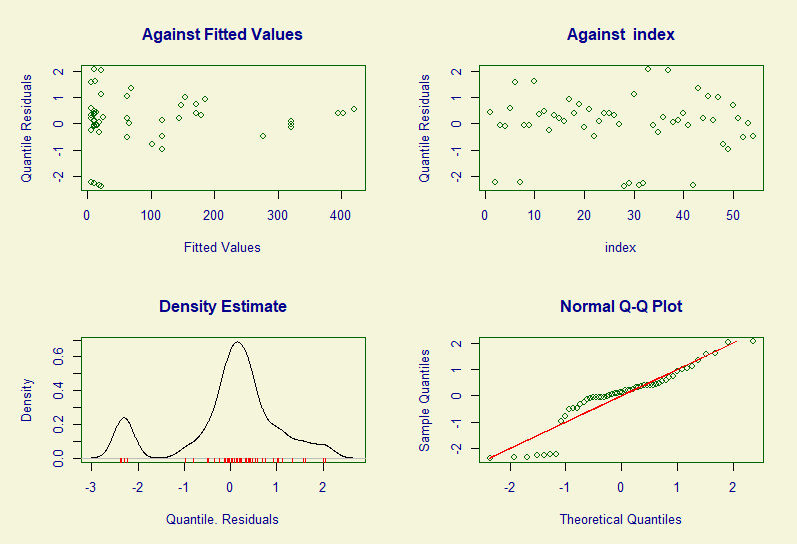

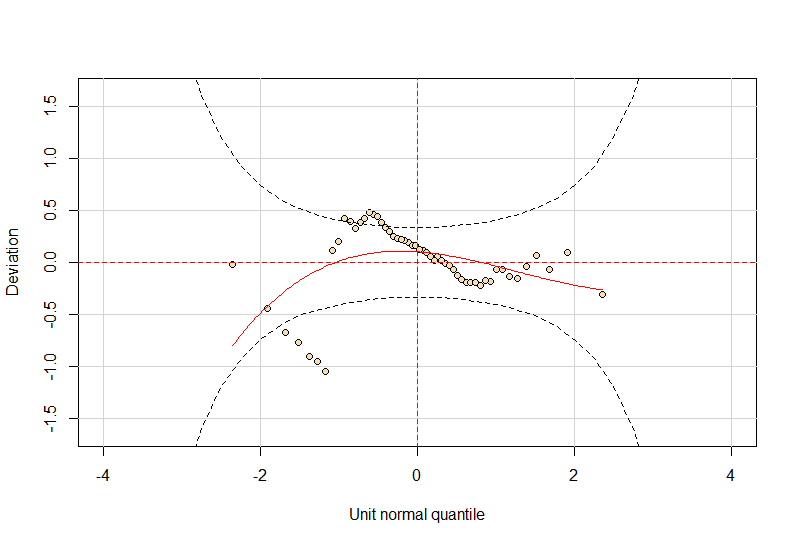


###### plot(Persistence.WEI3) & wp(Persistence.WEI3)


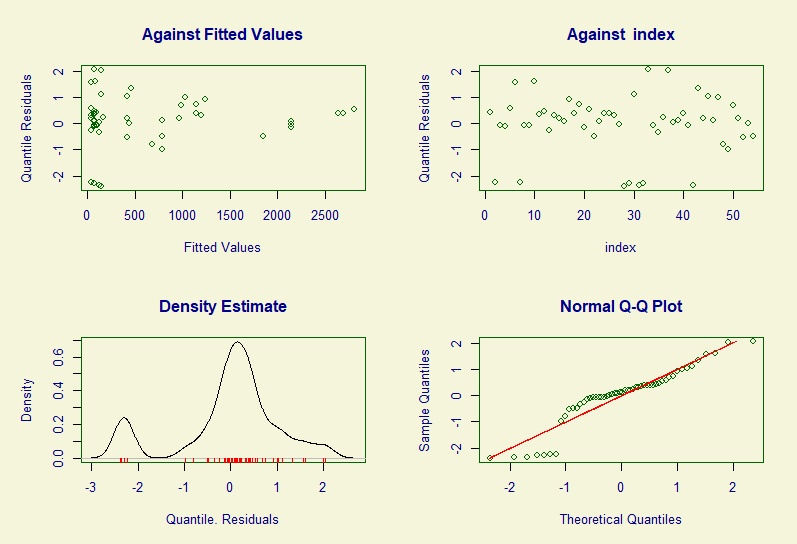

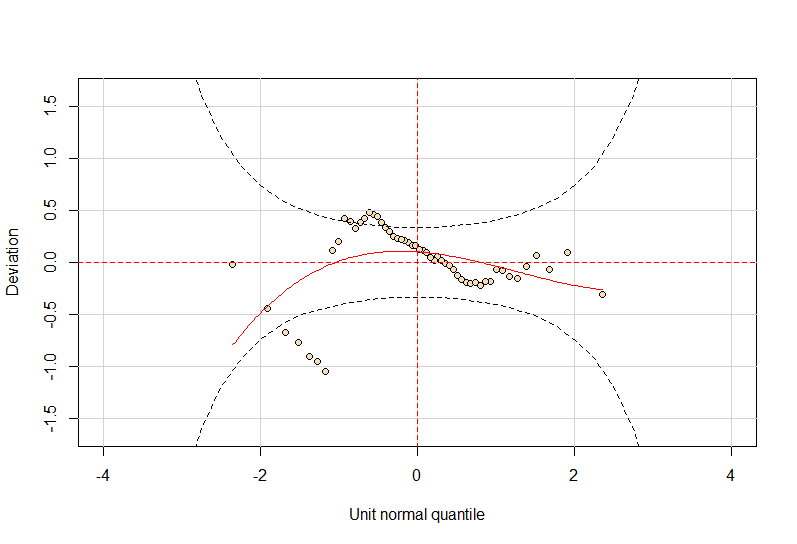


###### plot(Persistence.WEI2) & wp(Persistence.WEI2)


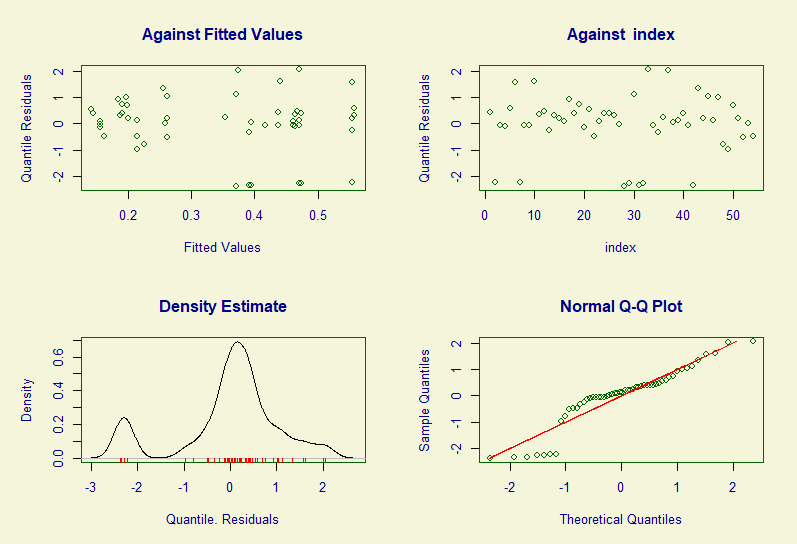

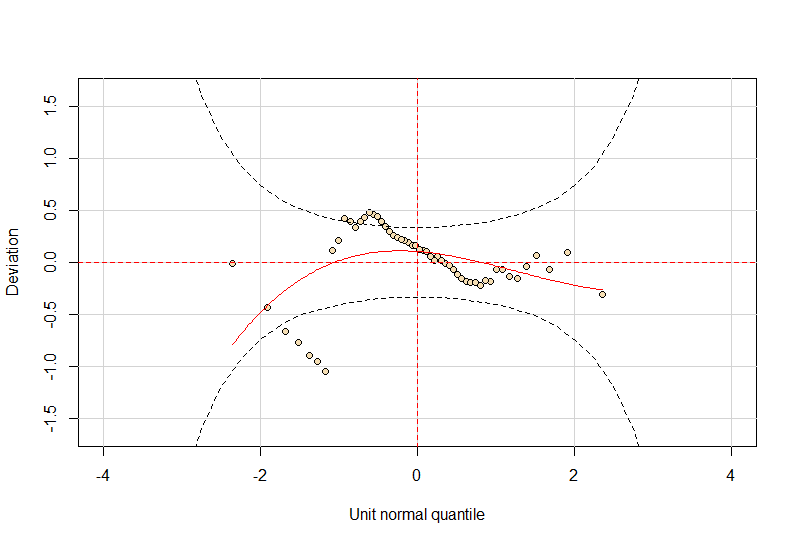


###### plot(Persistence.LOGNO2) & wp(Persistence.LOGNO2)


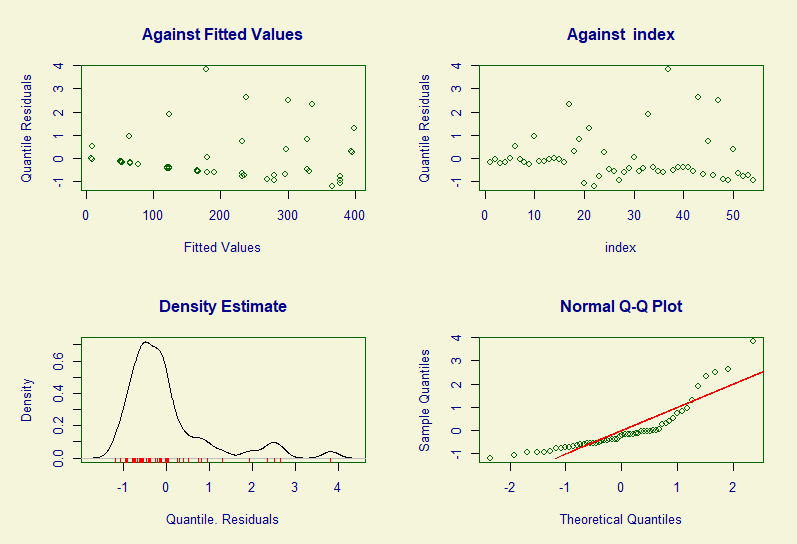

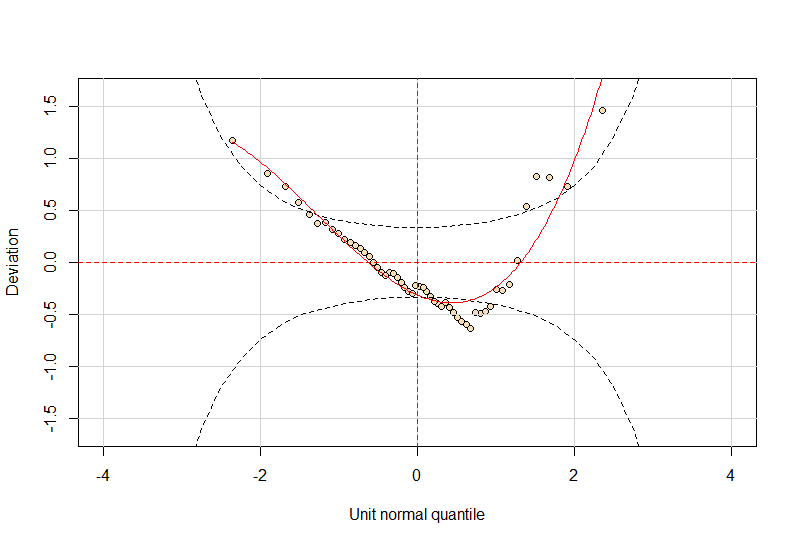


###### plot(Persistence.LOGNO) & wp(Persistence.LOGNO)


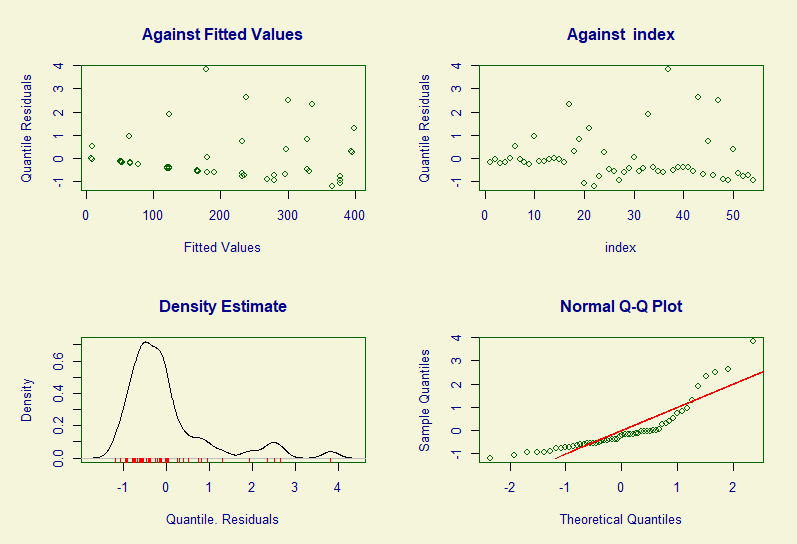

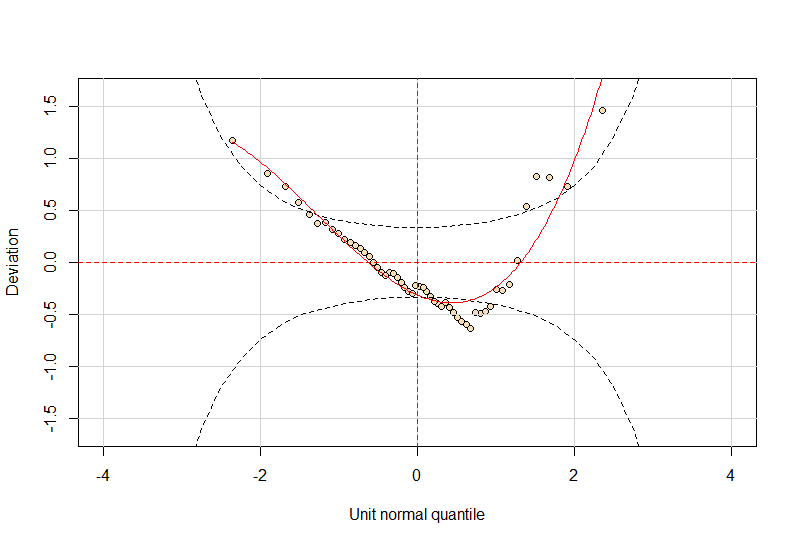


#### Model Reduction & Validation

##### Gamma Models

###### summary(Persistence.GA)

******************************************************************

Family: c("GA", "Gamma")

Call: gamlss(formula = Persistence ~ Species * Object + Age,

family = "GA", data = na.omit(asdf), control = con, random = ~1 | Individual)

Fitting method: RS()

------------------------------------------------------------------

Mu link function: log

Mu Coefficients:

Estimate Std. Error t value Pr(>|t|)

(Intercept) 3.0281 0.8996 3.366 0.00151 **

SpeciesWolf 2.0694 0.8809 2.349 0.02297 *

ObjectPipe 1.3928 0.7705 1.808 0.07692 .

Age 0.1292 0.1653 0.782 0.43815

SpeciesWolf:ObjectPipe -1.7297 1.1809 -1.465 0.14950

---

Signif. codes: 0 ‘***’ 0.001 ‘**’ 0.01 ‘*’ 0.05 ‘.’ 0.1 ‘ ’ 1

------------------------------------------------------------------

Sigma link function: log

Sigma Coefficients:

Estimate Std. Error t value Pr(>|t|)

(Intercept) 0.76252 0.07419 10.28 1.02e-13 ***

------------------------------------------------------------------

No. of observations in the fit: 54

Degrees of Freedom for the fit: 6

Residual Deg. of Freedom: 48

at cycle: 2

Global Deviance: 459.1446

AIC: 471.1446

SBC: 483.0785

******************************************************************

###### dropterm(Persistence.GA, test = "Chisq")

Single term deletions for mu

Model: Persistence ~ Species * Object + Age

Df AIC LRT Pr(Chi)

<none> 471.14

Age 1 469.75 0.60761 0.4357

Species:Object 1 471.22 2.07727 0.1495

###### summary(Persistence.GA.2)

******************************************************************

Family: c("GA", "Gamma")

Call: gamlss(formula = Persistence ~ Species + Object + Age,

family = "GA", data = na.omit(asdf), control = con, random = ~1 | Individual)

Fitting method: RS()

------------------------------------------------------------------

Mu link function: log

Mu Coefficients:

Estimate Std. Error t value Pr(>|t|)

(Intercept) 3.4547 0.9266 3.728 0.0005 ***

SpeciesWolf 1.2552 0.6993 1.795 0.0788 .

ObjectPipe 0.6699 0.6352 1.055 0.2968

Age 0.1266 0.1674 0.756 0.4533

---

Signif. codes: 0 ‘***’ 0.001 ‘**’ 0.01 ‘*’ 0.05 ‘.’ 0.1 ‘ ’ 1

------------------------------------------------------------------

Sigma link function: log

Sigma Coefficients:

Estimate Std. Error t value Pr(>|t|)

(Intercept) 0.77393 0.07407 10.45 4.6e-14 ***

------------------------------------------------------------------

No. of observations in the fit: 54

Degrees of Freedom for the fit: 5

Residual Deg. of Freedom: 49

at cycle: 2

Global Deviance: 461.2219

AIC: 471.2219

SBC: 481.1668

******************************************************************

##### Box-Cox T Original Models

###### summary(Persistence.BCTo)

******************************************************************

Family: c("BCTo", "Box-Cox-t-orig.")

Call: gamlss(formula = Persistence ~ Species * Object + Age,

family = "BCTo", data = na.omit(asdf), control = con, random = ~1 | Individual)

Fitting method: RS()

------------------------------------------------------------------

Mu link function: log

Mu Coefficients:

Estimate Std. Error t value Pr(>|t|)

(Intercept) 0.9022 0.7954 1.134 0.263

SpeciesWolf 3.4660 0.7307 4.744 2.07e-05 ***

ObjectPipe 0.1557 0.7024 0.222 0.826

Age 0.1630 0.1487 1.096 0.279

SpeciesWolf:ObjectPipe -1.5952 1.0694 -1.492 0.143

---

Signif. codes: 0 ‘***’ 0.001 ‘**’ 0.01 ‘*’ 0.05 ‘.’ 0.1 ‘ ’ 1

------------------------------------------------------------------

Sigma link function: log

Sigma Coefficients:

Estimate Std. Error t value Pr(>|t|)

(Intercept) 0.3788 0.3357 1.128 0.265

------------------------------------------------------------------

Nu link function: identity

Nu Coefficients:

Estimate Std. Error t value Pr(>|t|)

(Intercept) 0.10731 0.07888 1.36 0.18

------------------------------------------------------------------

Tau link function: log

Tau Coefficients:

Estimate Std. Error t value Pr(>|t|)

(Intercept) 0.1502 0.4464 0.337 0.738

------------------------------------------------------------------

No. of observations in the fit: 54

Degrees of Freedom for the fit: 8

Residual Deg. of Freedom: 46

at cycle: 20

Global Deviance: 456.3822

AIC: 472.3822

SBC: 488.2941

******************************************************************

###### dropterm(Persistence.BCTo, test = "Chisq")

Single term deletions for mu

Model: Persistence ~ Species * Object + Age

Df AIC LRT Pr(Chi)

<none> 472.38

Age 1 471.51 1.1258 0.2887

Species:Object 1 472.26 1.8732 0.1711

###### summary(Persistence.BCTo2)

******************************************************************

Family: c("BCTo", "Box-Cox-t-orig.")

Call: gamlss(formula = Persistence ~ Species + Object + Age,

family = "BCTo", data = na.omit(asdf), control = con, random = ~1 | Individual)

Fitting method: RS()

------------------------------------------------------------------

Mu link function: log

Mu Coefficients:

Estimate Std. Error t value Pr(>|t|)

(Intercept) 1.1990 0.9015 1.330 0.189943

SpeciesWolf 2.9267 0.7718 3.792 0.000426 ***

ObjectPipe -0.4757 0.6160 -0.772 0.443868

Age 0.1361 0.1760 0.774 0.443078

---

Signif. codes: 0 ‘***’ 0.001 ‘**’ 0.01 ‘*’ 0.05 ‘.’ 0.1 ‘ ’ 1

------------------------------------------------------------------

Sigma link function: log

Sigma Coefficients:

Estimate Std. Error t value Pr(>|t|)

(Intercept) 0.5673 0.3778 1.502 0.14

------------------------------------------------------------------

Nu link function: identity

Nu Coefficients:

Estimate Std. Error t value Pr(>|t|)

(Intercept) 0.13653 0.08641 1.58 0.121

------------------------------------------------------------------

Tau link function: log

Tau Coefficients:

Estimate Std. Error t value Pr(>|t|)

(Intercept) 0.3865 0.5790 0.667 0.508

------------------------------------------------------------------

No. of observations in the fit: 54

Degrees of Freedom for the fit: 7

Residual Deg. of Freedom: 47

at cycle: 21

Global Deviance: 458.2554

AIC: 472.2554

SBC: 486.1783

******************************************************************

##### Weibull Models

###### summary(Persistence.WEI)

******************************************************************

Family: c("WEI", "Weibull")

Call: gamlss(formula = Persistence ~ Species * Object + Age,

family = "WEI", data = na.omit(asdf), control = con, random = ~1 | Individual)

Fitting method: RS()

------------------------------------------------------------------

Mu link function: log

Mu Coefficients:

Estimate Std. Error t value Pr(>|t|)

(Intercept) 1.3296 1.2897 1.031 0.3078

SpeciesWolf 2.9151 1.3044 2.235 0.0301 *

ObjectPipe 0.5124 1.1156 0.459 0.6480

Age 0.2089 0.2417 0.864 0.3917

SpeciesWolf:ObjectPipe -1.5139 1.6988 -0.891 0.3773

---

Signif. codes: 0 ‘***’ 0.001 ‘**’ 0.01 ‘*’ 0.05 ‘.’ 0.1 ‘ ’ 1

------------------------------------------------------------------

Sigma link function: log

Sigma Coefficients:

Estimate Std. Error t value Pr(>|t|)

(Intercept) -1.1253 0.1144 -9.836 4.33e-13 ***

------------------------------------------------------------------

No. of observations in the fit: 54

Degrees of Freedom for the fit: 6

Residual Deg. of Freedom: 48

at cycle: 4

Global Deviance: 466.4812

AIC: 478.4812

SBC: 490.4151

******************************************************************

###### dropterm(Persistence.WEI, test = "Chisq")

Single term deletions for mu

Model: Persistence ~ Species * Object + Age

Df AIC LRT Pr(Chi)

<none> 478.48

Age 1 477.22 0.74338 0.3886

Species:Object 1 477.26 0.78022 0.3771

###### summary(Persistence.WEI2)

******************************************************************

Family: c("WEI", "Weibull")

Call: gamlss(formula = Persistence ~ Species + Object + Age,

family = "WEI", data = na.omit(asdf), control = con, random = ~1 | Individual)

Fitting method: RS()

------------------------------------------------------------------

Mu link function: log

Mu Coefficients:

Estimate Std. Error t value Pr(>|t|)

(Intercept) 1.6360 1.2767 1.281 0.2061

SpeciesWolf 2.1743 1.0010 2.172 0.0347 *

ObjectPipe -0.1444 0.8599 -0.168 0.8673

Age 0.2104 0.2451 0.858 0.3948

---

Signif. codes: 0 ‘***’ 0.001 ‘**’ 0.01 ‘*’ 0.05 ‘.’ 0.1 ‘ ’ 1

------------------------------------------------------------------

Sigma link function: log

Sigma Coefficients:

Estimate Std. Error t value Pr(>|t|)

(Intercept) -1.1399 0.1135 -10.04 1.77e-13 ***

------------------------------------------------------------------

No. of observations in the fit: 54

Degrees of Freedom for the fit: 5

Residual Deg. of Freedom: 49

at cycle: 4

Global Deviance: 467.2614

AIC: 477.2614

SBC: 487.2063

******************************************************************

##### Log-Normal Models

###### summary(Persistence.LOGNO)

******************************************************************

Family: c("NO", "Normal")

Call: gamlss(formula = Persistence ~ Species * Object + Age,

data = na.omit(asdf), control = con, random = ~1 | Individual, Family = "LOGNO")

Fitting method: RS()

------------------------------------------------------------------

Mu link function: identity

Mu Coefficients:

Estimate Std. Error t value Pr(>|t|)

(Intercept) -29.64 125.99 -0.235 0.8150

SpeciesWolf 289.25 130.70 2.213 0.0317 *

ObjectPipe 113.49 107.04 1.060 0.2943

Age 16.22 24.68 0.657 0.5143

SpeciesWolf:ObjectPipe -211.17 164.07 -1.287 0.2042

---

Signif. codes: 0 ‘***’ 0.001 ‘**’ 0.01 ‘*’ 0.05 ‘.’ 0.1 ‘ ’ 1

------------------------------------------------------------------

Sigma link function: log

Sigma Coefficients:

Estimate Std. Error t value Pr(>|t|)

(Intercept) 5.69651 0.09622 59.2 <2e-16 ***

------------------------------------------------------------------

No. of observations in the fit: 54

Degrees of Freedom for the fit: 6

Residual Deg. of Freedom: 48

at cycle: 2

Global Deviance: 768.4682

AIC: 780.4682

SBC: 792.4021

******************************************************************

###### dropterm(Persistence.LOGNO, test = "Chisq")

Single term deletions for mu

Model: Persistence ~ Species * Object + Age

Df AIC LRT Pr(Chi)

<none> 780.47

Age 1 778.90 0.43004 0.5120

Species:Object 1 780.10 1.63146 0.2015

###### summary(Persistence.LOGNO2)

******************************************************************

Family: c("LOGNO", "Log Normal")

Call: gamlss(formula = Persistence ~ Species + Object + Age,

family = "LOGNO", data = na.omit(asdf), control = con, random = ~1 | Individual)

Fitting method: RS()

------------------------------------------------------------------

Mu link function: identity

Mu Coefficients:

Estimate Std. Error t value Pr(>|t|)

(Intercept) -1.3138 1.9207 -0.684 0.49717

SpeciesWolf 4.2744 1.5764 2.712 0.00921 **

ObjectPipe -1.9848 1.2785 -1.552 0.12698

Age 0.3988 0.3887 1.026 0.30989

---

Signif. codes: 0 ‘***’ 0.001 ‘**’ 0.01 ‘*’ 0.05 ‘.’ 0.1 ‘ ’ 1

------------------------------------------------------------------

Sigma link function: log

Sigma Coefficients:

Estimate Std. Error t value Pr(>|t|)

(Intercept) 1.54578 0.09622 16.06 <2e-16 ***

------------------------------------------------------------------

No. of observations in the fit: 54

Degrees of Freedom for the fit: 5

Residual Deg. of Freedom: 49

at cycle: 2

Global Deviance: 486.6034

AIC: 496.6034

SBC: 506.5483

******************************************************************

##### Model Comparison

###### AIC(All Previous Models)

|  | df | AIC |
| --- | --- | --- |
| **GA Models** |  |  |
| Persistence.GA.2 | 4 | 469.786522 |
| Persistence.GA | 6 | 471.144609 |
| **BCTo Models** |  |  |
| Persistence.BCTo.2 | 7 | 472.255443 |
| Persistence.BCTo | 8 | 472.382229 |
| **WEI Models** |  |  |
| Persistence.WEI.2 | 5 | 477.261367 |
| Persistence.WEI | 6 | 478.48115 |
| **LOGNO Models** |  |  |
| Persistence.LOGNO.2 | 5 | 496.603369 |
| Persistence.LOGNO | 6 | 780.468216 |

## Contact Latency Models

### Dogs

#### Response Variable Distribution Checks

###### Dogs.Latency.Distribution$fit

| Distribution | AIC | Errors in Plot? |
| --- | --- | --- |
| IGAMMA | 25.89524136 | No |
| exGAUS | 27.12539013 | No |
| GG | 27.14205944 | No |
| IG | 27.26266424 | No |
| BCCGo | 27.42606623 | No |
| BCCG | 27.42606623 | No |
| LOGNO2 | 27.44045258 | No |
| LOGNO | 27.44045258 | No |
| GIG | 27.89524136 | No |
| GB2 | 29.14238404 | Yes |
| BCT | 29.42606623 | No |
| BCTo | 29.42610463 | Yes |
| GA | 30.01443782 | No |

##### Density plots of distributions used in final models


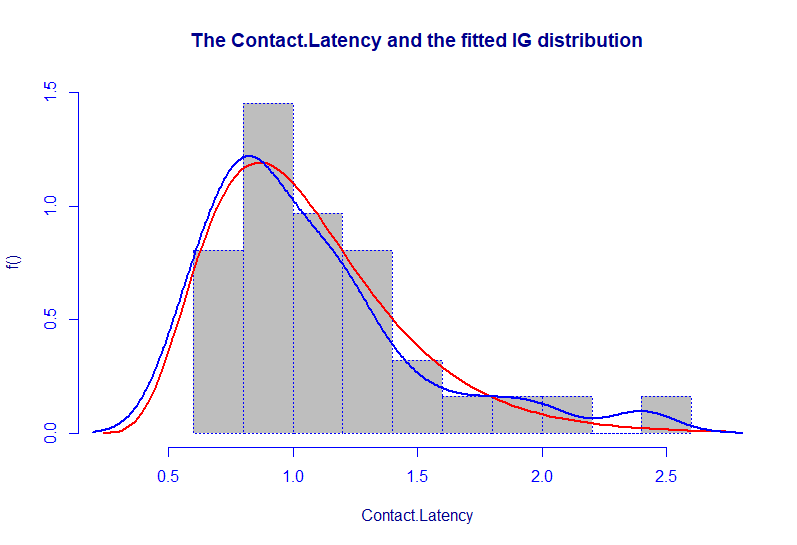

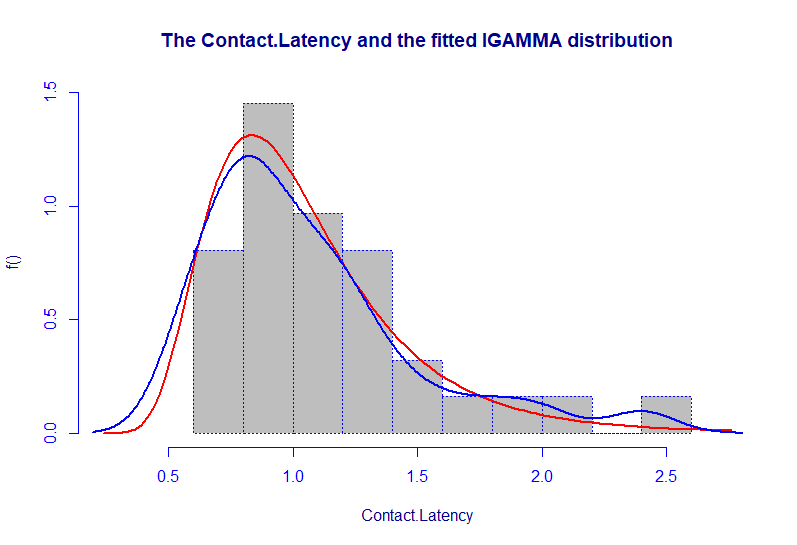


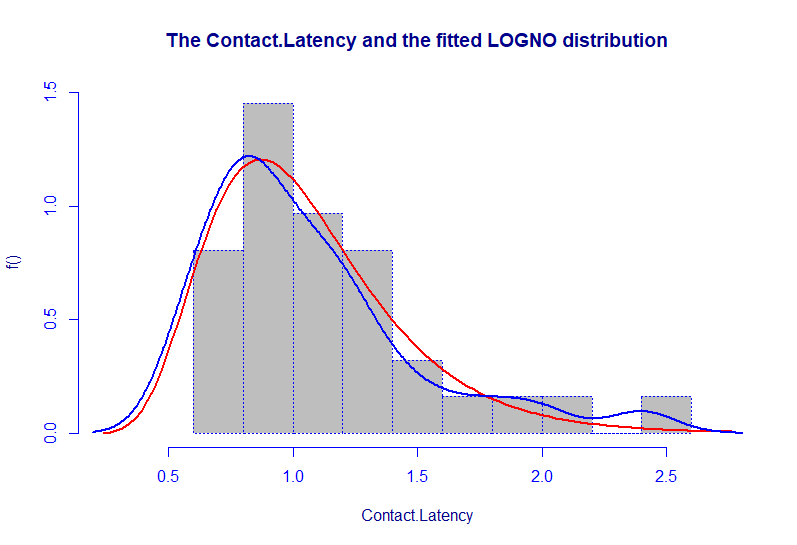

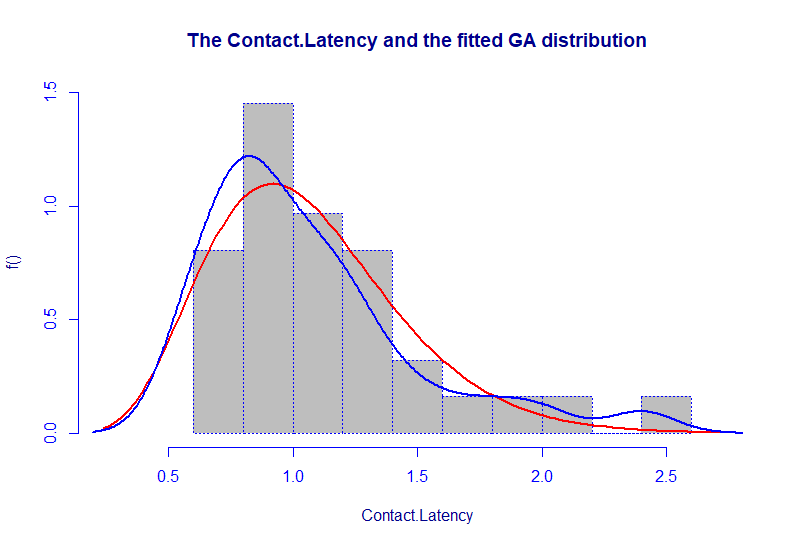


#### Model Distribution Selection

Latency.Dogs.DISTRIBUTION <- gamlss(Contact.Latency ~ Object*Approach.Posture +

Object*Persistence + Age, random = ~1|Individual,

family = "DISTRIBUTION", data = dogs)

| Model | df | AIC |
| --- | --- | --- |
| Latency.Dogs.IG | 8 | 22.2139762 |
| Latency.Dogs.IGAMMA | 8 | 23.9994415 |
| Latency.Dogs.LOGNO | 8 | 24.452884 |
| Latency.Dogs.GA | 8 | 25.0946606 |
| Latency.Dogs.GG | 9 | 25.7231161 |
| Latency.Dogs.BCCGo | 9 | 25.8781709 |
| Latency.Dogs.GIG | 9 | 26.0003484 |
| Latency.Dogs.BCCG | 9 | 27.2859465 |
| Latency.Dogs.BCT | 10 | 29.2858833 |

##### Model Plots

###### plot(Latency.Dogs.IG) & wp(Latency.Dogs.IG)


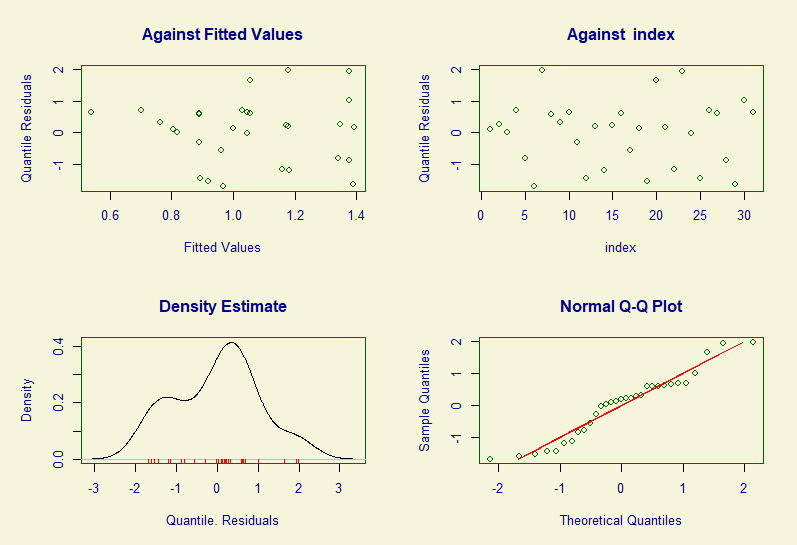

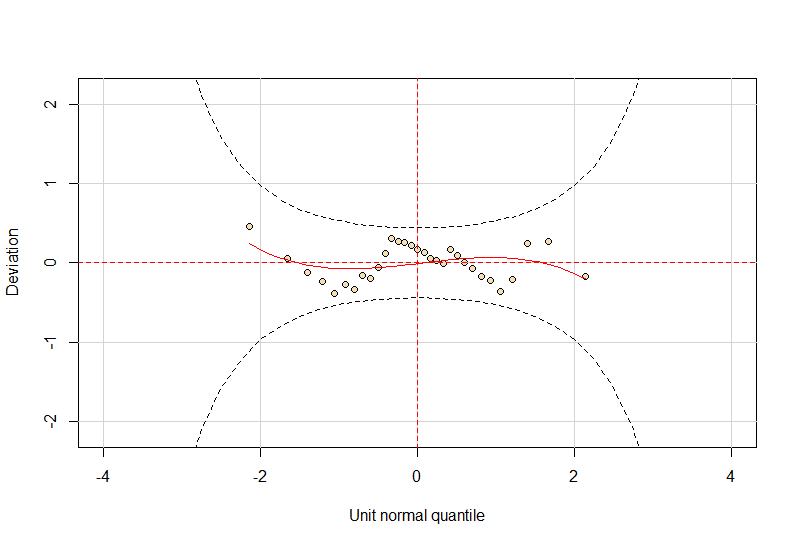


###### plot(Latency.Dogs.IGAMMA) & wp(Latency.Dogs.IGAMMA)


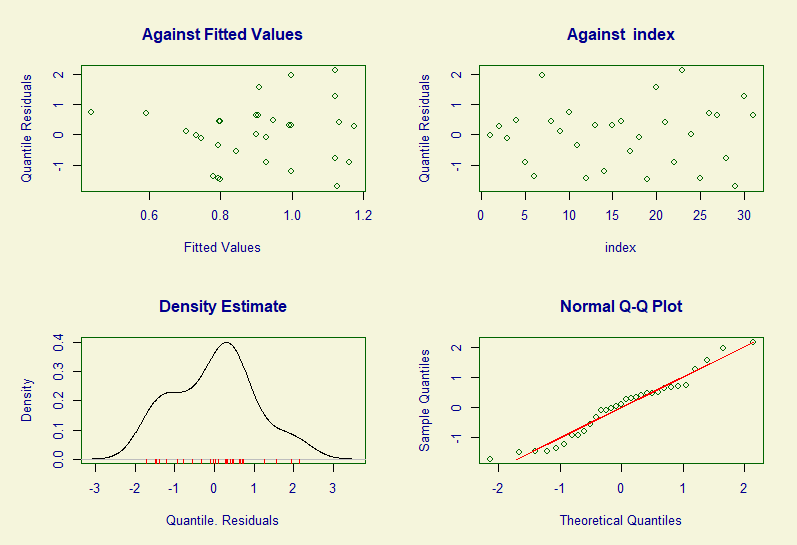

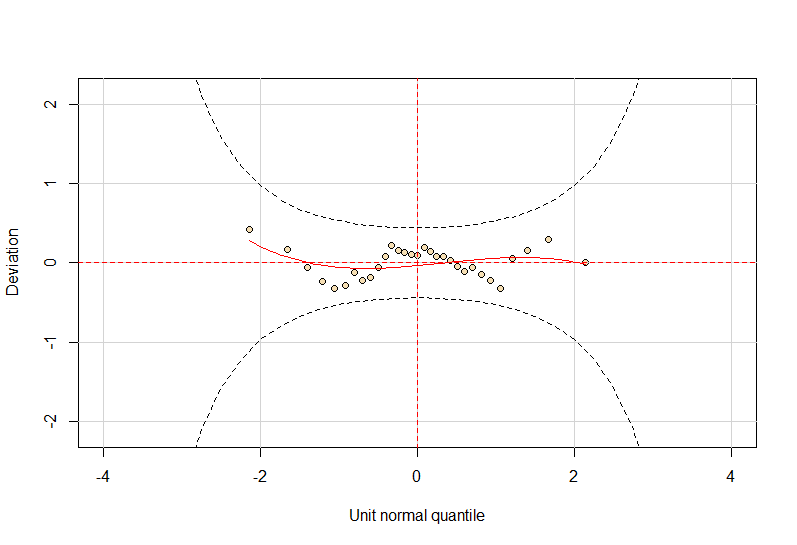


###### plot(Latency.Dogs.LOGNO) & wp(Latency.Dogs.LOGNO)


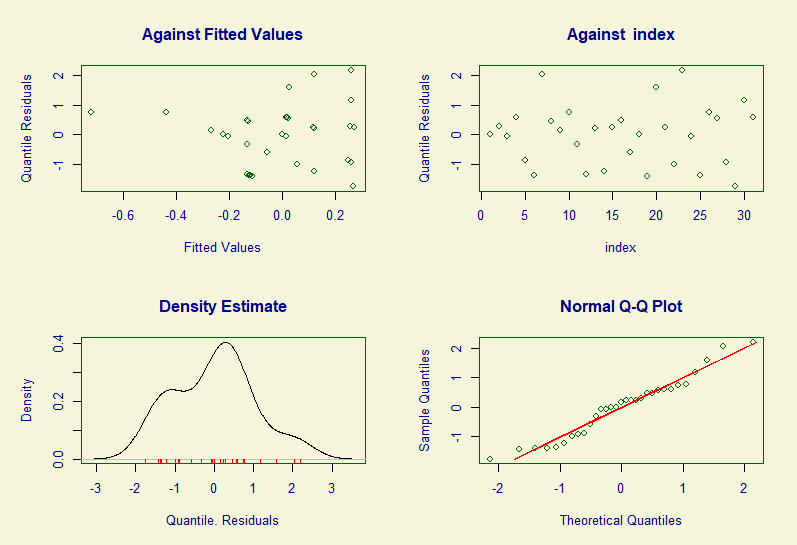

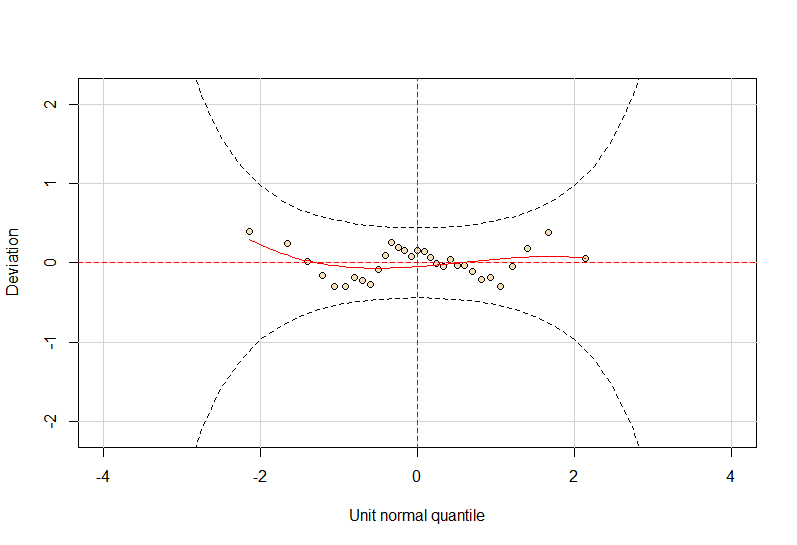


###### plot(Latency.Dogs.GA) & wp(Latency.Dogs.GA)


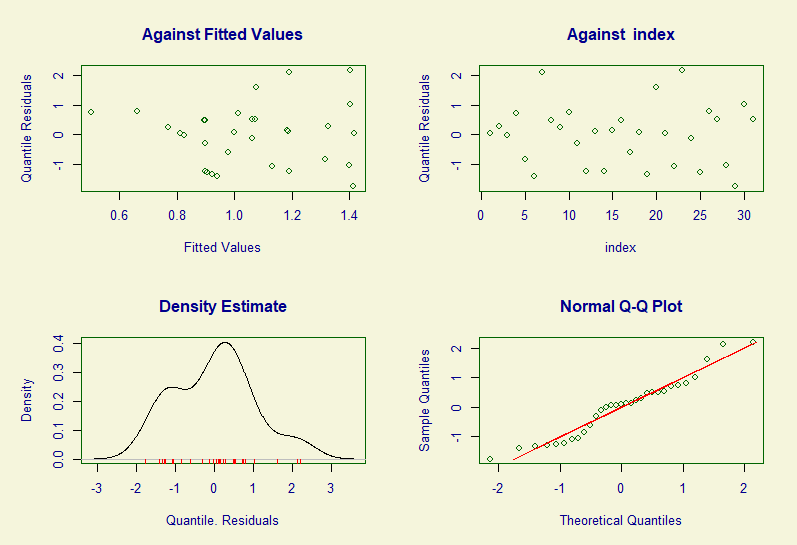

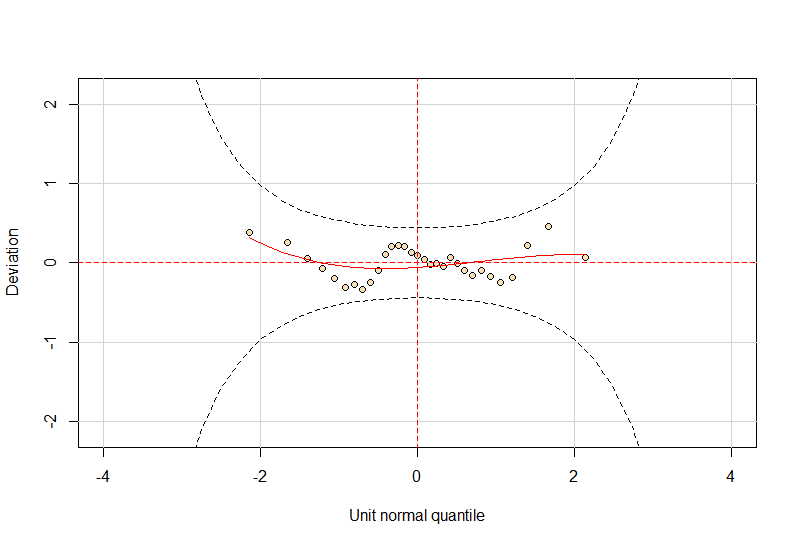


###### plot(Latency.Dogs.GG) & wp(Latency.Dogs.GG)


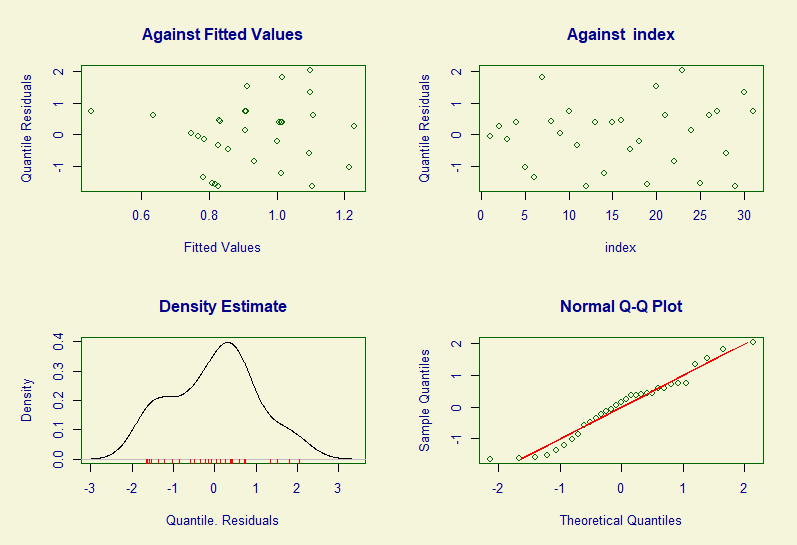

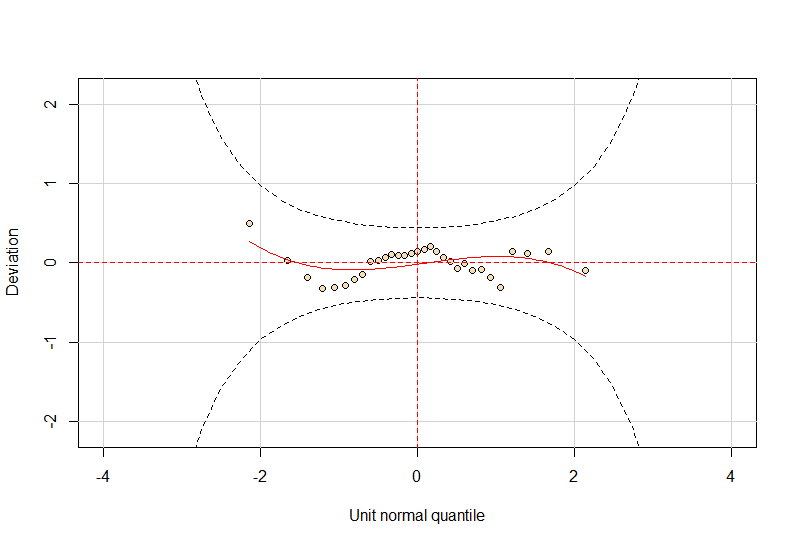


###### plot(Latency.Dogs.BCCGo) & wp(Latency.Dogs.BCCGo)


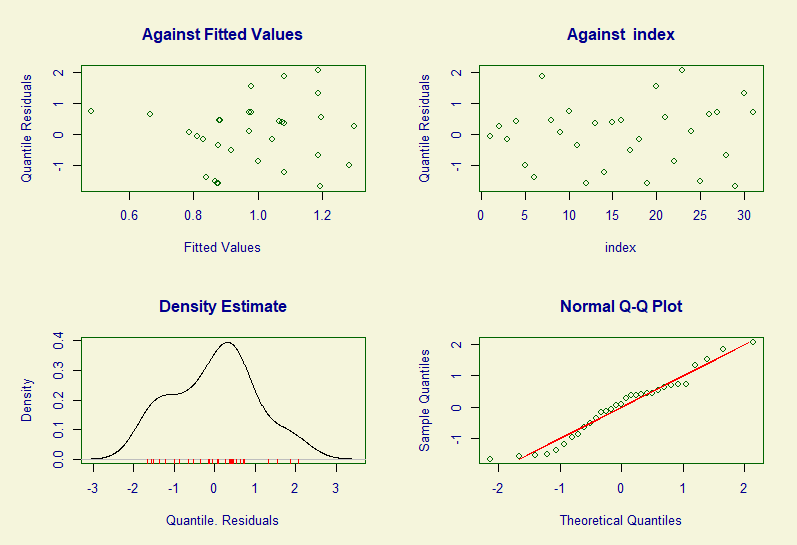

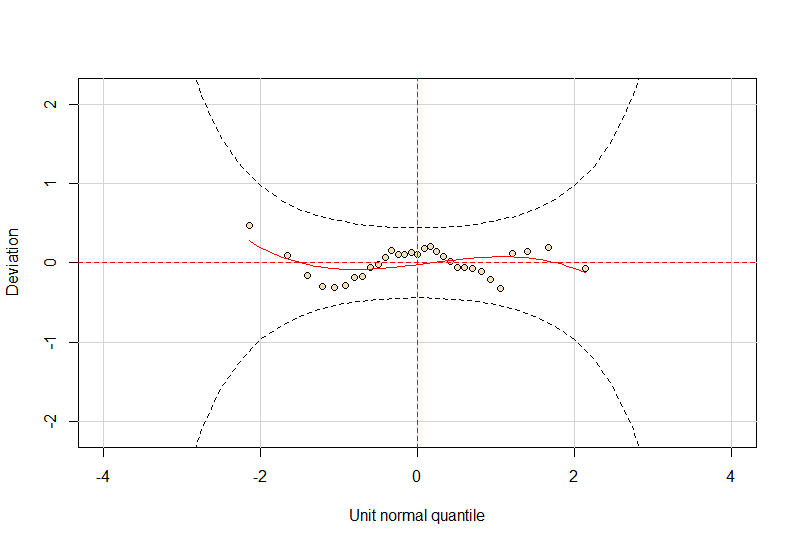


###### plot(Latency.Dogs.GIG) & wp(Latency.Dogs.GIG)


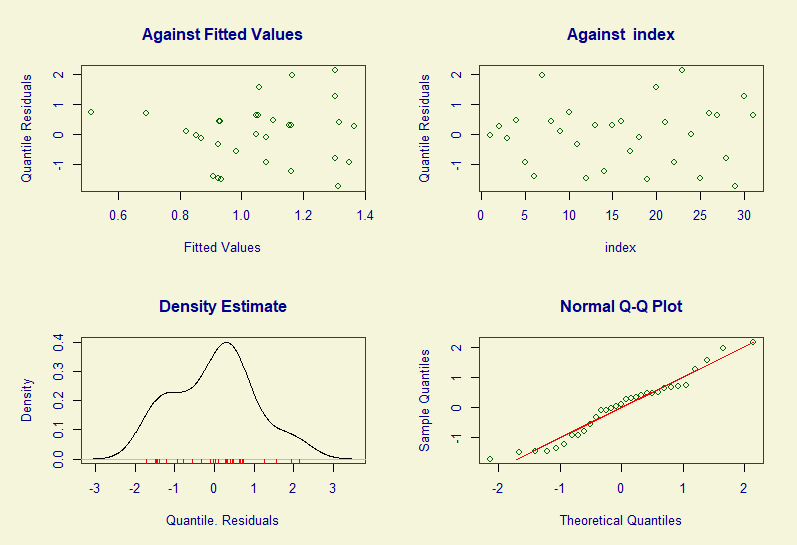

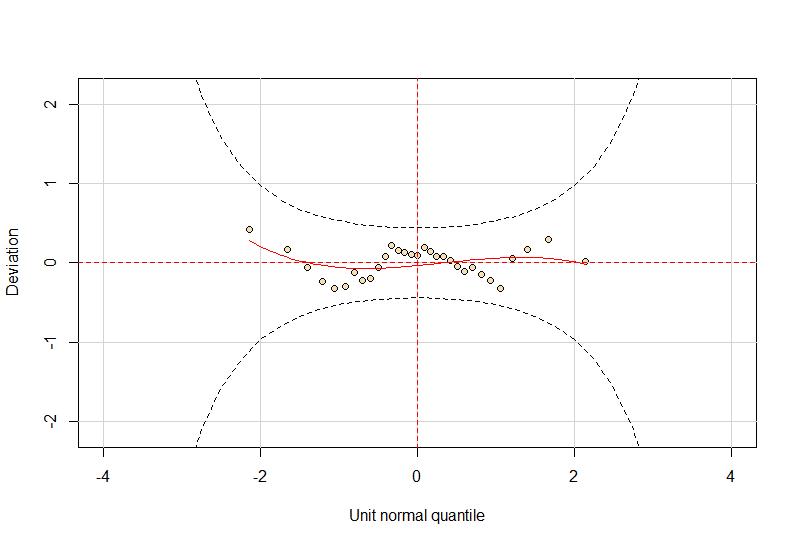


###### plot(Latency.Dogs.BCCG) & wp(Latency.Dogs.BCCG)


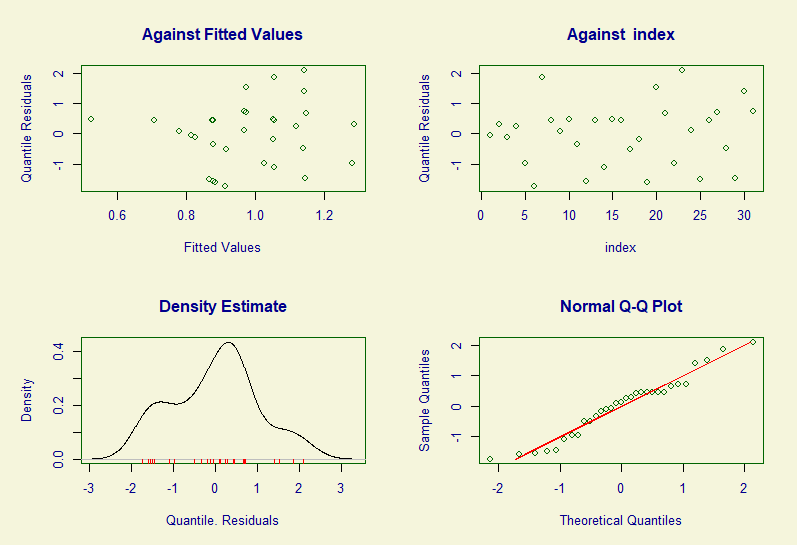

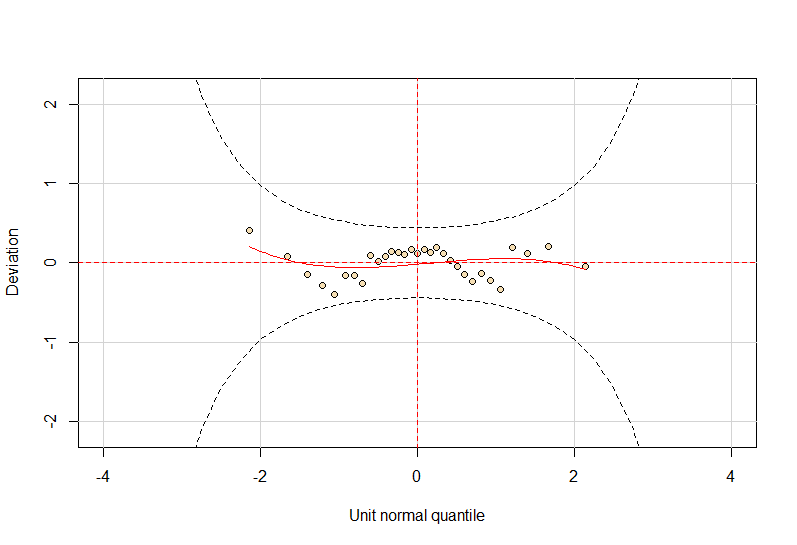


###### plot(Latency.Dogs.BCT) & wp(Latency.Dogs.BCT)


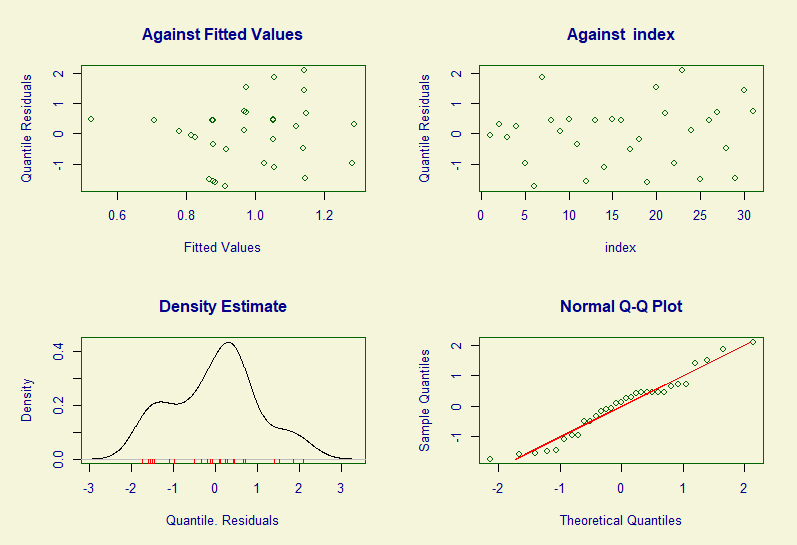

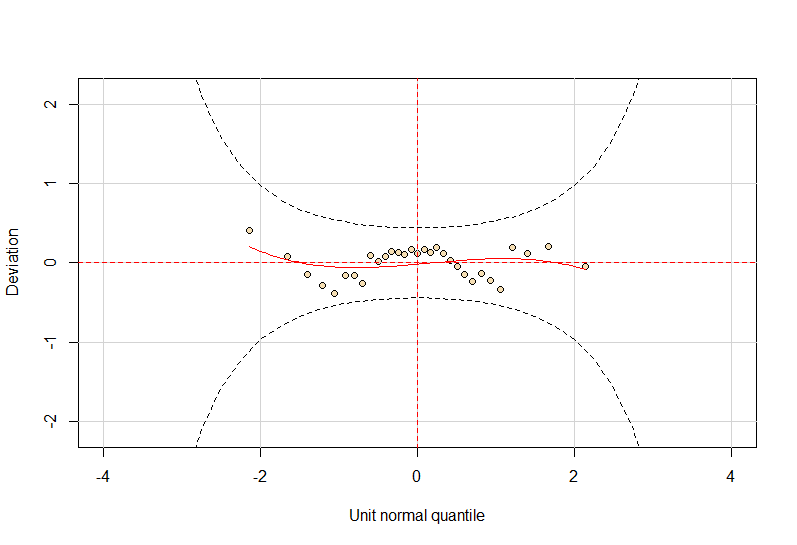


#### Model Reduction and Validation

##### Inverse Gaussian

###### summary(Latency.Dogs.IG)

******************************************************************

Family: c("IG", "Inverse Gaussian")

Call: gamlss(formula = Contact.Latency ~ Object * Approach.Posture +

Object * Persistence + Age, family = "IG",

data = na.omit(dogs), control = con, random = ~1 | Individual)

Fitting method: RS()

------------------------------------------------------------------

Mu link function: log

Mu Coefficients:

Estimate Std. Error t value Pr(>|t|)

(Intercept) 0.4102365 0.1781549 2.303 0.03068 *

ObjectPipe 0.1569040 0.1185724 1.323 0.19875

Approach.PostureUnsure 0.1349085 0.1920899 0.702 0.48953

Persistence -0.0012333 0.0005199 -2.372 0.02641 *

Age -0.1026588 0.0360592 -2.847 0.00913 **

ObjectPipe:Approach.PostureUnsure -0.4556678 0.3444472 -1.323 0.19887

ObjectPipe:Persistence 0.0009844 0.0005155 1.910 0.06872 .

---

Signif. codes: 0 ‘***’ 0.001 ‘**’ 0.01 ‘*’ 0.05 ‘.’ 0.1 ‘ ’ 1

------------------------------------------------------------------

Sigma link function: log

Sigma Coefficients:

Estimate Std. Error t value Pr(>|t|)

(Intercept) -1.3017 0.1529 -8.515 1.45e-08 ***

------------------------------------------------------------------

No. of observations in the fit: 31

Degrees of Freedom for the fit: 8

Residual Deg. of Freedom: 23

at cycle: 2

Global Deviance: 6.213976

AIC: 22.21398

SBC: 33.68587

******************************************************************

###### dropterm(Latency.Dogs.IG, test = "Chisq")

Single term deletions for mu

Model:

Contact.Latency ~ Object * Approach.Posture + Object * Persistence + Age

Df AIC LRT Pr(Chi)

<none> 22.214

Age 1 27.960 7.7464 0.005382 **

Object:Approach.Posture 1 21.622 1.4077 0.235436

Object:Persistence 1 22.384 2.1705 0.140683

---

Signif. codes: 0 ‘***’ 0.001 ‘**’ 0.01 ‘*’ 0.05 ‘.’ 0.1 ‘ ’ 1

###### summary(Latency.Dogs.IG.2)

******************************************************************

Family: c("IG", "Inverse Gaussian")

Call: gamlss(formula = Contact.Latency ~ Object * Persistence + Age +

Approach.Posture, family = "IG", data = na.omit(dogs),

control = con, random = ~1 | Individual)

Fitting method: RS()

------------------------------------------------------------------

Mu link function: log

Mu Coefficients:

Estimate Std. Error t value Pr(>|t|)

(Intercept) 0.4136302 0.1807401 2.289 0.0312 *

ObjectPipe 0.1216014 0.1180047 1.030 0.3131

Persistence -0.0012998 0.0005321 -2.443 0.0223 *

Age -0.0993958 0.0364335 -2.728 0.0117 *

Approach.PostureUnsure 0.0448015 0.1716762 0.261 0.7963

ObjectPipe:Persistence 0.0010621 0.0005275 2.013 0.0554 .

---

Signif. codes: 0 ‘***’ 0.001 ‘**’ 0.01 ‘*’ 0.05 ‘.’ 0.1 ‘ ’ 1

------------------------------------------------------------------

Sigma link function: log

Sigma Coefficients:

Estimate Std. Error t value Pr(>|t|)

(Intercept) -1.279 0.151 -8.47 1.13e-08 ***

---

Signif. codes: 0 ‘***’ 0.001 ‘**’ 0.01 ‘*’ 0.05 ‘.’ 0.1 ‘ ’ 1

------------------------------------------------------------------

No. of observations in the fit: 31

Degrees of Freedom for the fit: 7

Residual Deg. of Freedom: 24

at cycle: 2

Global Deviance: 7.621691

AIC: 21.62169

SBC: 31.6596

******************************************************************

###### dropterm(Latency.Dogs.IG.2, test = "Chisq")

Single term deletions for mu

Model:

Contact.Latency ~ Object * Persistence + Age + Approach.Posture

Df AIC LRT Pr(Chi)

<none> 21.622

Age 1 26.747 7.1255 0.0076 **

Approach.Posture 1 19.692 0.0700 0.7914

Object:Persistence 1 21.992 2.3701 0.1237

---

Signif. codes: 0 ‘***’ 0.001 ‘**’ 0.01 ‘*’ 0.05 ‘.’ 0.1 ‘ ’ 1

###### summary(Latency.Dogs.IG.3)

******************************************************************

Family: c("IG", "Inverse Gaussian")

Call: gamlss(formula = Contact.Latency ~ Object + Persistence + Age +

Approach.Posture, family = "IG", data = na.omit(dogs),

control = con, random = ~1 | Individual)

Fitting method: RS()

------------------------------------------------------------------

Mu link function: log

Mu Coefficients:

Estimate Std. Error t value Pr(>|t|)

(Intercept) 3.989e-01 1.866e-01 2.138 0.042479 *

ObjectPipe 1.729e-01 1.200e-01 1.441 0.162116

Persistence -2.827e-04 6.492e-05 -4.354 0.000199 ***

Age -1.058e-01 3.708e-02 -2.853 0.008580 **

Approach.PostureUnsure 7.570e-02 1.773e-01 0.427 0.673021

---

Signif. codes: 0 ‘***’ 0.001 ‘**’ 0.01 ‘*’ 0.05 ‘.’ 0.1 ‘ ’ 1

------------------------------------------------------------------

Sigma link function: log

Sigma Coefficients:

Estimate Std. Error t value Pr(>|t|)

(Intercept) -1.2408 0.1529 -8.114 1.81e-08 ***

------------------------------------------------------------------

No. of observations in the fit: 31

Degrees of Freedom for the fit: 6

Residual Deg. of Freedom: 25

at cycle: 2

Global Deviance: 9.991834

AIC: 21.99183

SBC: 30.59576

******************************************************************

##### Inverse Gamma

###### summary(Latency.Dogs.IGAMMA)

******************************************************************

Family: c("IGAMMA", "Inverse Gamma")

Call: gamlss(formula = Contact.Latency ~ Object * Approach.Posture +

Object * Persistence + Age, family = "IGAMMA",

data = na.omit(dogs), control = con, random = ~1 | Individual)

Fitting method: RS()

------------------------------------------------------------------

Mu link function: log

Mu Coefficients:

Estimate Std. Error t value Pr(>|t|)

(Intercept) 0.1943433 0.1590614 1.222 0.2342

ObjectPipe 0.1157691 0.1168878 0.990 0.3323

Approach.PostureUnsure 0.1614497 0.1827590 0.883 0.3862

Persistence -0.0015549 0.0008241 -1.887 0.0719 .

Age -0.0813011 0.0313085 -2.597 0.0161 *

ObjectPipe:Approach.PostureUnsure -0.3494135 0.3383956 -1.033 0.3125

ObjectPipe:Persistence 0.0012806 0.0008414 1.522 0.1416

---

Signif. codes: 0 ‘***’ 0.001 ‘**’ 0.01 ‘*’ 0.05 ‘.’ 0.1 ‘ ’ 1

------------------------------------------------------------------

Sigma link function: log

Sigma Coefficients:

Estimate Std. Error t value Pr(>|t|)

(Intercept) -1.2911 0.1288 -10.02 7.32e-10 ***

------------------------------------------------------------------

No. of observations in the fit: 31

Degrees of Freedom for the fit: 8

Residual Deg. of Freedom: 23

at cycle: 9

Global Deviance: 7.999442

AIC: 23.99944

SBC: 35.47134

******************************************************************

###### dropterm(Latency.Dogs.IGAMMA, test = "Chisq")

Single term deletions for mu

Model:

Contact.Latency ~ Object * Approach.Posture + Object * Persistence + Age

Df AIC LRT Pr(Chi)

<none> 23.999

Age 1 28.092 6.0927 0.01357 *

Object:Approach.Posture 1 23.092 1.0925 0.29591

Object:Persistence 1 24.297 2.2973 0.12960

---

Signif. codes: 0 ‘***’ 0.001 ‘**’ 0.01 ‘*’ 0.05 ‘.’ 0.1 ‘ ’ 1

###### summary(Latency.Dogs.IGAMMA.2)

******************************************************************

Family: c("IGAMMA", "Inverse Gamma")

Call: gamlss(formula = Contact.Latency ~ Object * Persistence + Age +

Approach.Posture, family = "IGAMMA", data = na.omit(dogs),

control = con, random = ~1 | Individual)

Fitting method: RS()

------------------------------------------------------------------

Mu link function: log

Mu Coefficients:

Estimate Std. Error t value Pr(>|t|)

(Intercept) 0.2058006 0.1613666 1.275 0.2144

ObjectPipe 0.0718711 0.1108730 0.648 0.5230

Persistence -0.0016423 0.0008340 -1.969 0.0606 .

Age -0.0792909 0.0318927 -2.486 0.0203 *

Approach.PostureUnsure 0.0565357 0.1593793 0.355 0.7259

ObjectPipe:Persistence 0.0013911 0.0008498 1.637 0.1147

---

Signif. codes: 0 ‘***’ 0.001 ‘**’ 0.01 ‘*’ 0.05 ‘.’ 0.1 ‘ ’ 1

------------------------------------------------------------------

Sigma link function: log

Sigma Coefficients:

Estimate Std. Error t value Pr(>|t|)

(Intercept) -1.2739 0.1287 -9.902 5.96e-10 ***

---

No. of observations in the fit: 31

Degrees of Freedom for the fit: 7

Residual Deg. of Freedom: 24

at cycle: 9

Global Deviance: 9.091991

AIC: 23.09199

SBC: 33.1299

******************************************************************

###### dropterm(Latency.Dogs.IGAMMA.2, test = "Chisq")

Single term deletions for mu

Model:

Contact.Latency ~ Object * Persistence + Age + Approach.Posture

Df AIC LRT Pr(Chi)

<none> 23.092

Age 1 26.725 5.6327 0.01763 *

Approach.Posture 1 21.216 0.1242 0.72456

Object:Persistence 1 23.766 2.6737 0.10202

---

Signif. codes: 0 ‘***’ 0.001 ‘**’ 0.01 ‘*’ 0.05 ‘.’ 0.1 ‘ ’ 1

###### summary(Latency.Dogs.IGAMMA.3)

******************************************************************

Family: c("IGAMMA", "Inverse Gamma")

Call: gamlss(formula = Contact.Latency ~ Object + Persistence + Age +

Approach.Posture, family = "IGAMMA",

data = na.omit(dogs), control = con, random = ~1 | Individual)

Fitting method: RS()

------------------------------------------------------------------

Mu link function: log

Mu Coefficients:

Estimate Std. Error t value Pr(>|t|)

(Intercept) 0.1415034 0.1646664 0.859 0.3983

ObjectPipe 0.1429034 0.1076359 1.328 0.1963

Persistence -0.0003477 0.0001859 -1.871 0.0732 .

Age -0.0797352 0.0332547 -2.398 0.0243 *

Approach.PostureUnsure 0.0879124 0.1650867 0.533 0.5991

---

Signif. codes: 0 ‘***’ 0.001 ‘**’ 0.01 ‘*’ 0.05 ‘.’ 0.1 ‘ ’ 1

------------------------------------------------------------------

Sigma link function: log

Sigma Coefficients:

Estimate Std. Error t value Pr(>|t|)

(Intercept) -1.2319 0.1279 -9.635 6.76e-10 ***

------------------------------------------------------------------

No. of observations in the fit: 31

Degrees of Freedom for the fit: 6

Residual Deg. of Freedom: 25

at cycle: 9

Global Deviance: 11.76571

AIC: 23.76571

SBC: 32.36963

******************************************************************

##### Log-Normal

###### summary(Latency.Dogs.LOGNO)

******************************************************************

Family: c("LOGNO", "Log Normal")

Call: gamlss(formula = Contact.Latency ~ Object * Approach.Posture +

Object * Persistence + Age, family = "LOGNO", data = na.omit(dogs),

control = con, random = ~1 | Individual)

Fitting method: RS()

------------------------------------------------------------------

Mu link function: identity

Mu Coefficients:

Estimate Std. Error t value Pr(>|t|)

(Intercept) 0.3378984 0.1625735 2.078 0.0490 *

ObjectPipe 0.1412920 0.1184929 1.192 0.2453

Approach.PostureUnsure 0.1363869 0.1853760 0.736 0.4693

Persistence -0.0015129 0.0008023 -1.886 0.0720 .

Age -0.0908627 0.0326460 -2.783 0.0106 *

ObjectPipe:Approach.PostureUnsure -0.3975069 0.3451327 -1.152 0.2613

ObjectPipe:Persistence 0.0012175 0.0008265 1.473 0.1543

---

Signif. codes: 0 ‘***’ 0.001 ‘**’ 0.01 ‘*’ 0.05 ‘.’ 0.1 ‘ ’ 1

------------------------------------------------------------------

Sigma link function: log

Sigma Coefficients:

Estimate Std. Error t value Pr(>|t|)

(Intercept) -1.271 0.127 -10.01 7.5e-10 ***

------------------------------------------------------------------

No. of observations in the fit: 31

Degrees of Freedom for the fit: 8

Residual Deg. of Freedom: 23

at cycle: 2

Global Deviance: 8.452884

AIC: 24.45288

SBC: 35.92478

******************************************************************

###### dropterm(Latency.Dogs.LOGNO, test = "Chisq")

Single term deletions for mu

Model:

Contact.Latency ~ Object * Approach.Posture + Object * Persistence + Age

Df AIC LRT Pr(Chi)

<none> 24.453

Age 1 29.368 6.9147 0.008549 **

Object:Approach.Posture 1 23.752 1.2989 0.254408

Object:Persistence 1 24.550 2.0970 0.147588

---

Signif. codes: 0 ‘***’ 0.001 ‘**’ 0.01 ‘*’ 0.05 ‘.’ 0.1 ‘ ’ 1

###### summary(Latency.Dogs.LOGNO.2)

******************************************************************

Family: c("LOGNO", "Log Normal")

Call: gamlss(formula = Contact.Latency ~ Object * Persistence + Age +

Approach.Posture, family = "LOGNO", data = na.omit(dogs),

control = con, random = ~1 | Individual)

Fitting method: RS()

------------------------------------------------------------------

Mu link function: identity

Mu Coefficients:

Estimate Std. Error t value Pr(>|t|)

(Intercept) 0.3517489 0.1655606 2.125 0.0441 *

ObjectPipe 0.0953582 0.1139420 0.837 0.4109

Persistence -0.0016021 0.0008155 -1.965 0.0611 .

Age -0.0884757 0.0332699 -2.659 0.0137 *

Approach.PostureUnsure 0.0277571 0.1629674 0.170 0.8662

ObjectPipe:Persistence 0.0013275 0.0008384 1.583 0.1264

---

Signif. codes: 0 ‘***’ 0.001 ‘**’ 0.01 ‘*’ 0.05 ‘.’ 0.1 ‘ ’ 1

------------------------------------------------------------------

Sigma link function: log

Sigma Coefficients:

Estimate Std. Error t value Pr(>|t|)

(Intercept) -1.250 0.127 -9.845 6.66e-10 ***

------------------------------------------------------------------

No. of observations in the fit: 31

Degrees of Freedom for the fit: 7

Residual Deg. of Freedom: 24

at cycle: 2

Global Deviance: 9.751817

AIC: 23.75182

SBC: 33.78973

******************************************************************

###### dropterm(Latency.Dogs.LOGNO.2, test = "Chisq")

Single term deletions for mu

Model: Contact.Latency ~ Object * Persistence + Age + Approach.Posture

Df AIC LRT Pr(Chi)

<none> 23.752

Age 1 28.122 6.3703 0.0116 *

Approach.Posture 1 21.781 0.0290 0.8648

Object:Persistence 1 24.163 2.4108 0.1205

---

Signif. codes: 0 ‘***’ 0.001 ‘**’ 0.01 ‘*’ 0.05 ‘.’ 0.1 ‘ ’ 1

###### summary(Latency.Dogs.LOGNO.3)

******************************************************************

Family: c("LOGNO", "Log Normal")

Call: gamlss(formula = Contact.Latency ~ Object + Persistence + Age +

Approach.Posture, family = "LOGNO", data = na.omit(dogs),

control = con, random = ~1 | Individual)

Fitting method: RS()

------------------------------------------------------------------

Mu link function: identity

Mu Coefficients:

Estimate Std. Error t value Pr(>|t|)

(Intercept) 0.3098394 0.1699111 1.824 0.0802 .

ObjectPipe 0.1567504 0.1113900 1.407 0.1717

Persistence -0.0003516 0.0002112 -1.665 0.1085

Age -0.0909106 0.0345520 -2.631 0.0144 *

Approach.PostureUnsure 0.0569946 0.1683378 0.339 0.7378

---

Signif. codes: 0 ‘***’ 0.001 ‘**’ 0.01 ‘*’ 0.05 ‘.’ 0.1 ‘ ’ 1

------------------------------------------------------------------

Sigma link function: log

Sigma Coefficients:

Estimate Std. Error t value Pr(>|t|)

(Intercept) -1.211 0.127 -9.539 8.24e-10 ***

------------------------------------------------------------------

No. of observations in the fit: 31

Degrees of Freedom for the fit: 6

Residual Deg. of Freedom: 25

at cycle: 2

Global Deviance: 12.1626

AIC: 24.1626

SBC: 32.76652

******************************************************************

##### Gamma

###### summary(Latency.Dogs.GA)

******************************************************************

Family: c("GA", "Gamma")

Call: gamlss(formula = Contact.Latency ~ Object * Approach.Posture +

Object * Persistence + Age, family = "GA", data = na.omit(dogs),

control = con, random = ~1 | Individual)

Fitting method: RS()

------------------------------------------------------------------

Mu link function: log

Mu Coefficients:

Estimate Std. Error t value Pr(>|t|)

(Intercept) 0.4196806 0.1644667 2.552 0.01783 *

ObjectPipe 0.1643692 0.1172481 1.402 0.17430

Approach.PostureUnsure 0.1094975 0.1837670 0.596 0.55709

Persistence -0.0014680 0.0007488 -1.960 0.06217 .

Age -0.1026352 0.0336409 -3.051 0.00567 **

ObjectPipe:Approach.PostureUnsure -0.4472226 0.3441880 -1.299 0.20670

ObjectPipe:Persistence 0.0011600 0.0007603 1.526 0.14071

---

Signif. codes: 0 ‘***’ 0.001 ‘**’ 0.01 ‘*’ 0.05 ‘.’ 0.1 ‘ ’ 1

------------------------------------------------------------------

Sigma link function: log

Sigma Coefficients:

Estimate Std. Error t value Pr(>|t|)

(Intercept) -1.2739 0.1301 -9.796 1.13e-09 ***

------------------------------------------------------------------

No. of observations in the fit: 31

Degrees of Freedom for the fit: 8

Residual Deg. of Freedom: 23

at cycle: 2

Global Deviance: 9.094661

AIC: 25.09466

SBC: 36.56656

******************************************************************

###### dropterm(Latency.Dogs.GA, test = "Chisq")

Single term deletions for mu

Model:

Contact.Latency ~ Object * Approach.Posture + Object * Persistence + Age

Df AIC LRT Pr(Chi)

<none> 23.999

Age 1 28.092 6.0927 0.01357 *

Object:Approach.Posture 1 23.092 1.0925 0.29591

Object:Persistence 1 24.297 2.2973 0.12960

---

Signif. codes: 0 ‘***’ 0.001 ‘**’ 0.01 ‘*’ 0.05 ‘.’ 0.1 ‘ ’ 1

###### summary(Latency.Dogs.GA.2)

******************************************************************

Family: c("GA", "Gamma")

Call: gamlss(formula = Contact.Latency ~ Object * Persistence + Age +

Approach.Posture, family = "GA", data = na.omit(dogs),

control = con, random = ~1 | Individual)

Fitting method: RS()

------------------------------------------------------------------

Mu link function: log

Mu Coefficients:

Estimate Std. Error t value Pr(>|t|)

(Intercept) 0.4317947 0.1681393 2.568 0.01688 *

ObjectPipe 0.1182663 0.1147076 1.031 0.31281

Persistence -0.0015582 0.0007647 -2.038 0.05277 .

Age -0.0996764 0.0342883 -2.907 0.00773 **

Approach.PostureUnsure 0.0019261 0.1633738 0.012 0.99069

ObjectPipe:Persistence 0.0012676 0.0007755 1.635 0.11518

---

Signif. codes: 0 ‘***’ 0.001 ‘**’ 0.01 ‘*’ 0.05 ‘.’ 0.1 ‘ ’ 1

------------------------------------------------------------------

Sigma link function: log

Sigma Coefficients:

Estimate Std. Error t value Pr(>|t|)

(Intercept) -1.2496 0.1297 -9.636 1.01e-09 ***

------------------------------------------------------------------

No. of observations in the fit: 31

Degrees of Freedom for the fit: 7

Residual Deg. of Freedom: 24

at cycle: 2

Global Deviance: 10.64324

AIC: 24.64324

SBC: 34.68115

******************************************************************

###### dropterm(Latency.Dogs.GA.2, test = "Chisq")

Single term deletions for mu

Model: Contact.Latency ~ Object * Persistence + Age + Approach.Posture

Df AIC LRT Pr(Chi)

<none> 24.643

Age 1 30.183 7.5400 0.006034 **

Approach.Posture 1 22.643 0.0001 0.990230

Object:Persistence 1 24.863 2.2196 0.136270

---

Signif. codes: 0 ‘***’ 0.001 ‘**’ 0.01 ‘*’ 0.05 ‘.’ 0.1 ‘ ’ 1

###### summary(Latency.Dogs.GA.3)

******************************************************************

Family: c("GA", "Gamma")

Call: gamlss(formula = Contact.Latency ~ Object + Persistence + Age +

Approach.Posture, family = "GA", data = na.omit(dogs),

control = con, random = ~1 | Individual)

Fitting method: RS()

------------------------------------------------------------------

Mu link function: log

Mu Coefficients:

Estimate Std. Error t value Pr(>|t|)

(Intercept) 0.4038062 0.1724769 2.341 0.02749 *

ObjectPipe 0.1706946 0.1134002 1.505 0.14479

Persistence -0.0003501 0.0001603 -2.185 0.03851 *

Age -0.1037697 0.0352672 -2.942 0.00693 **

Approach.PostureUnsure 0.0296296 0.1678586 0.177 0.86131

---

Signif. codes: 0 ‘***’ 0.001 ‘**’ 0.01 ‘*’ 0.05 ‘.’ 0.1 ‘ ’ 1

------------------------------------------------------------------

Sigma link function: log

Sigma Coefficients:

Estimate Std. Error t value Pr(>|t|)

(Intercept) -1.2148 0.1296 -9.375 1.16e-09 ***

------------------------------------------------------------------

No. of observations in the fit: 31

Degrees of Freedom for the fit: 6

Residual Deg. of Freedom: 25

at cycle: 2

Global Deviance: 12.86282

AIC: 24.86282

SBC: 33.46674

******************************************************************

##### Model AIC Values & Plots

|  | **df** | **AIC** |
| --- | --- | --- |
| **IG Models** |  |  |
| Latency.Dogs.IG.2 | 7 | 21.6216907 |
| Latency.Dogs.IG.3 | 6 | 21.991834 |
| Latency.Dogs.IG | 8 | 22.2139762 |
| **IGAMMA Models** |  |  |
| Latency.Dogs.IGAMMA.2 | 7 | 23.0919912 |
| Latency.Dogs.IGAMMA.3 | 6 | 23.7657082 |
| Latency.Dogs.IGAMMA | 8 | 23.9994415 |
| **LOGNO Models** |  |  |
| Latency.Dogs.LOGNO.2 | 7 | 23.751817 |
| Latency.Dogs.LOGNO.3 | 6 | 24.162601 |
| Latency.Dogs.LOGNO | 8 | 24.452884 |
| **GA Models** |  |  |
| Latency.Dogs.GA.2 | 7 | 24.6432376 |
| Latency.Dogs.GA.3 | 6 | 24.8628194 |
| Latency.Dogs.GA | 8 | 25.0946606 |

#### Plots


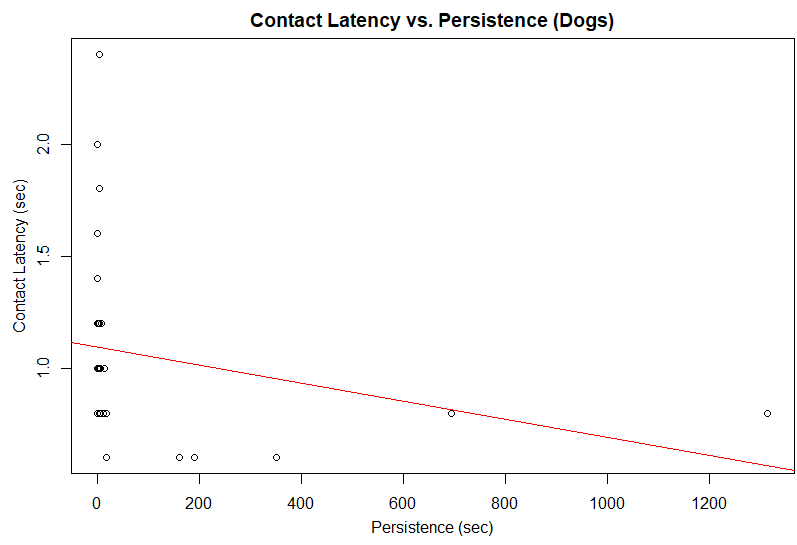


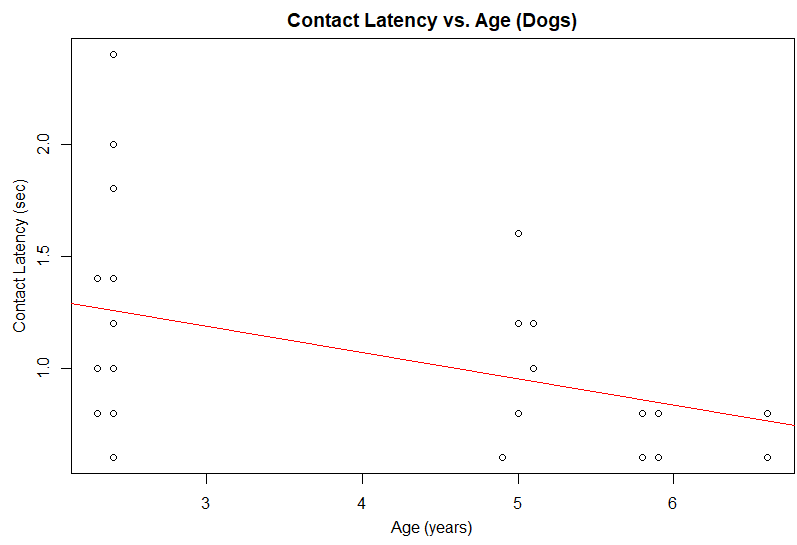


### Wolves

#### Response Variable Distribution Checks

###### Wolves.Latency.Distribution$fit

| Distribution | AIC | Errors in Plot? |
| --- | --- | --- |
| BCPEo | 42.5956468 | No |
| BCPE | 45.0343444 | Yes |
| GA | 46.7551277 | No |
| WEI | 47.8204512 | No |
| WEI3 | 47.8204512 | No |
| WEI2 | 47.8204512 | No |
| LOGNO2 | 48.007022 | No |
| LOGNO | 48.007022 | No |
| exGAUS | 48.1292579 | No |
| BCCGo | 48.6930046 | No |
| BCCG | 48.6930046 | No |
| GG | 48.7442016 | No |
| GIG | 48.7551277 | No |
| IG | 49.2806389 | No |
| GB2 | 50.2402167 | Yes |
| BCT | 50.3641552 | No |
| BCTo | 50.3641552 | No |
| IGAMMA | 52.8433712 | Yes |
| EXP | 58.0239751 | No |

##### Density plots of distributions used in final models


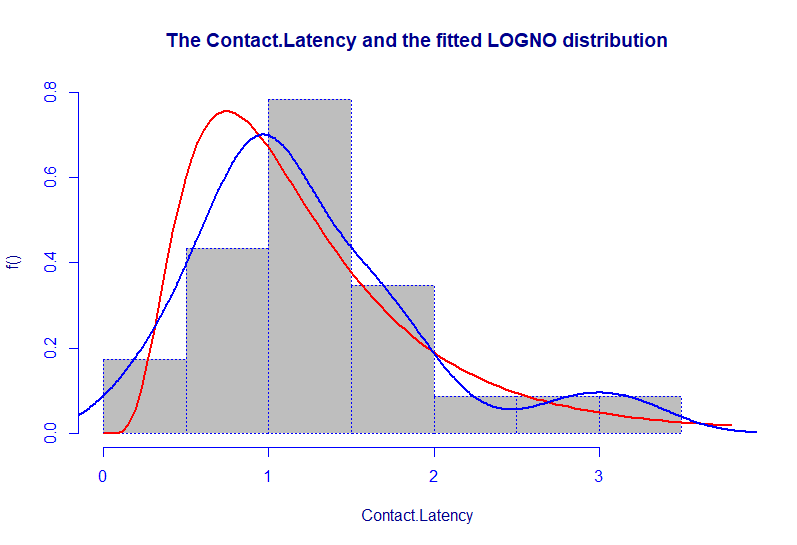

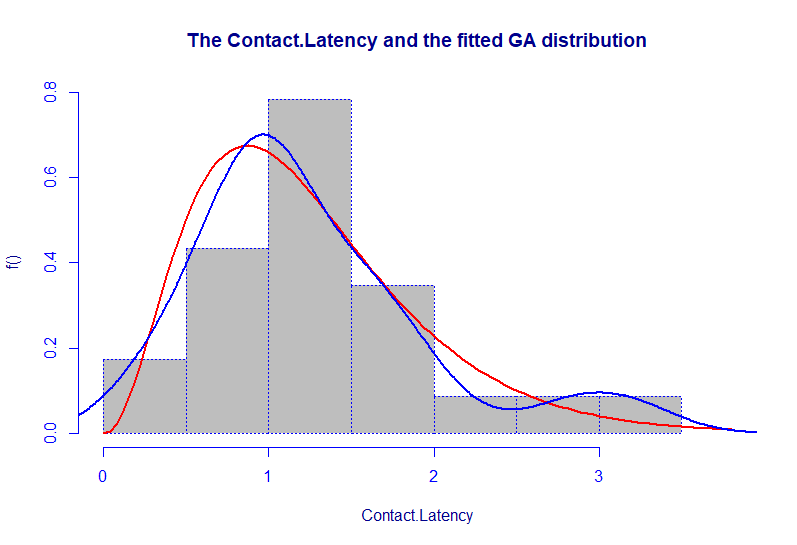


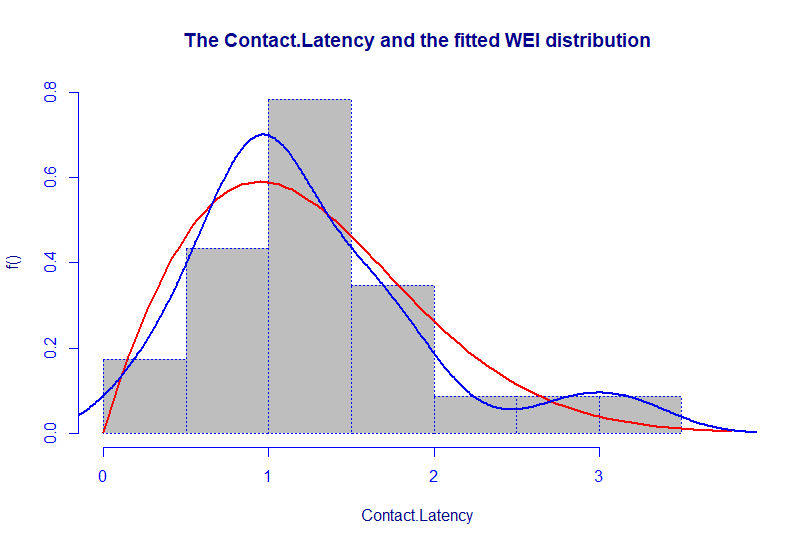

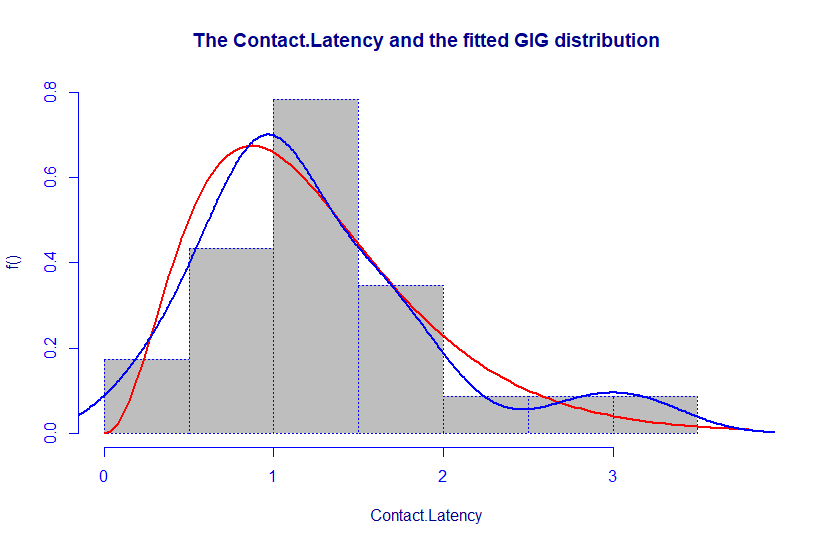


#### Model Distribution Selection

Latency.Wolves.DISTRIBUTION <- gamlss(Contact.Latency ~ Object*Approach.Posture +

Object*Persistence + Age, random = ~1|Individual,

family = "DISTRIBUTION", data = wolves)

| Model | df | AIC | Residuals outside CI |
| --- | --- | --- | --- |
| Latency.Wolves.BCPEo | 9 | 37.4157307 | 14 |
| Latency.Wolves.LOGNO | 7 | 46.3634629 | 0 |
| Latency.Wolves.GA | 7 | 46.5175795 | 0 |
| Latency.Wolves.WEI | 7 | 46.9785802 | 0 |
| Latency.Wolves.BCCG | 8 | 47.1128132 | 0 |
| Latency.Wolves.GIG | 8 | 48.0492091 | 0 |
| Latency.Wolves.BCCGo | 8 | 48.3522457 | 0 |
| Latency.Wolves.BCT | 9 | 49.1125839 | 0 |
| Latency.Wolves.BCTo | 9 | 50.3524066 |  |
| Latency.Wolves.IG | 7 | 50.4243689 | 0 |

##### Model Plots

###### plot(Latency.Wolves.BCPEo) & wp(Latency.Wolves.BCPEo)


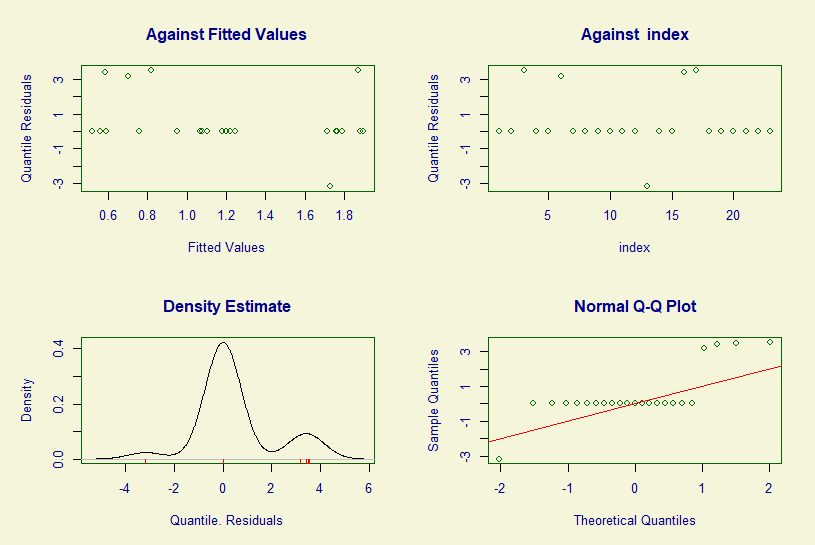

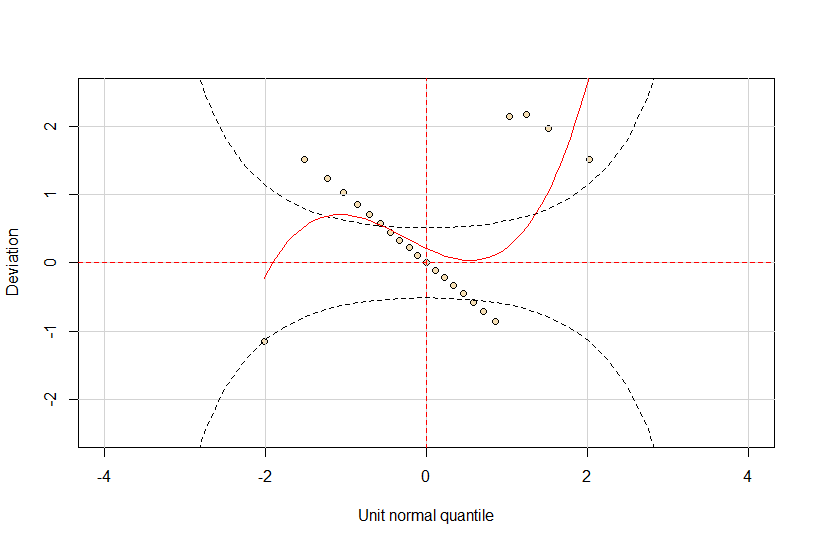


###### plot(Latency.Wolves.LOGNO) & wp(Latency.Wolves.LOGNO)


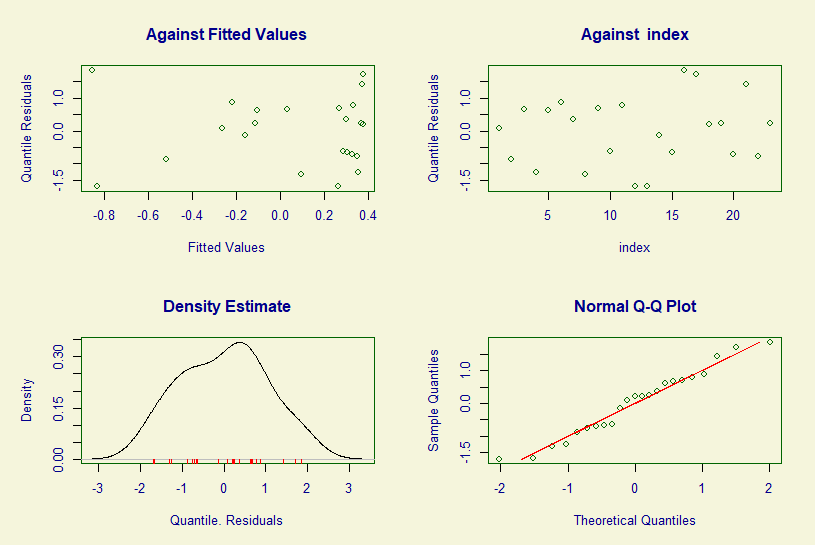

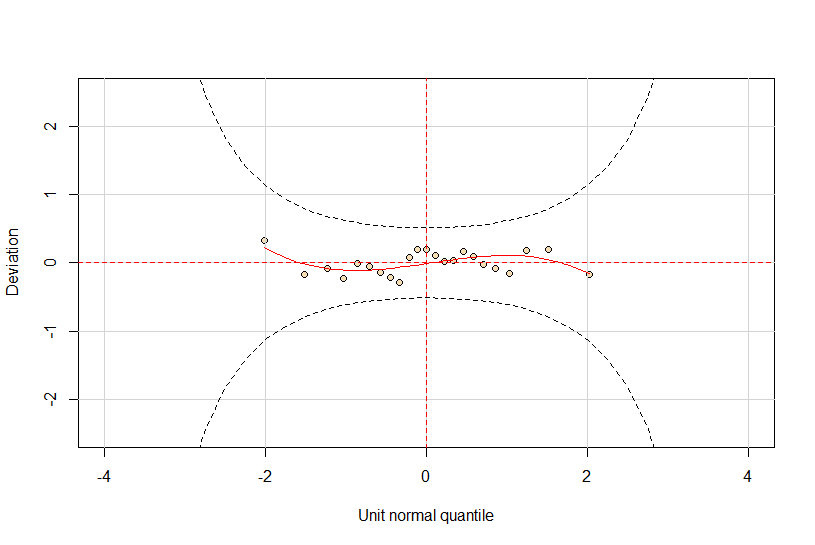


###### plot(Latency.Wolves.GA) & wp(Latency.Wolves.GA)


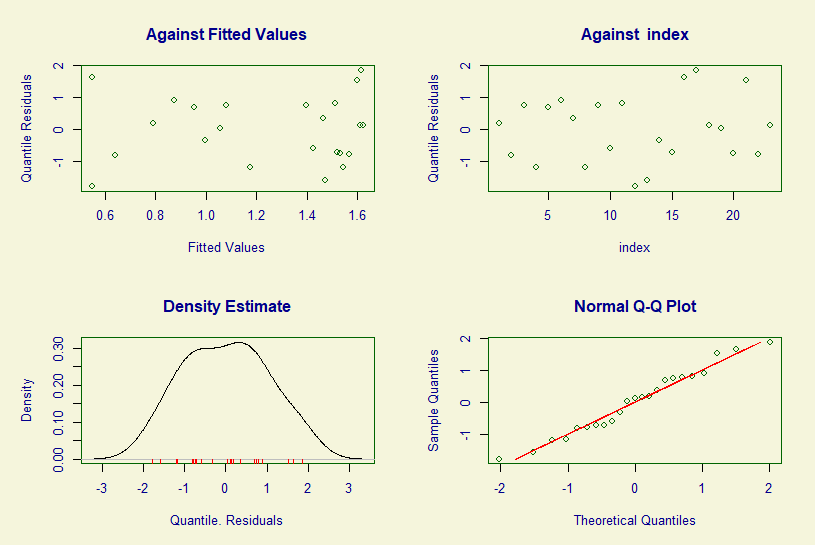

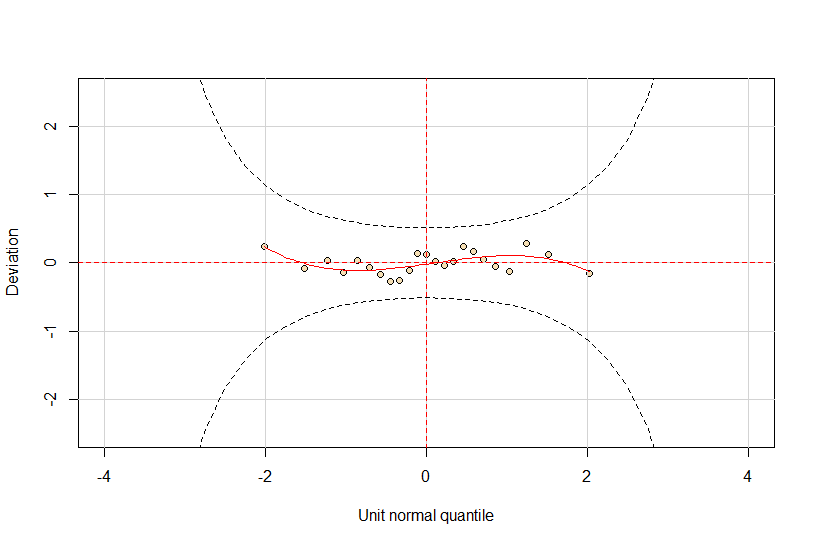


###### plot(Latency.Wolves.WEI) & wp(Latency.Wolves.WEI)


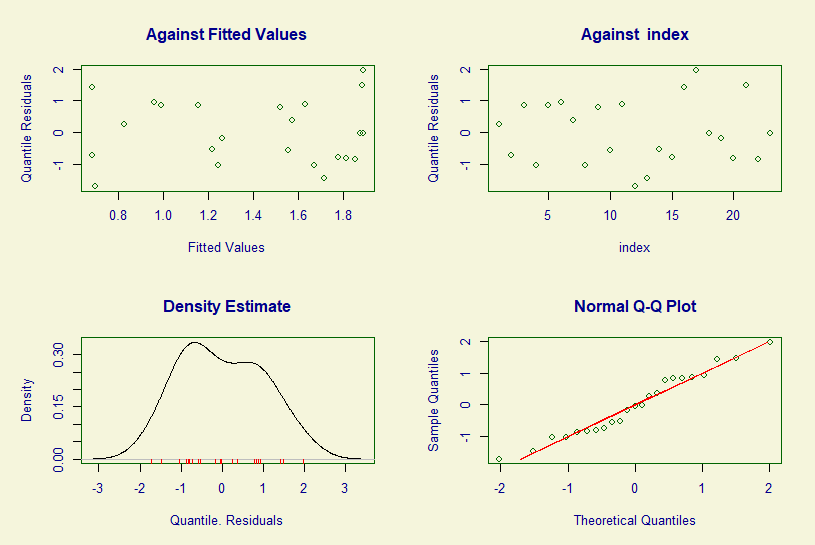

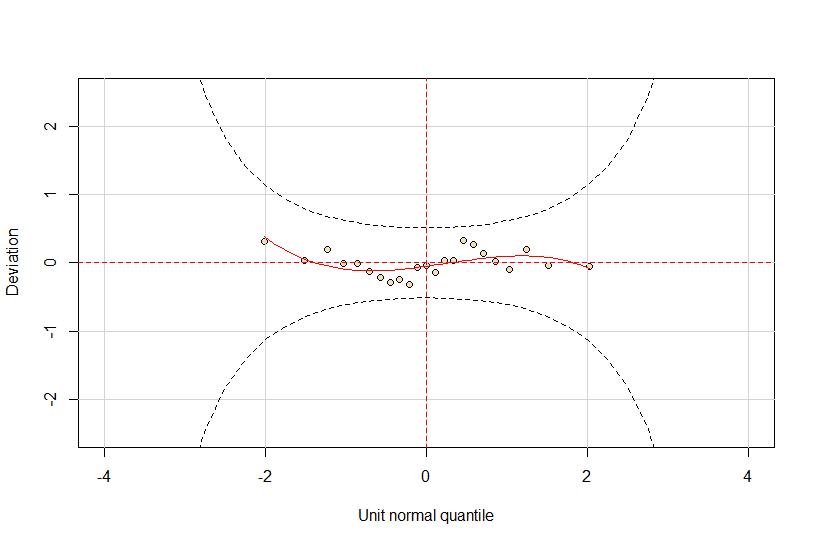


###### plot(Latency.Wolves.BCCG) & wp(Latency.Wolves.BCCG)


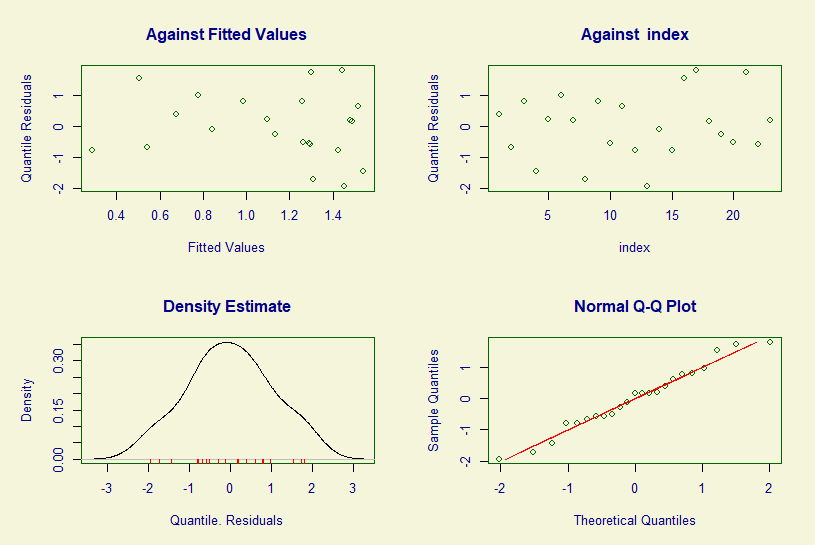

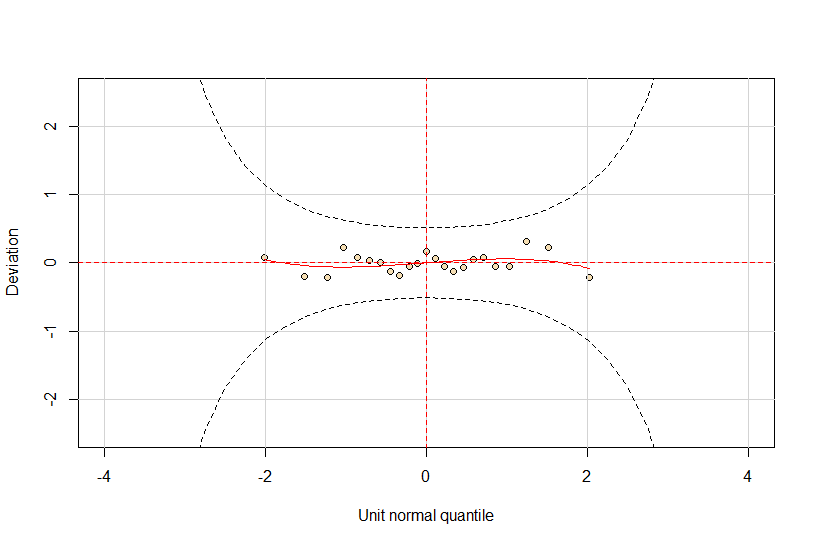


###### plot(Latency.Wolves.GIG) & wp(Latency.Wolves.GIG)


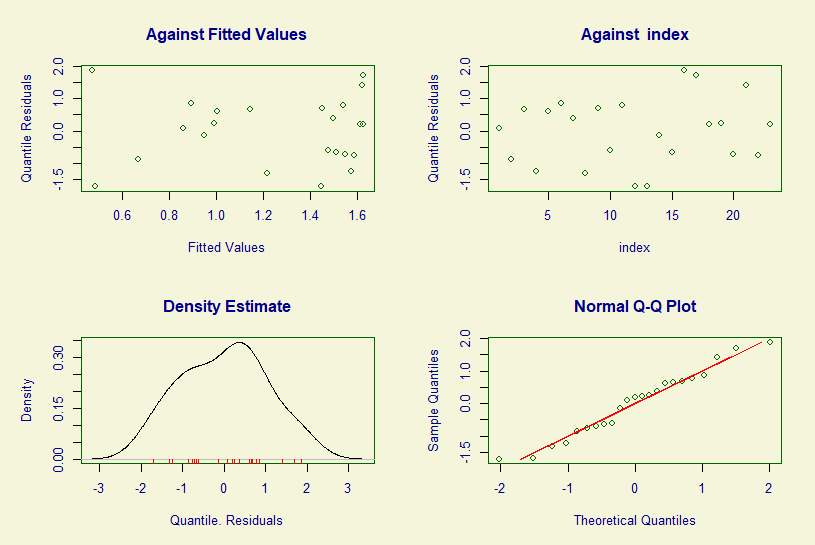

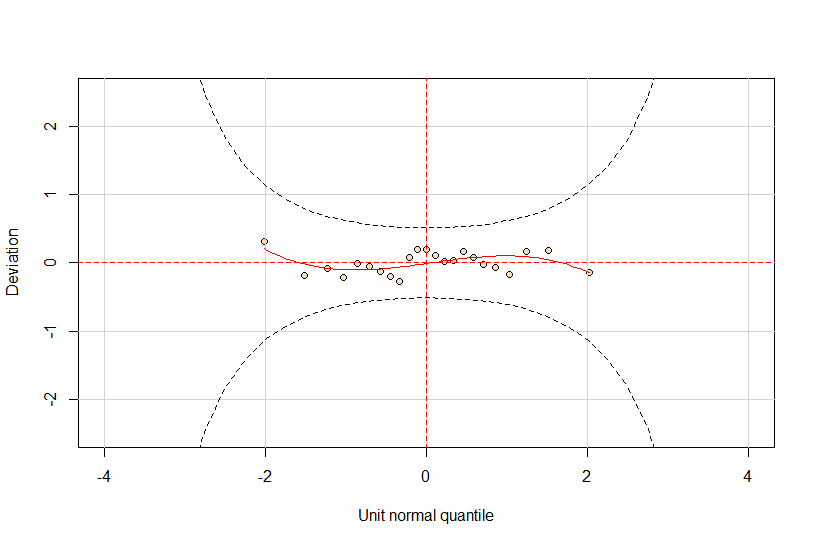


###### plot(Latency.Wolves.BCCGo) & wp(Latency.Wolves.BCCGo)


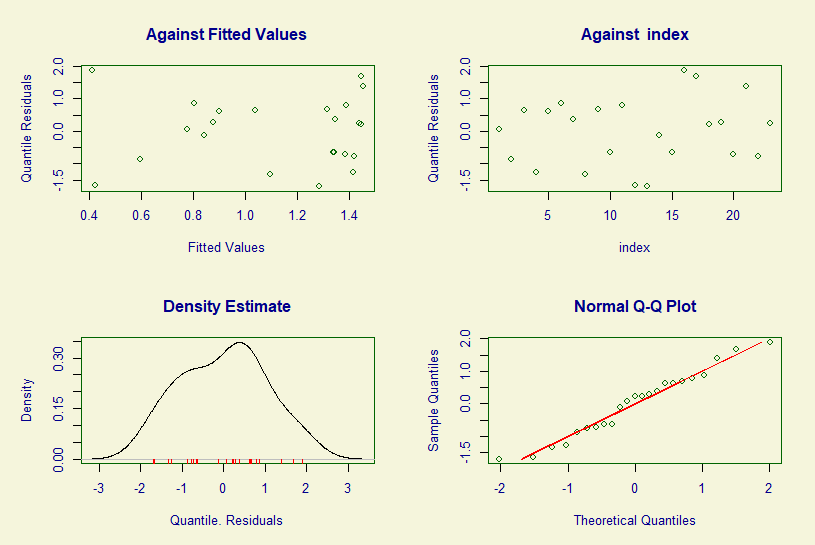

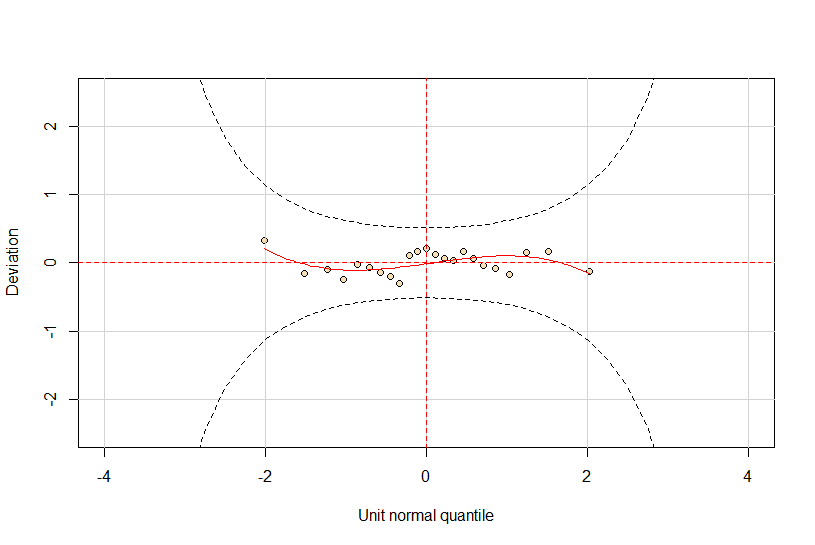


###### plot(Latency.Wolves.BCT) & wp(Latency.Wolves.BCT)


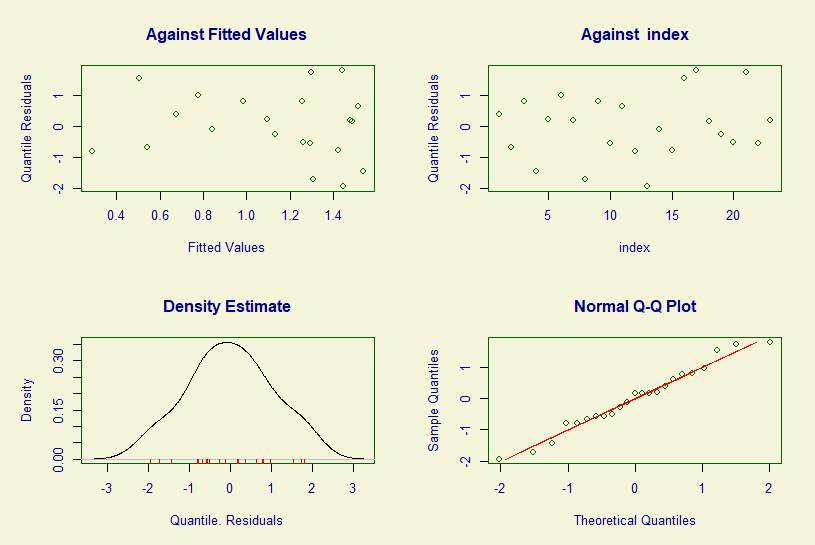

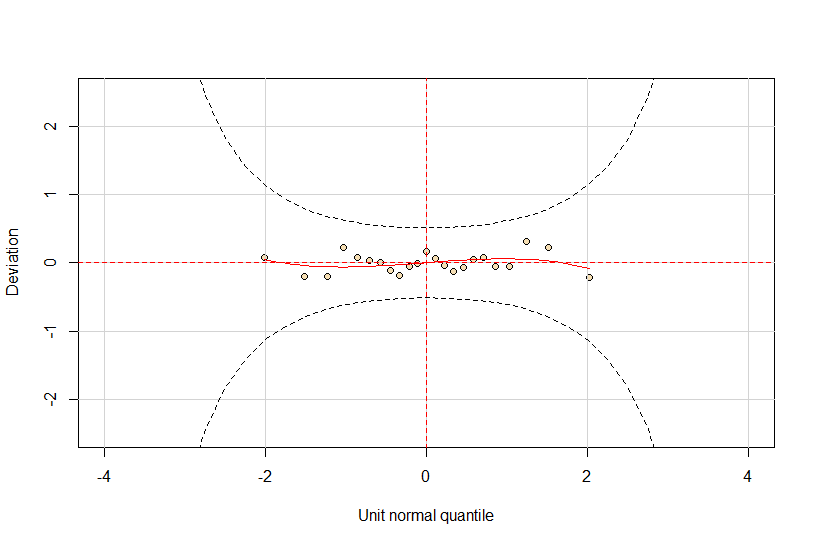


###### plot(Latency.Wolves.BCTo) & wp(Latency.Wolves.BCTo)


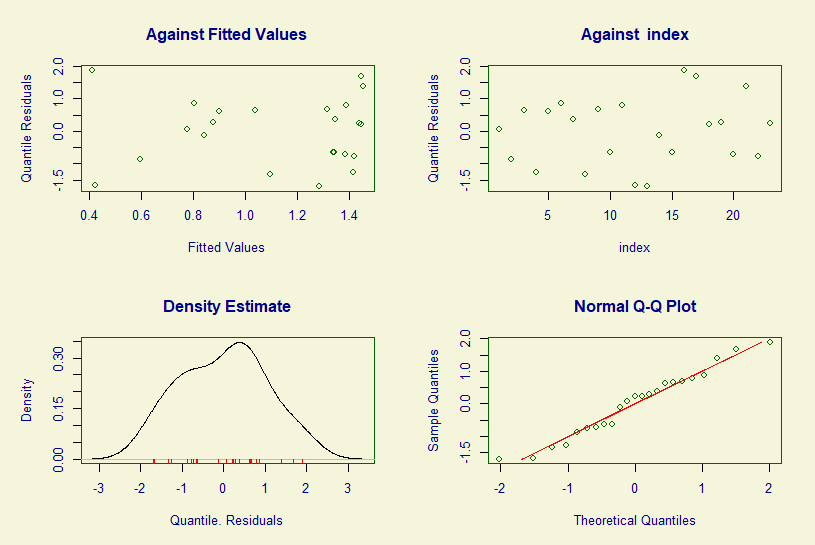

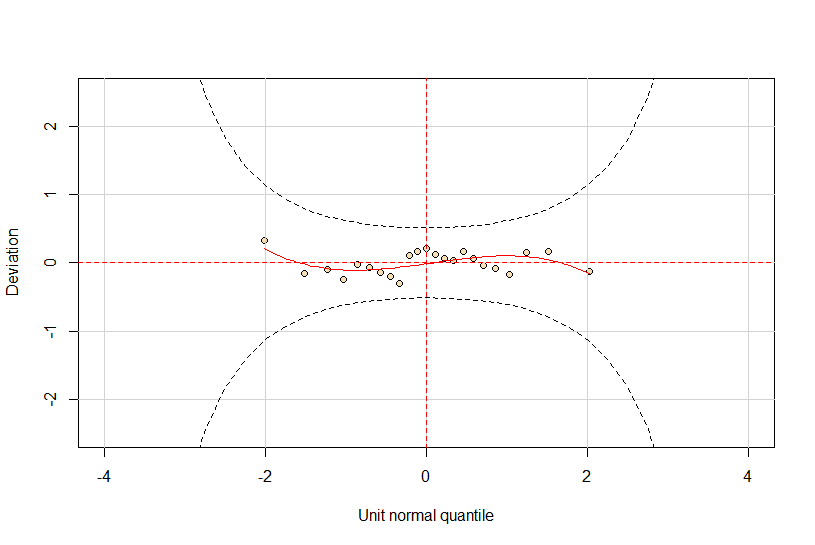


###### plot(Latency.Wolves.IG) & wp(Latency.Wolves.IG)


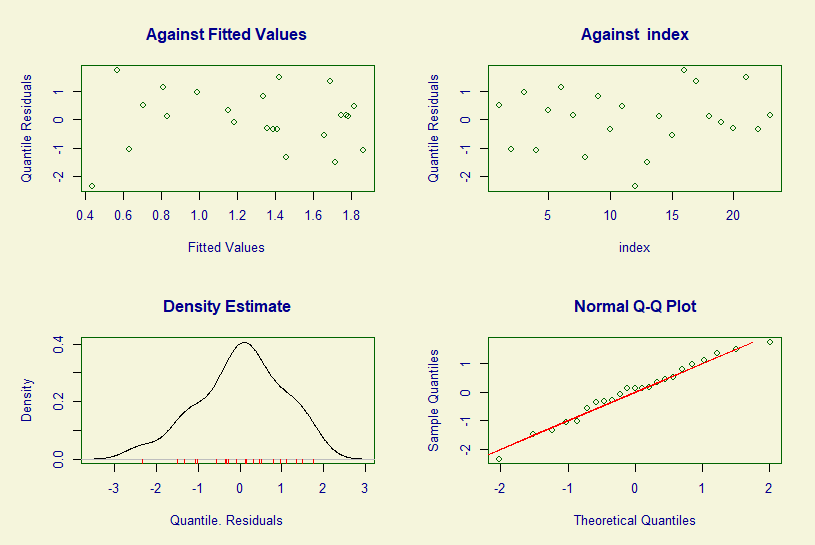

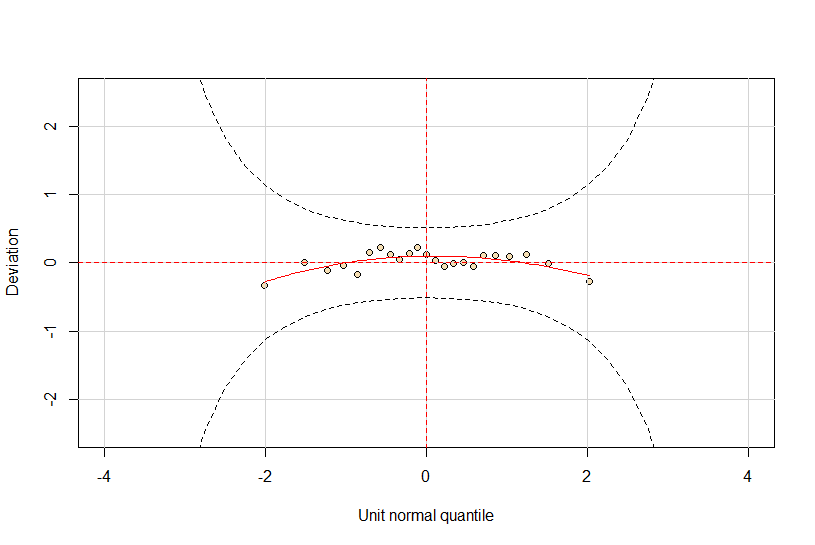


#### Model Reduction and Validation

##### Log-Normal

###### summary(Latency.Wolves.LOGNO)

******************************************************************

Family: c("LOGNO", "Log Normal")

Call: gamlss(formula = Contact.Latency ~ Object * Approach.Posture +

Object * Persistence + Age, family = "LOGNO", data = na.omit(wolves),

control = con, random = ~1 | Individual)

Fitting method: RS()

------------------------------------------------------------------

Mu link function: identity

Mu Coefficients:

Estimate Std. Error t value Pr(>|t|)

(Intercept) 3.934e-01 4.354e-01 0.904 0.380

ObjectPipe -1.605e-02 2.839e-01 -0.057 0.956

Approach.PostureUnsure -6.026e-01 3.719e-01 -1.621 0.125

Persistence -6.340e-04 4.472e-04 -1.418 0.176

Age 3.605e-05 5.844e-02 0.001 1.000

ObjectPipe:Persistence -5.450e-04 5.707e-04 -0.955 0.354

------------------------------------------------------------------

Sigma link function: log

Sigma Coefficients:

Estimate Std. Error t value Pr(>|t|)

(Intercept) -0.7749 0.1474 -5.255 7.85e-05 ***

---

Signif. codes: 0 ‘***’ 0.001 ‘**’ 0.01 ‘*’ 0.05 ‘.’ 0.1 ‘ ’ 1

------------------------------------------------------------------

No. of observations in the fit: 23

Degrees of Freedom for the fit: 7

Residual Deg. of Freedom: 16

at cycle: 2

Global Deviance: 32.36346

AIC: 46.36346

SBC: 54.31192

******************************************************************

###### dropterm(Latency.Wolves.LOGNO, test = "Chisq")

Single term deletions for mu

Model: Contact.Latency ~ Object * Approach.Posture + Object * Persistence + Age

Df AIC LRT Pr(Chi)

<none> 46.363

Age 1 44.363 0.00000 0.9995

Object:Approach.Posture 0 46.363 0.00000

Object:Persistence 1 45.258 0.89422 0.3443

###### summary(Latency.Wolves.LOGNO.2)

******************************************************************

Family: c("LOGNO", "Log Normal")

Call: gamlss(formula = Contact.Latency ~ Object * Persistence + Approach.Posture +

Age, family = "LOGNO", data = na.omit(wolves),

control = con, random = ~1 | Individual)

Fitting method: RS()

------------------------------------------------------------------

Mu link function: identity

Mu Coefficients:

Estimate Std. Error t value Pr(>|t|)

(Intercept) 3.934e-01 4.354e-01 0.904 0.380

ObjectPipe -1.605e-02 2.839e-01 -0.057 0.956

Persistence -6.340e-04 4.472e-04 -1.418 0.176

Approach.PostureUnsure -6.026e-01 3.719e-01 -1.621 0.125

Age 3.605e-05 5.844e-02 0.001 1.000

ObjectPipe:Persistence -5.450e-04 5.707e-04 -0.955 0.354

------------------------------------------------------------------

Sigma link function: log

Sigma Coefficients:

Estimate Std. Error t value Pr(>|t|)

(Intercept) -0.7749 0.1474 -5.255 7.85e-05 ***

---

Signif. codes: 0 ‘***’ 0.001 ‘**’ 0.01 ‘*’ 0.05 ‘.’ 0.1 ‘ ’ 1

------------------------------------------------------------------

No. of observations in the fit: 23

Degrees of Freedom for the fit: 7

Residual Deg. of Freedom: 16

at cycle: 2

Global Deviance: 32.36346

AIC: 46.36346

SBC: 54.31192

******************************************************************

###### dropterm(Latency.Wolves.LOGNO.2, test = "Chisq")

Single term deletions for mu

Model: Contact.Latency ~ Object * Persistence + Approach.Posture + Age

Df AIC LRT Pr(Chi)

<none> 46.363

Approach.Posture 1 46.850 2.48703 0.1148

Age 1 44.363 0.00000 0.9995

Object:Persistence 1 45.258 0.89422 0.3443

###### summary(Latency.Wolves.LOGNO.3)

******************************************************************

Family: c("LOGNO", "Log Normal")

Call: gamlss(formula = Contact.Latency ~ Persistence + Object + Approach.Posture +

Age, family = "LOGNO", data = na.omit(wolves),

control = con, random = ~1 | Individual)

Fitting method: RS()

------------------------------------------------------------------

Mu link function: identity

Mu Coefficients:

Estimate Std. Error t value Pr(>|t|)

(Intercept) 0.5406153 0.4152015 1.302 0.21026

Persistence -0.0009686 0.0002833 -3.418 0.00327 **

ObjectPipe -0.2024413 0.2102612 -0.963 0.34914

Approach.PostureUnsure -0.6470474 0.3761826 -1.720 0.10358

Age -0.0025617 0.0595226 -0.043 0.96617

---

Signif. codes: 0 ‘***’ 0.001 ‘**’ 0.01 ‘*’ 0.05 ‘.’ 0.1 ‘ ’ 1

------------------------------------------------------------------

Sigma link function: log

Sigma Coefficients:

Estimate Std. Error t value Pr(>|t|)

(Intercept) -0.7554 0.1474 -5.124 8.47e-05 ***

------------------------------------------------------------------

No. of observations in the fit: 23

Degrees of Freedom for the fit: 6

Residual Deg. of Freedom: 17

at cycle: 2

Global Deviance: 33.25768

AIC: 45.25768

SBC: 52.07065

******************************************************************

##### Gamma

###### summary(Latency.Wolves.GA)

******************************************************************

Family: c("GA", "Gamma")

Call: gamlss(formula = Contact.Latency ~ Object * Approach.Posture +

Object * Persistence + Age, family = "GA", data = na.omit(wolves),

control = con, random = ~1 | Individual)

Fitting method: RS()

------------------------------------------------------------------

Mu link function: identity

Mu Coefficients:

Estimate Std. Error t value Pr(>|t|)

(Intercept) 0.4483751 0.4331479 1.035 0.3160

ObjectPipe 0.0058086 0.2723396 0.021 0.9832

Approach.PostureUnsure -0.6013323 0.3701945 -1.624 0.1238

Persistence -0.0006806 0.0003790 -1.796 0.0914 .

Age 0.0041454 0.0600371 0.069 0.9458

ObjectPipe:Persistence -0.0003632 0.0004724 -0.769 0.4532

---

Signif. codes: 0 ‘***’ 0.001 ‘**’ 0.01 ‘*’ 0.05 ‘.’ 0.1 ‘ ’ 1

------------------------------------------------------------------

Sigma link function: log

Sigma Coefficients:

Estimate Std. Error t value Pr(>|t|)

(Intercept) -0.8048 0.1484 -5.425 5.62e-05 ***

------------------------------------------------------------------

No. of observations in the fit: 23

Degrees of Freedom for the fit: 7

Residual Deg. of Freedom: 16

at cycle: 2

Global Deviance: 32.51758

AIC: 46.51758

SBC: 54.46604

******************************************************************

###### dropterm(Latency.Wolves.GA, test = "Chisq")

Single term deletions for mu

Model: Contact.Latency ~ Object * Approach.Posture + Object * Persistence + Age

Df AIC LRT Pr(Chi)

<none> 46.518

Age 1 44.523 0.0051 0.9431

Object:Approach.Posture 0 46.518 0.0000

Object:Persistence 1 44.950 0.4321 0.5110

###### summary(Latency.Wolves.GA.2)

******************************************************************

Family: c("GA", "Gamma")

Call: gamlss(formula = Contact.Latency ~ Object * Persistence + Approach.Posture +

Age, family = "GA", data = na.omit(wolves),

control = con, random = ~1 | Individual)

Fitting method: RS()

------------------------------------------------------------------

Mu link function: identity

Mu Coefficients:

Estimate Std. Error t value Pr(>|t|)

(Intercept) 0.4483751 0.4331479 1.035 0.3160

ObjectPipe 0.0058086 0.2723396 0.021 0.9832

Persistence -0.0006806 0.0003790 -1.796 0.0914 .

Approach.PostureUnsure -0.6013323 0.3701945 -1.624 0.1238

Age 0.0041454 0.0600371 0.069 0.9458

ObjectPipe:Persistence -0.0003632 0.0004724 -0.769 0.4532

---

Signif. codes: 0 ‘***’ 0.001 ‘**’ 0.01 ‘*’ 0.05 ‘.’ 0.1 ‘ ’ 1

------------------------------------------------------------------

Sigma link function: log

Sigma Coefficients:

Estimate Std. Error t value Pr(>|t|)

(Intercept) -0.8048 0.1484 -5.425 5.62e-05 ***

------------------------------------------------------------------

No. of observations in the fit: 23

Degrees of Freedom for the fit: 7

Residual Deg. of Freedom: 16

at cycle: 2

Global Deviance: 32.51758

AIC: 46.51758

SBC: 54.46604

******************************************************************

###### dropterm(Latency.Wolves.GA.2, test = "Chisq")

Single term deletions for mu

Model: Contact.Latency ~ Object * Persistence + Approach.Posture + Age

Df AIC LRT Pr(Chi)

<none> 46.518

Approach.Posture 1 46.790 2.2726 0.1317

Age 1 44.523 0.0051 0.9431

Object:Persistence 1 44.950 0.4321 0.5110

###### summary(Latency.Wolves.GA.3)

******************************************************************

Family: c("GA", "Gamma")

Call: gamlss(formula = Contact.Latency ~ Persistence + Object + Approach.Posture +

Age, family = "GA", data = na.omit(wolves),

control = con, random = ~1 | Individual)

Fitting method: RS()

------------------------------------------------------------------

Mu link function: identity

Mu Coefficients:

Estimate Std. Error t value Pr(>|t|)

(Intercept) 0.5869804 0.3865818 1.518 0.14729

Persistence -0.0008988 0.0002418 -3.717 0.00171 **

ObjectPipe -0.1210280 0.2061554 -0.587 0.56487

Approach.PostureUnsure -0.6493395 0.3675023 -1.767 0.09519 .

Age -0.0033821 0.0592149 -0.057 0.95512

---

Signif. codes: 0 ‘***’ 0.001 ‘**’ 0.01 ‘*’ 0.05 ‘.’ 0.1 ‘ ’ 1

------------------------------------------------------------------

Sigma link function: log

Sigma Coefficients:

Estimate Std. Error t value Pr(>|t|)

(Intercept) -0.7960 0.1481 -5.374 5.05e-05 ***

------------------------------------------------------------------

No. of observations in the fit: 23

Degrees of Freedom for the fit: 6

Residual Deg. of Freedom: 17

at cycle: 2

Global Deviance: 32.94968

AIC: 44.94968

SBC: 51.76264

******************************************************************

##### Weibull

###### summary(Latency.Wolves.WEI)

******************************************************************

Family: c("WEI", "Weibull")

Call: gamlss(formula = Contact.Latency ~ Object * Approach.Posture +

Object * Persistence + Age, family = "WEI", data = na.omit(wolves),

control = con, random = ~1 | Individual)

Fitting method: RS()

------------------------------------------------------------------

Mu link function: identity

Mu Coefficients:

Estimate Std. Error t value Pr(>|t|)

(Intercept) 0.5621230 0.4213164 1.334 0.2008

ObjectPipe 0.0774597 0.2578471 0.300 0.7677

Approach.PostureUnsure -0.5876850 0.3490992 -1.683 0.1117

Persistence -0.0007229 0.0004001 -1.807 0.0896 .

Age -0.0005306 0.0611897 -0.009 0.9932

ObjectPipe:Persistence -0.0002476 0.0004970 -0.498 0.6252

---

Signif. codes: 0 ‘***’ 0.001 ‘**’ 0.01 ‘*’ 0.05 ‘.’ 0.1 ‘ ’ 1

------------------------------------------------------------------

Sigma link function: log

Sigma Coefficients:

Estimate Std. Error t value Pr(>|t|)

(Intercept) 0.9101 0.1662 5.476 5.08e-05 ***

------------------------------------------------------------------

No. of observations in the fit: 23

Degrees of Freedom for the fit: 7

Residual Deg. of Freedom: 16

at cycle: 4

Global Deviance: 32.97858

AIC: 46.97858

SBC: 54.92704

******************************************************************

###### dropterm(Latency.Wolves.WEI, test = "Chisq")

Single term deletions for mu

Model: Contact.Latency ~ Object * Approach.Posture + Object * Persistence + Age

Df AIC LRT Pr(Chi)

<none> 46.979

Age 1 44.979 0.000081 0.9928

Object:Approach.Posture 0 46.979 0.000000

Object:Persistence 1 45.221 0.241950 0.6228

###### summary(Latency.Wolves.WEI.2)

******************************************************************

Family: c("WEI", "Weibull")

Call: gamlss(formula = Contact.Latency ~ Object * Persistence + Approach.Posture +

Age, family = "WEI", data = na.omit(wolves),

control = con, random = ~1 | Individual)

Fitting method: RS()

------------------------------------------------------------------

Mu link function: identity

Mu Coefficients:

Estimate Std. Error t value Pr(>|t|)

(Intercept) 0.5621230 0.3274350 1.717 0.1053

ObjectPipe 0.0774597 0.2576337 0.301 0.7675

Persistence -0.0007229 0.0003989 -1.812 0.0887 .

Approach.PostureUnsure -0.5876850 0.3457811 -1.700 0.1086

Age -0.0005306 0.0402149 -0.013 0.9896

ObjectPipe:Persistence -0.0002476 0.0004906 -0.505 0.6207

---

Signif. codes: 0 ‘***’ 0.001 ‘**’ 0.01 ‘*’ 0.05 ‘.’ 0.1 ‘ ’ 1

------------------------------------------------------------------

Sigma link function: log

Sigma Coefficients:

Estimate Std. Error t value Pr(>|t|)

(Intercept) 0.9101 0.1662 5.477 5.07e-05 ***

------------------------------------------------------------------

No. of observations in the fit: 23

Degrees of Freedom for the fit: 7

Residual Deg. of Freedom: 16

at cycle: 4

Global Deviance: 32.97858

AIC: 46.97858

SBC: 54.92704

******************************************************************

###### dropterm(Latency.Wolves.WEI.2, test = "Chisq")

Single term deletions for mu

Model: Contact.Latency ~ Object * Persistence + Approach.Posture + Age

Df AIC LRT Pr(Chi)

<none> 46.979

Approach.Posture 1 47.177 2.19812 0.1382

Age 1 44.979 0.00008 0.9928

Object:Persistence 1 45.221 0.24195 0.6228

###### summary(Latency.Wolves.WEI.3)

******************************************************************

Family: c("WEI", "Weibull")

Call: gamlss(formula = Contact.Latency ~ Persistence + Object + Approach.Posture +

Age, family = "WEI", data = na.omit(wolves),

control = con, random = ~1 | Individual)

Fitting method: RS()

------------------------------------------------------------------

Mu link function: identity

Mu Coefficients:

Estimate Std. Error t value Pr(>|t|)

(Intercept) 0.6782045 0.3522054 1.926 0.07105 .

Persistence -0.0008737 0.0002496 -3.500 0.00274 **

ObjectPipe -0.0122778 0.1901729 -0.065 0.94928

Approach.PostureUnsure -0.6379362 0.3354641 -1.902 0.07430 .

Age -0.0084118 0.0562303 -0.150 0.88284

---

Signif. codes: 0 ‘***’ 0.001 ‘**’ 0.01 ‘*’ 0.05 ‘.’ 0.1 ‘ ’ 1

------------------------------------------------------------------

Sigma link function: log

Sigma Coefficients:

Estimate Std. Error t value Pr(>|t|)

(Intercept) 0.9057 0.1658 5.464 4.2e-05 ***

------------------------------------------------------------------

No. of observations in the fit: 23

Degrees of Freedom for the fit: 6

Residual Deg. of Freedom: 17

at cycle: 4

Global Deviance: 33.22053

AIC: 45.22053

SBC: 52.0335

******************************************************************

##### Generalised Inverse Gaussian

###### summary(Latency.Wolves.GIG)

******************************************************************

Family: c("GIG", "Generalised Inverse Gaussian")

Call: gamlss(formula = Contact.Latency ~ Object * Approach.Posture +

Object * Persistence + Age, family = "GIG", data = na.omit(wolves),

control = con, random = ~1 | Individual)

Fitting method: RS()

------------------------------------------------------------------

Mu link function: identity

Mu Coefficients:

Estimate Std. Error t value Pr(>|t|)

(Intercept) 0.4965731 0.4308583 1.153 0.265

ObjectPipe -0.0069290 0.2809814 -0.025 0.981

Approach.PostureUnsure -0.5969354 0.3679807 -1.622 0.123

Persistence -0.0006269 0.0004426 -1.417 0.175

Age -0.0004834 0.0578306 -0.008 0.993

ObjectPipe:Persistence -0.0005623 0.0005648 -0.996 0.333

------------------------------------------------------------------

Sigma link function: log

Sigma Coefficients:

Estimate Std. Error t value Pr(>|t|)

(Intercept) -0.729 0.188 -3.878 0.000811 ***

---

Signif. codes: 0 ‘***’ 0.001 ‘**’ 0.01 ‘*’ 0.05 ‘.’ 0.1 ‘ ’ 1

------------------------------------------------------------------

Nu link function: identity

Nu Coefficients:

Estimate Std. Error t value Pr(>|t|)

(Intercept) -0.4295 10.5923 -0.041 0.968

------------------------------------------------------------------

No. of observations in the fit: 23

Degrees of Freedom for the fit: 8

Residual Deg. of Freedom: 15

at cycle: 4

Global Deviance: 32.04921

AIC: 48.04921

SBC: 57.13316

******************************************************************

###### dropterm(Latency.Wolves.GIG, test = "Chisq")

Single term deletions for mu

Model: Contact.Latency ~ Object * Approach.Posture + Object * Persistence + Age

Df AIC LRT Pr(Chi)

<none> 48.049

Age 1 46.049 0.00002 0.9962

Object:Approach.Posture 0 48.049 0.00000

Object:Persistence 1 46.810 0.76050 0.3832

###### summary(Latency.Wolves.GIG.2)

******************************************************************

Family: c("GIG", "Generalised Inverse Gaussian")

Call: gamlss(formula = Contact.Latency ~ Object * Persistence + Approach.Posture +

Age, family = "GIG", data = na.omit(wolves),

control = con, random = ~1 | Individual)

Fitting method: RS()

------------------------------------------------------------------

Mu link function: identity

Mu Coefficients:

Estimate Std. Error t value Pr(>|t|)

(Intercept) 0.4965731 0.4308583 1.153 0.265

ObjectPipe -0.0069290 0.2809814 -0.025 0.981

Persistence -0.0006269 0.0004426 -1.417 0.175

Approach.PostureUnsure -0.5969354 0.3679807 -1.622 0.123

Age -0.0004834 0.0578306 -0.008 0.993

ObjectPipe:Persistence -0.0005623 0.0005648 -0.996 0.333

------------------------------------------------------------------

Sigma link function: log

Sigma Coefficients:

Estimate Std. Error t value Pr(>|t|)

(Intercept) -0.729 0.188 -3.878 0.000811 ***

---

Signif. codes: 0 ‘***’ 0.001 ‘**’ 0.01 ‘*’ 0.05 ‘.’ 0.1 ‘ ’ 1

------------------------------------------------------------------

Nu link function: identity

Nu Coefficients:

Estimate Std. Error t value Pr(>|t|)

(Intercept) -0.4295 10.5923 -0.041 0.968

------------------------------------------------------------------

No. of observations in the fit: 23

Degrees of Freedom for the fit: 8

Residual Deg. of Freedom: 15

at cycle: 4

Global Deviance: 32.04921

AIC: 48.04921

SBC: 57.13316

******************************************************************

###### dropterm(Latency.Wolves.GIG.2, test = "Chisq")

Single term deletions for mu

Model: Contact.Latency ~ Object * Persistence + Approach.Posture + Age

Df AIC LRT Pr(Chi)

<none> 48.049

Approach.Posture 1 48.475 2.42612 0.1193

Age 1 46.049 0.00002 0.9962

Object:Persistence 1 46.810 0.76050 0.3832

###### summary(Latency.Wolves.GIG.3)

******************************************************************

Family: c("GIG", "Generalised Inverse Gaussian")

Call: gamlss(formula = Contact.Latency ~ Persistence + Object + Approach.Posture +

Age, family = "GIG", data = na.omit(wolves),

control = con, random = ~1 | Individual)

Fitting method: RS()

------------------------------------------------------------------

Mu link function: identity

Mu Coefficients:

Estimate Std. Error t value Pr(>|t|)

(Intercept) 0.6065597 0.6188065 0.980 0.34157

Persistence -0.0009467 0.0002995 -3.161 0.00606 **

ObjectPipe -0.1609172 0.2296859 -0.701 0.49362

Approach.PostureUnsure -0.6530659 0.3897705 -1.676 0.11326

Age -0.0007817 0.0995881 -0.008 0.99383

---

Signif. codes: 0 ‘***’ 0.001 ‘**’ 0.01 ‘*’ 0.05 ‘.’ 0.1 ‘ ’ 1

------------------------------------------------------------------

Sigma link function: log

Sigma Coefficients:

Estimate Std. Error t value Pr(>|t|)

(Intercept) -0.6378 0.4539 -1.405 0.179

------------------------------------------------------------------

Nu link function: identity

Nu Coefficients:

Estimate Std. Error t value Pr(>|t|)

(Intercept) 2.542 6.060 0.419 0.68

------------------------------------------------------------------

No. of observations in the fit: 23

Degrees of Freedom for the fit: 7

Residual Deg. of Freedom: 16

at cycle: 10

Global Deviance: 32.80971

AIC: 46.80971

SBC: 54.75817

******************************************************************

##### Model AIC Values & Plots

|  | **df** | **AIC** |
| --- | --- | --- |
| **LOGNO Models** |  |  |
| Latency.Wolves.LOGNO.3 | 6 | 45.2576836 |
| Latency.Wolves.LOGNO.2 | 7 | 46.3634629 |
| Latency.Wolves.LOGNO | 7 | 46.3634629 |
| **GA Models** |  |  |
| Latency.Wolves.GA.3 | 6 | 44.94967512 |
| Latency.Wolves.GA.2 | 7 | 46.51757949 |
| Latency.Wolves.GA | 7 | 46.51757949 |
| **WEI Models** |  |  |
| Latency.Wolves.WEI.3 | 6 | 45.22053018 |
| Latency.Wolves.WEI | 7 | 46.97858022 |
| Latency.Wolves.WEI.2 | 7 | 46.97858022 |
| **GIG Models** |  |  |
| Latency.Wolves.GIG.3 | 7 | 46.80970824 |
| Latency.Wolves.GIG.2 | 8 | 48.04920909 |
| Latency.Wolves.GIG | 8 | 48.04920909 |

#### Plots


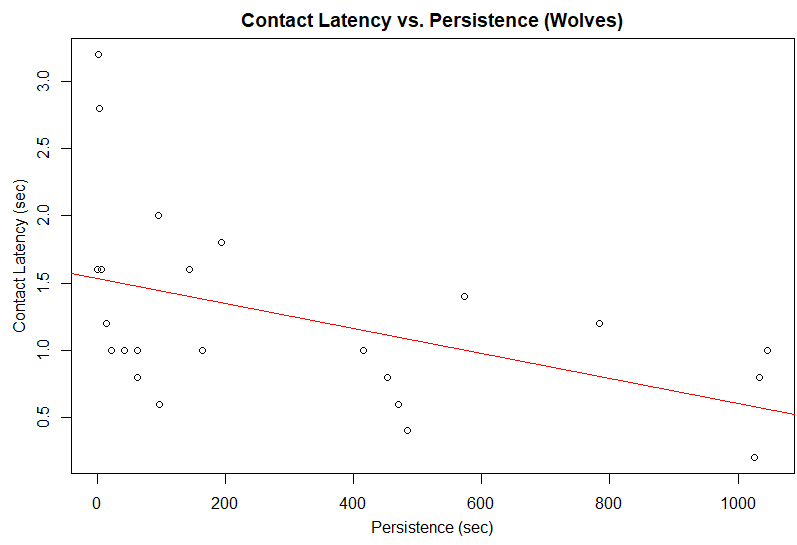


## Motor Diversity Models

### Dogs

#### Response Variable Distribution

###### Dogs.Diversity.Distribution$fit

| Distribution | AIC |
| --- | --- |
| GEOM | 131.840356 |
| GEOMo | 131.840356 |
| DPO | 131.926183 |
| NBI | 132.9925 |
| NBII | 132.9925 |
| GPO | 133.39636 |
| ZAPIG | 133.454585 |
| ZIPIG | 133.454585 |
| ZINBI | 133.50528 |
| ZANBI | 133.50528 |
| WARING | 133.840359 |
| ZIP | 133.929318 |
| ZAP | 133.929318 |
| PIG | 133.929318 |
| NBF | 134.143332 |

##### Density plots of distributions used in final models


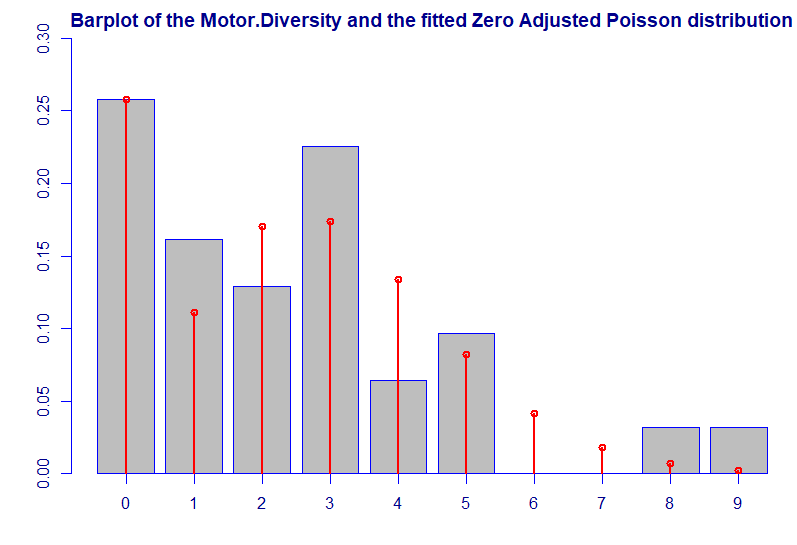

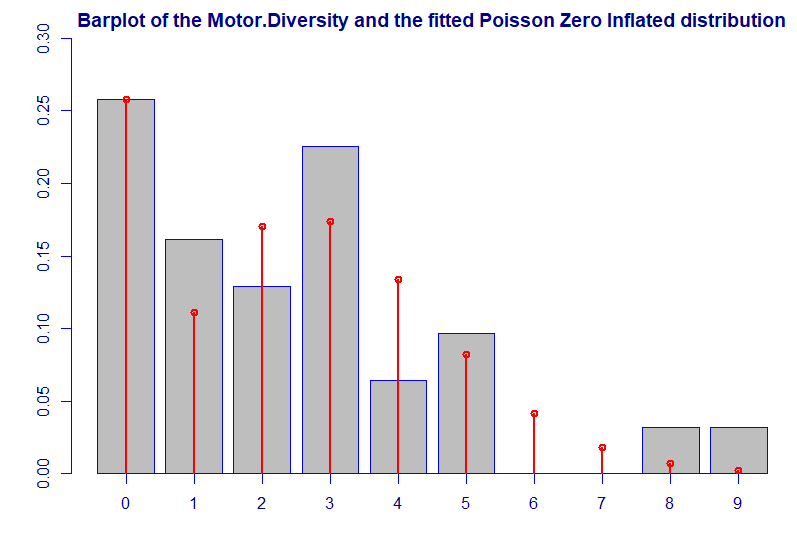


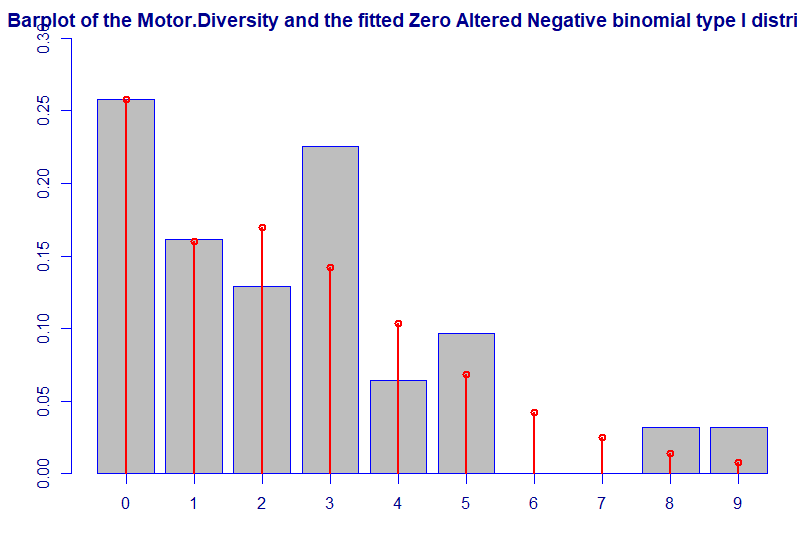

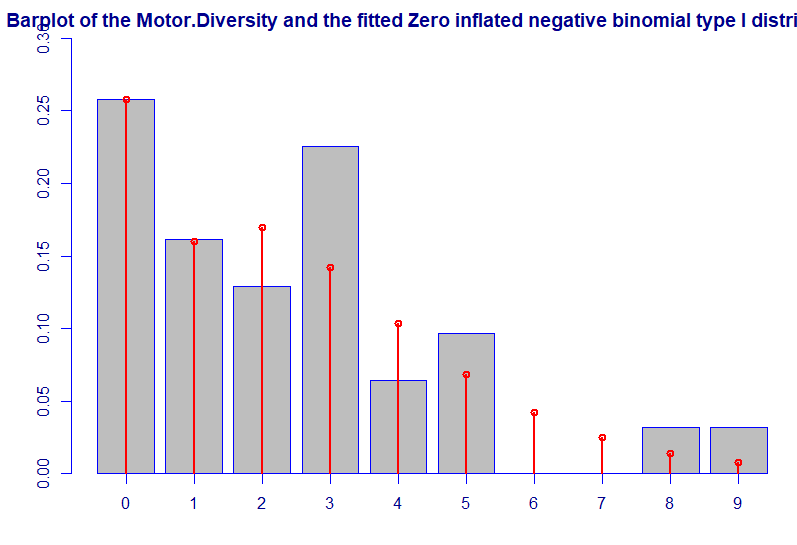


#### Model Distribution Selection

Diversity.Dogs.DISTRIBUTION <- gamlss(Motor.Diversity ~ Object*Persistence + Age,

random = ~1|Individual,

family = "DISTRIBUTION", data = dogs)

| Model | df | AIC |
| --- | --- | --- |
| Diversity.Dogs.ZAP | 6 | 117.2878 |
| Diversity.Dogs.ZIP | 6 | 118.941638 |
| Diversity.Dogs.ZANBI | 7 | 119.287843 |
| Diversity.Dogs.ZINBI | 7 | 120.941578 |
| Diversity.Dogs.ZIPIG | 7 | 121.123295 |
| Diversity.Dogs.DPO | 6 | 122.587648 |
| Diversity.Dogs.ZAPIG | 7 | 123.594596 |
| Diversity.Dogs.NBI | 6 | 123.977027 |
| Diversity.Dogs.PIG | 6 | 123.986116 |
| Diversity.Dogs.GPO | 6 | 123.989319 |
| Diversity.Dogs.NBF | 7 | 125.232948 |
| Diversity.Dogs.GEOM | 5 | 130.605775 |

##### Model Plots

###### plot(Diversity.Dogs.ZAP) & wp(Diversity.Dogs.ZAP)


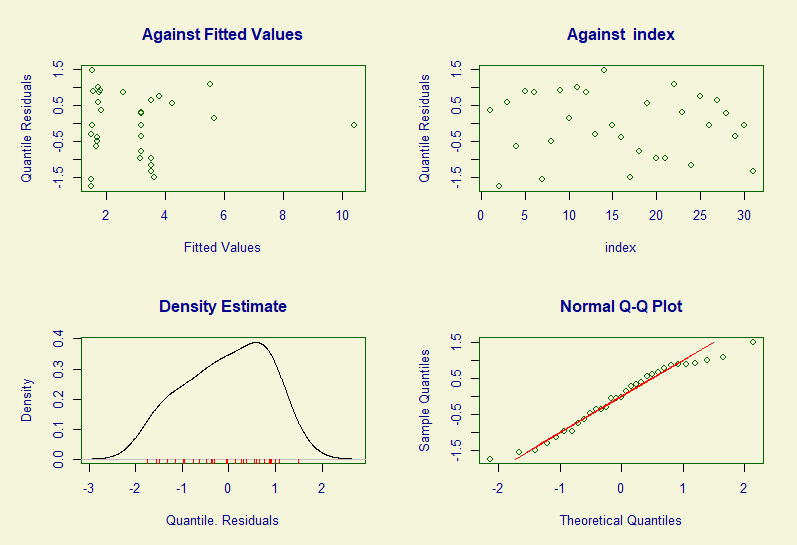

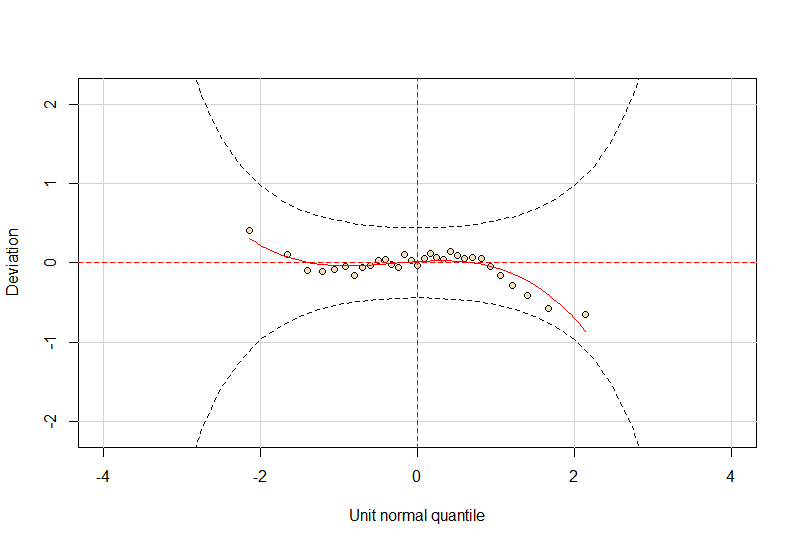


###### plot(Diversity.Dogs.ZIP) & wp(Diversity.Dogs.ZIP)


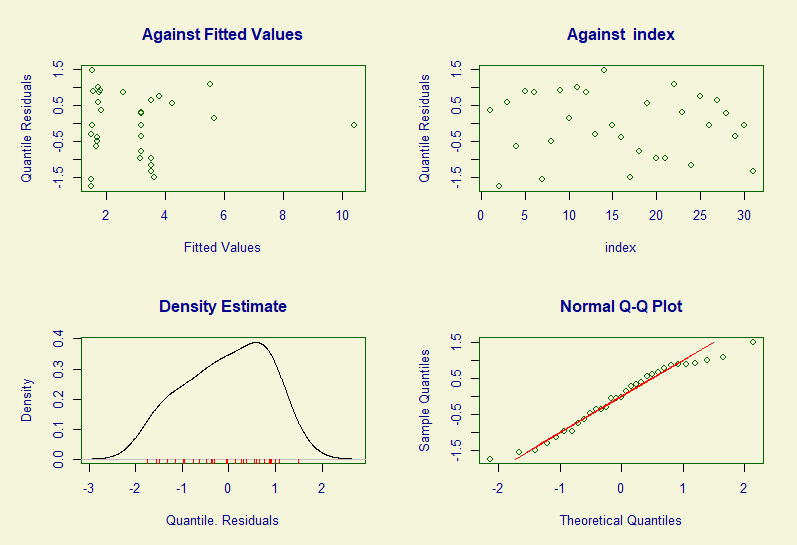

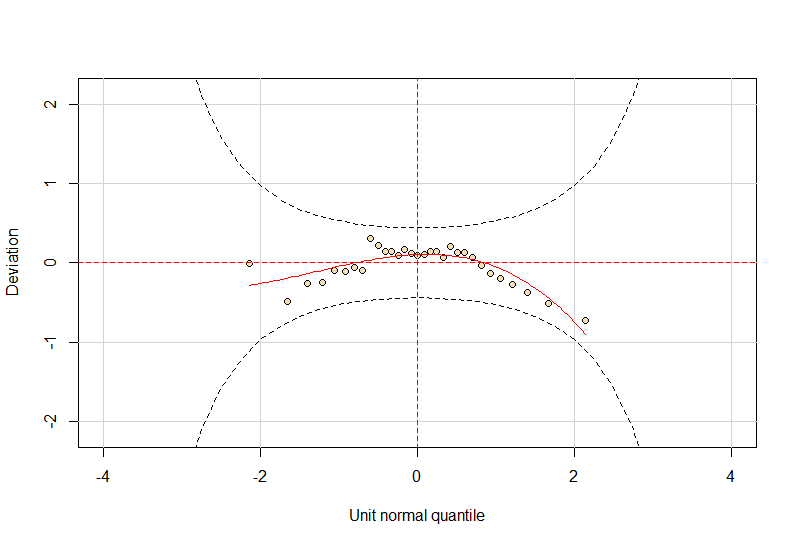


###### plot(Diversity.Dogs.ZANBI) & wp(Diversity.Dogs.ZANBI)


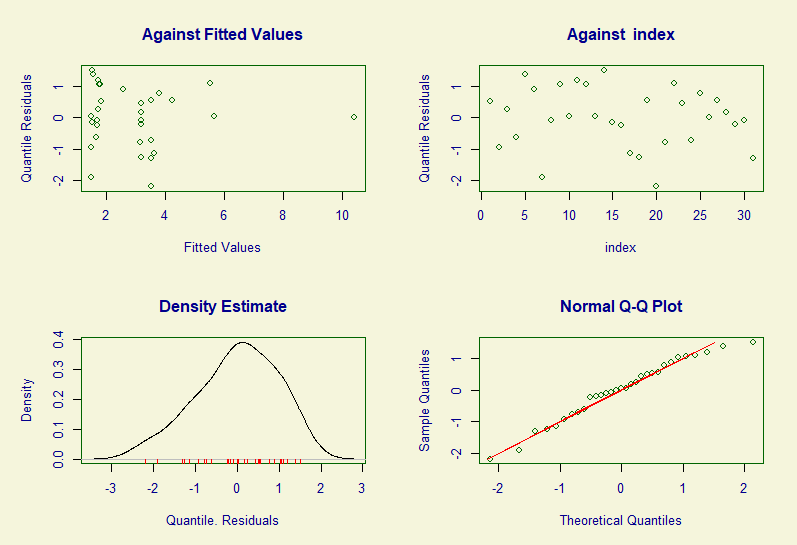

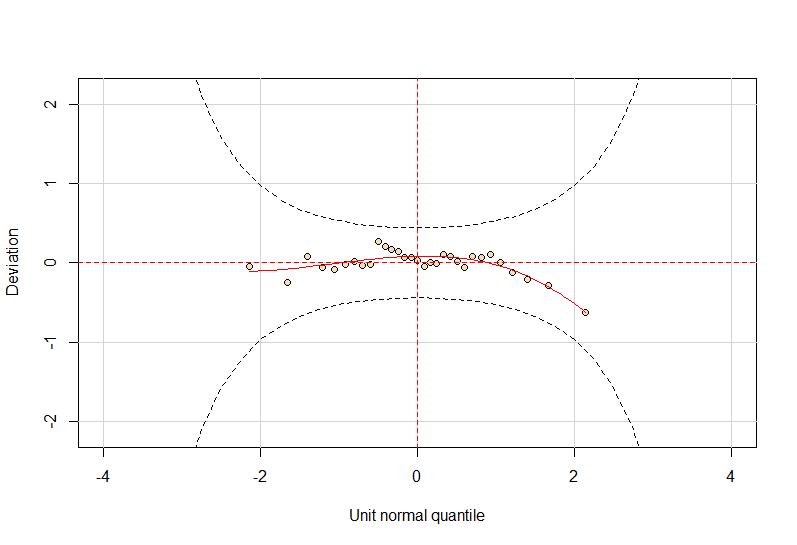


###### plot(Diversity.Dogs.ZINBI) & wp(Diversity.Dogs.ZINBI)


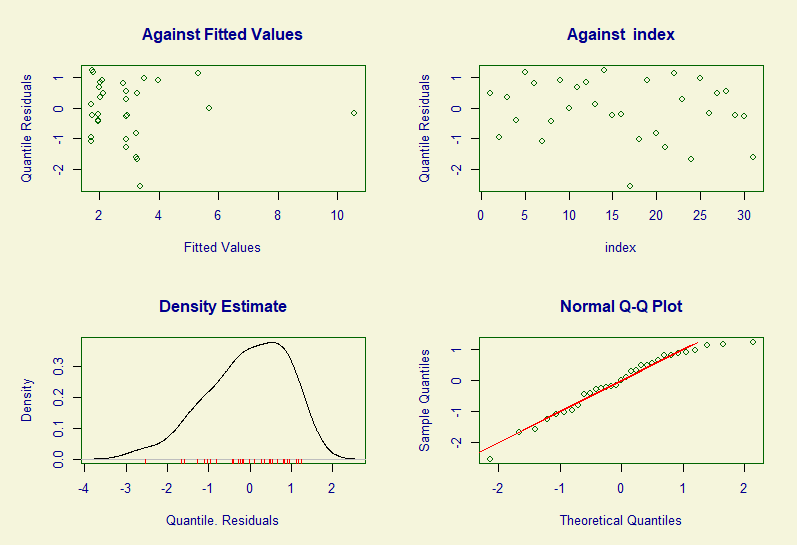

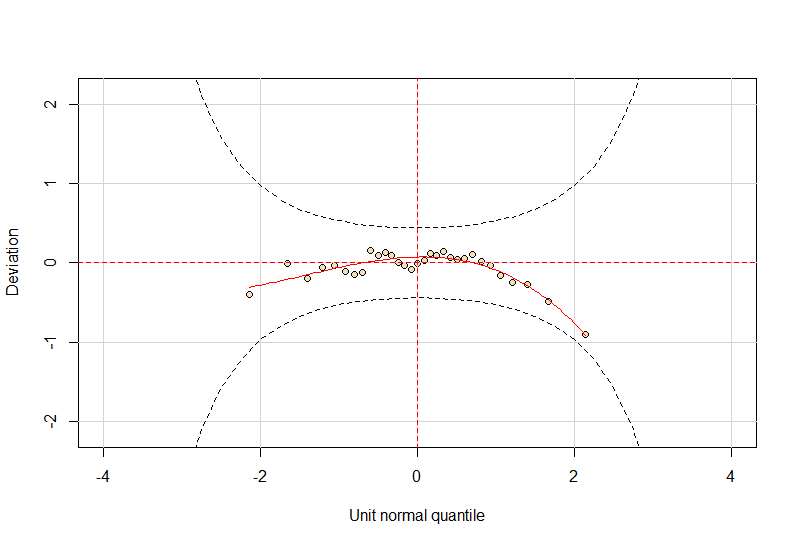


###### plot(Diversity.Dogs.ZIPIG) & wp(Diversity.Dogs.ZIPIG)


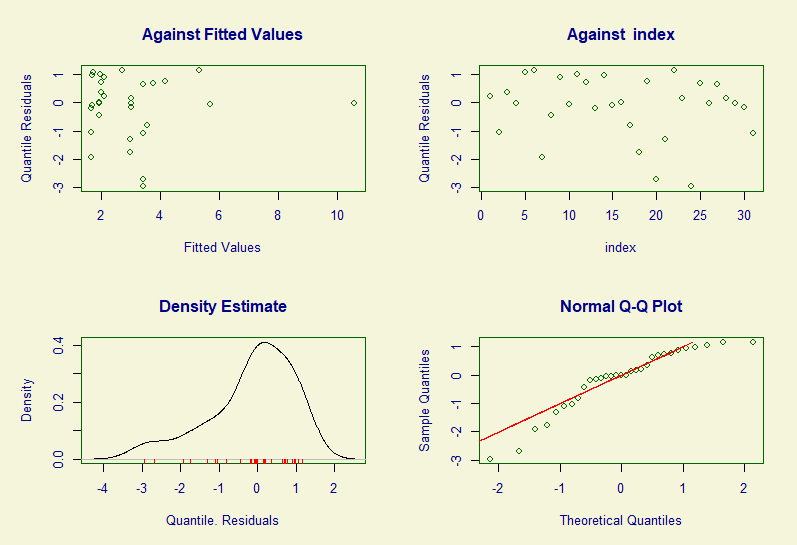

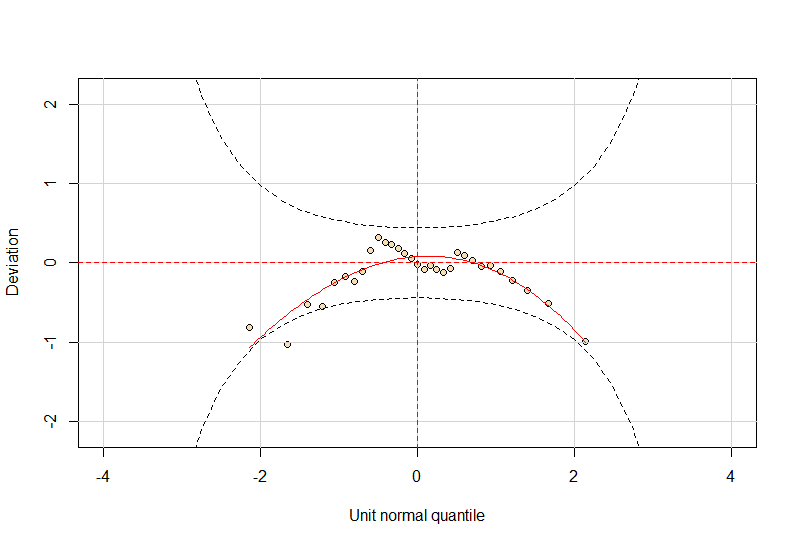


###### plot(Diversity.Dogs.DPO) & wp(Diversity.Dogs.DPO)


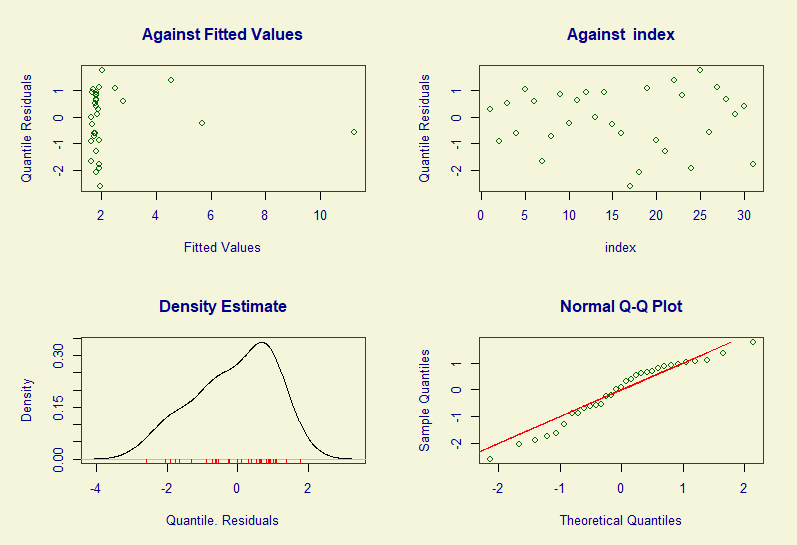

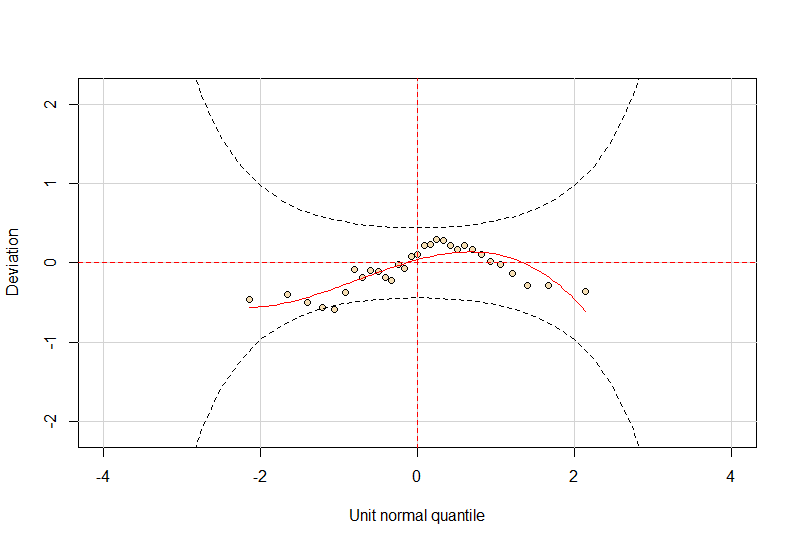


###### plot(Diversity.Dogs.ZAPIG) & wp(Diversity.Dogs.ZAPIG)


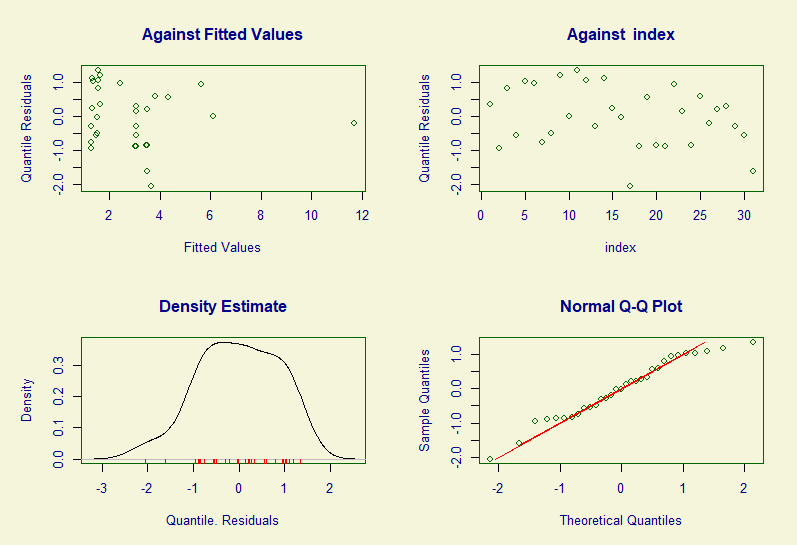

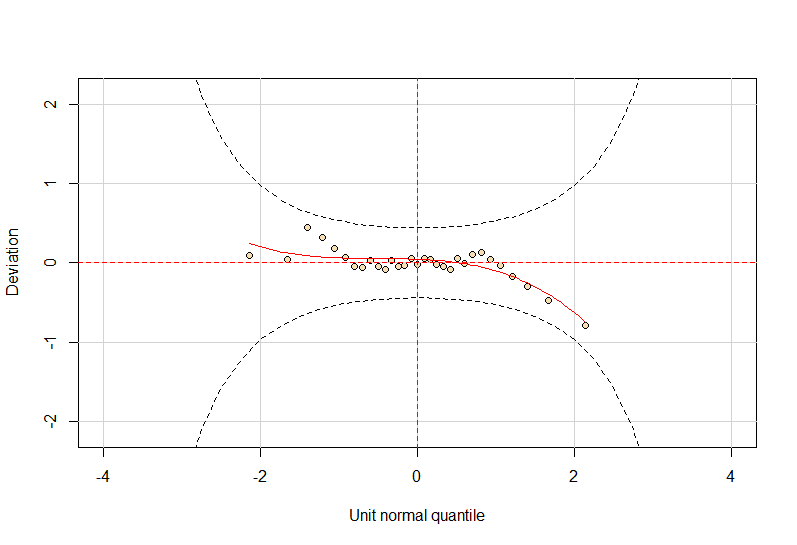


###### plot(Diversity.Dogs.NBI) & wp(Diversity.Dogs.NBI)


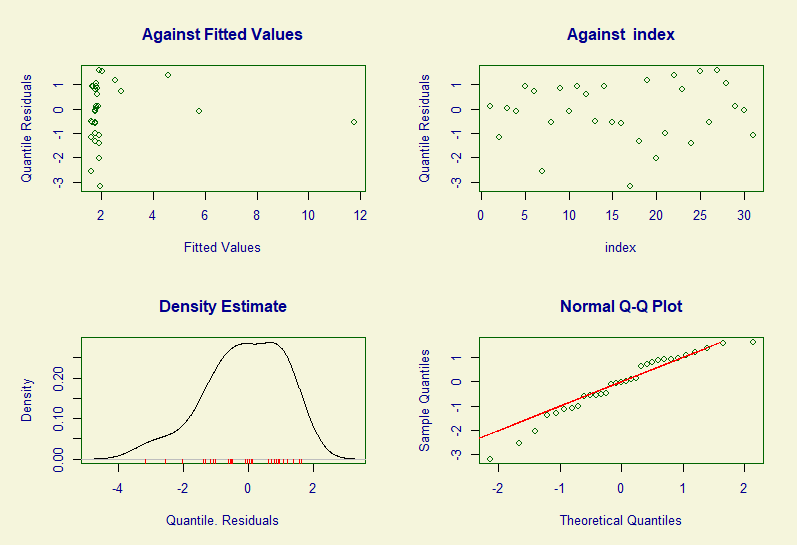

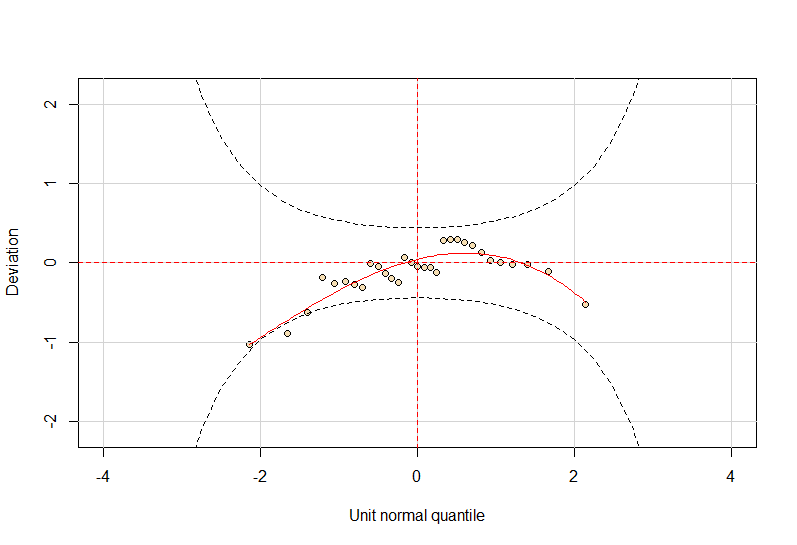


###### plot(Diversity.Dogs.PIG) & wp(Diversity.Dogs.PIG)


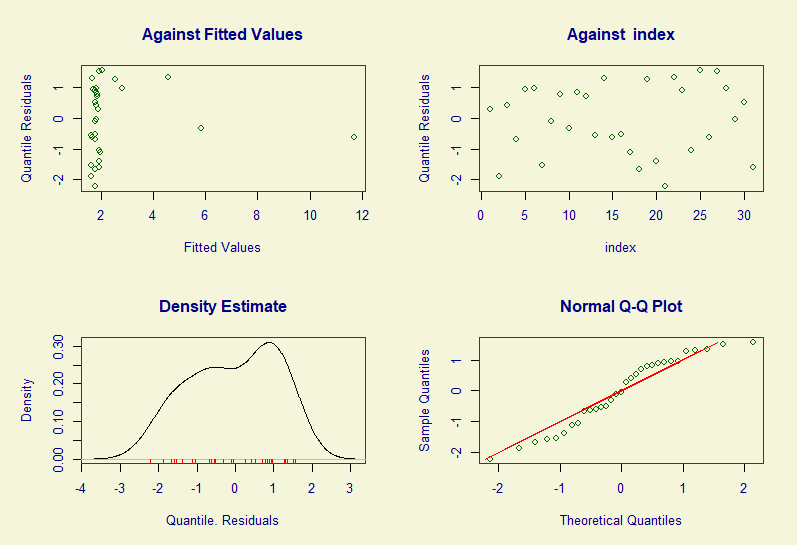

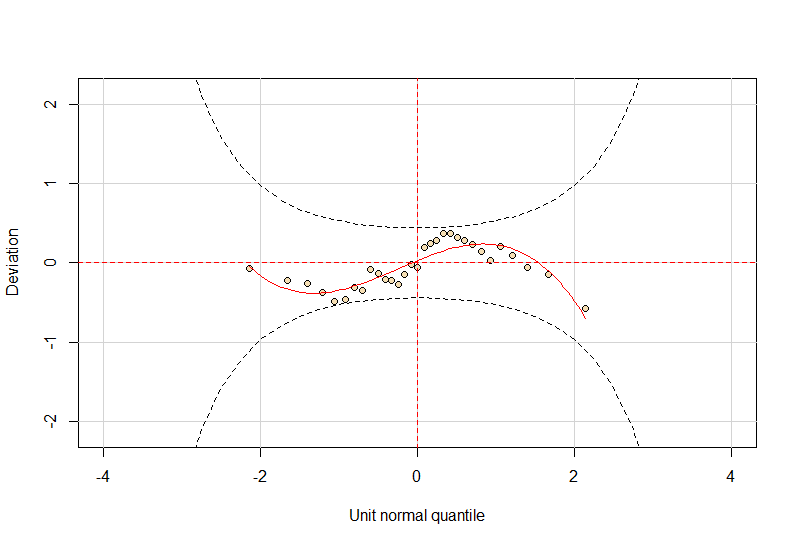


###### plot(Diversity.Dogs.GPO) & wp(Diversity.Dogs.GPO)


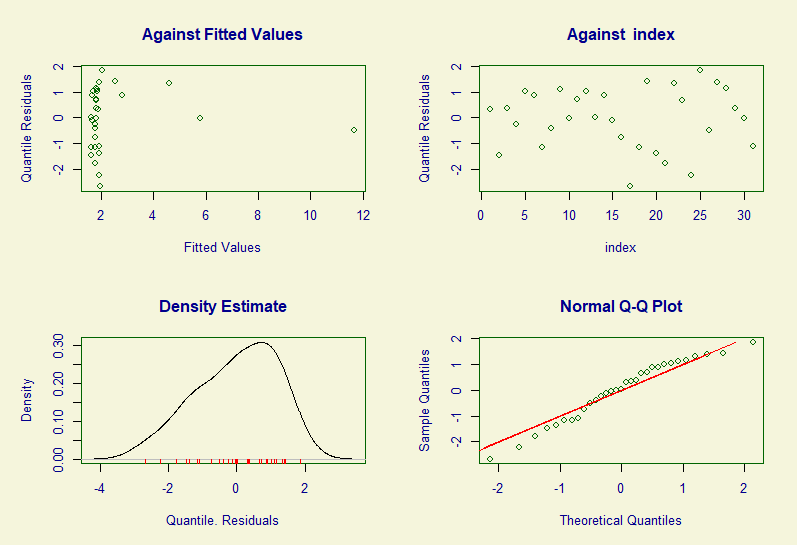

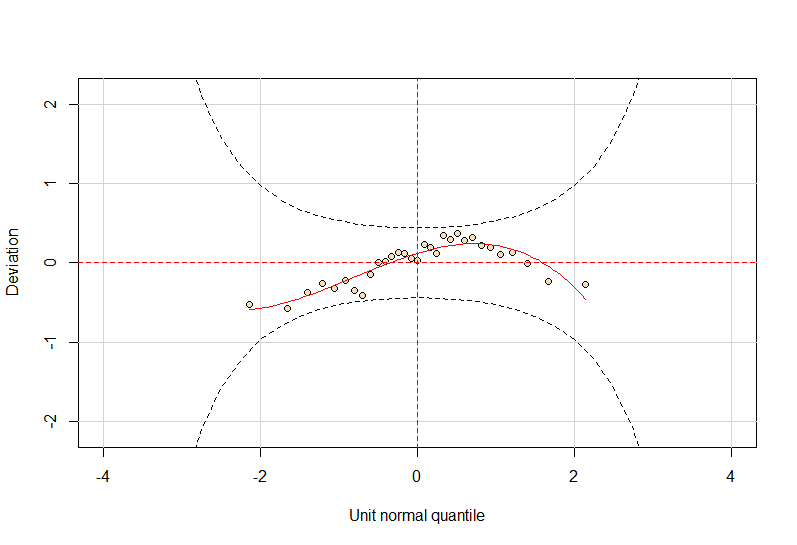


###### plot(Diversity.Dogs.NBF) & wp(Diversity.Dogs.NBF)


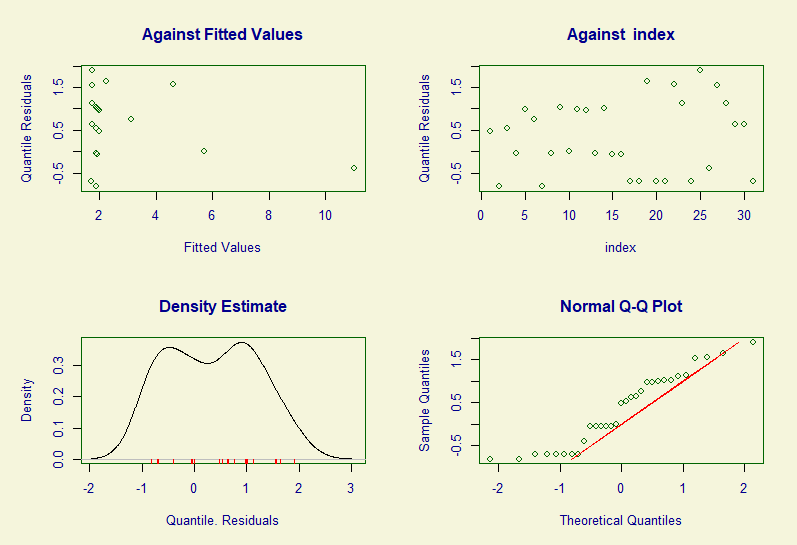

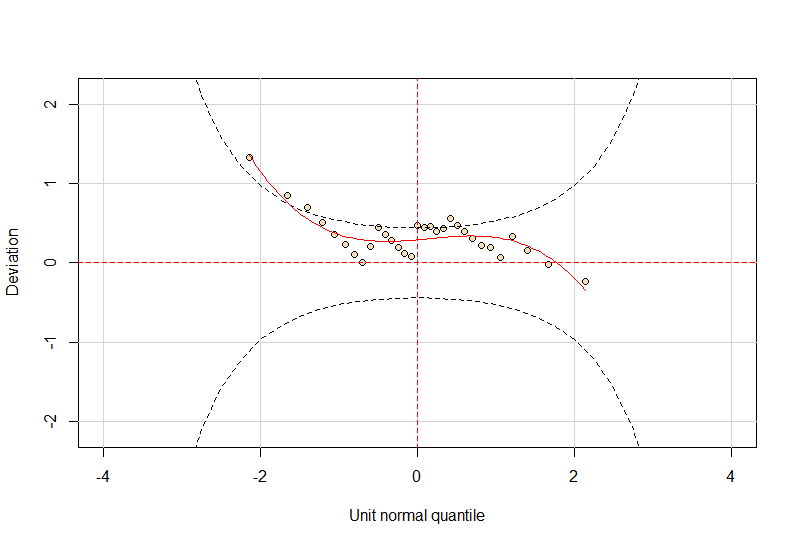


###### plot(Diversity.Dogs.GEOM) & wp(Diversity.Dogs.GEOM)


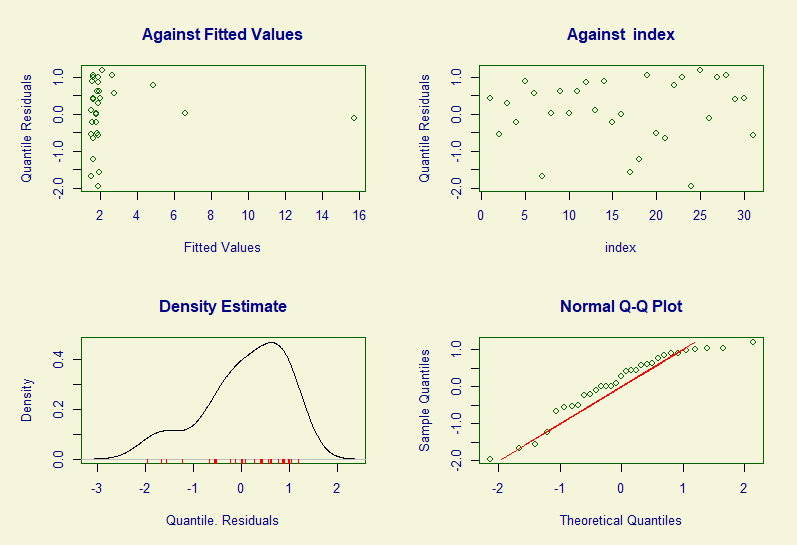

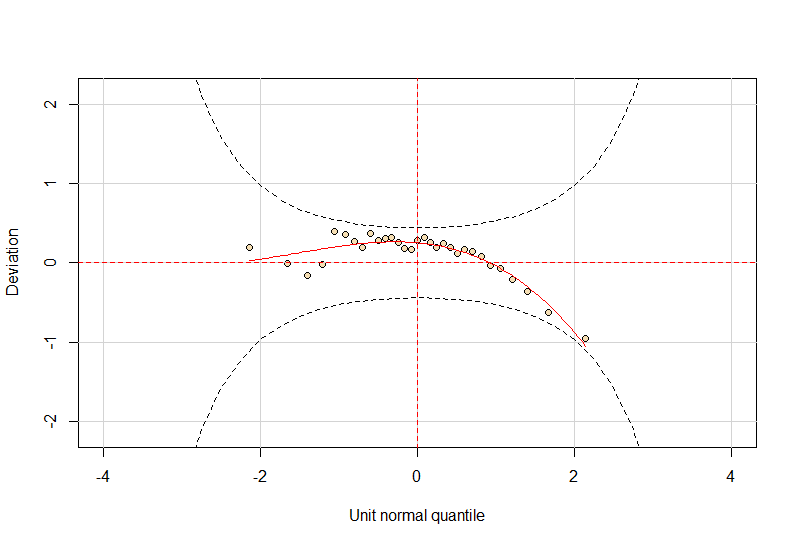


#### Model Reduction and Validation

##### Zero Adjusted Poisson

###### summary(Diversity.Dogs.ZAP)

******************************************************************

Family: c(“ZAP”, “Zero Adjusted Poisson”)

Call: gamlss(formula = Motor.Diversity ~ Object * Persistence + Age,

family = "ZAP", data = na.omit(dogs), control = con, random = ~1 | Individual)

Fitting method: RS()

------------------------------------------------------------------

Mu link function: log

Mu Coefficients:

Estimate Std. Error t value Pr(>|t|)

(Intercept) 0.316935 0.443544 0.715 0.4815

ObjectPipe 0.748283 0.337201 2.219 0.0358 *

Persistence 0.003257 0.001465 2.223 0.0355 *

Age 0.037068 0.083626 0.443 0.6614

ObjectPipe:Persistence -0.002452 0.001470 -1.668 0.1077

---

Signif. codes: 0 ‘***’ 0.001 ‘**’ 0.01 ‘*’ 0.05 ‘.’ 0.1 ‘ ’ 1

------------------------------------------------------------------

Sigma link function: logit

Sigma Coefficients:

Estimate Std. Error t value Pr(>|t|)

(Intercept) -1.0561 0.4105 -2.573 0.0164 *

------------------------------------------------------------------

No. of observations in the fit: 31

Degrees of Freedom for the fit: 6

Residual Deg. of Freedom: 25

at cycle: 2

Global Deviance: 105.2878

AIC: 117.2878

SBC: 125.8917

******************************************************************

###### dropterm(Diversity.Dogs.ZAP, test = "Chisq")

Single term deletions for mu

Model: Motor.Diversity ~ Object * Persistence + Age

Df AIC LRT Pr(Chi)

<none> 117.29

Age 1 115.51 0.21849 0.64019

Object:Persistence 1 118.01 2.71956 0.09913 .

---

Signif. codes: 0 ‘***’ 0.001 ‘**’ 0.01 ‘*’ 0.05 ‘.’ 0.1 ‘ ’ 1

###### summary(Diversity.Dogs.ZAP.2)

******************************************************************

Family: c(“ZAP”, “Zero Adjusted Poisson”)

Call: gamlss(formula = Motor.Diversity ~ Object + Persistence + Age,

family = "ZAP", data = na.omit(dogs), control = con, random = ~1 | Individual)

Fitting method: RS()

------------------------------------------------------------------

Mu link function: log

Mu Coefficients:

Estimate Std. Error t value Pr(>|t|)

(Intercept) 0.4659100 0.4244754 1.098 0.282435

ObjectPipe 0.5208601 0.2992994 1.740 0.093638 .

Persistence 0.0008714 0.0002328 3.744 0.000908 ***

Age 0.0477883 0.0825917 0.579 0.567832

---

Signif. codes: 0 ‘***’ 0.001 ‘**’ 0.01 ‘*’ 0.05 ‘.’ 0.1 ‘ ’ 1

------------------------------------------------------------------

Sigma link function: logit

Sigma Coefficients:

Estimate Std. Error t value Pr(>|t|)

(Intercept) -1.0561 0.4105 -2.573 0.0161 *

------------------------------------------------------------------

No. of observations in the fit: 31

Degrees of Freedom for the fit: 5

Residual Deg. of Freedom: 26

at cycle: 2

Global Deviance: 108.0074

AIC: 118.0074

SBC: 125.1773

******************************************************************

##### Poisson Zero Inflated

###### summary(Diversity.Dogs.ZIP)

******************************************************************

Family: c(“ZIP”, “Poisson Zero Inflated”)

Call: gamlss(formula = Motor.Diversity ~ Object * Persistence + Age,

family = "ZIP", data = na.omit(dogs), control = con, random = ~1 | Individual)

Fitting method: RS()

------------------------------------------------------------------

Mu link function: log

Mu Coefficients:

Estimate Std. Error t value Pr(>|t|)

(Intercept) 0.450355 0.407922 1.104 0.2801

ObjectPipe 0.513878 0.324300 1.585 0.1256

Persistence 0.002867 0.001355 2.115 0.0446 *

Age 0.042100 0.080686 0.522 0.6064

ObjectPipe:Persistence -0.001990 0.001362 -1.461 0.1565

---

Signif. codes: 0 ‘***’ 0.001 ‘**’ 0.01 ‘*’ 0.05 ‘.’ 0.1 ‘ ’ 1

------------------------------------------------------------------

Sigma link function: logit

Sigma Coefficients:

Estimate Std. Error t value Pr(>|t|)

(Intercept) -1.3655 0.5367 -2.544 0.0175 *

------------------------------------------------------------------

No. of observations in the fit: 31

Degrees of Freedom for the fit: 6

Residual Deg. of Freedom: 25

at cycle: 4

Global Deviance: 106.9416

AIC: 118.9416

SBC: 127.5456

******************************************************************

###### dropterm(Diversity.Dogs.ZIP, test = "Chisq")

Single term deletions for mu

Model: Motor.Diversity ~ Object * Persistence + Age

Df AIC LRT Pr(Chi)

<none> 118.94

Age 1 117.22 0.27643 0.5991

Object:Persistence 1 119.00 2.06065 0.1511

###### summary(Diversity.Dogs.ZIP.2)

******************************************************************

Family: c(“ZIP”, “Poisson Zero Inflated”)

Call: gamlss(formula = Motor.Diversity ~ Object + Persistence + Age,

family = "ZIP", data = na.omit(dogs), control = con, random = ~1 | Individual)

Fitting method: RS()

------------------------------------------------------------------

Mu link function: log

Mu Coefficients:

Estimate Std. Error t value Pr(>|t|)

(Intercept) 0.5632811 0.4018451 1.402 0.172820

ObjectPipe 0.3443769 0.3051545 1.129 0.269401

Persistence 0.0009430 0.0002494 3.781 0.000825 ***

Age 0.0465734 0.0805621 0.578 0.568166

---

Signif. codes: 0 ‘***’ 0.001 ‘**’ 0.01 ‘*’ 0.05 ‘.’ 0.1 ‘ ’ 1

------------------------------------------------------------------

Sigma link function: logit

Sigma Coefficients:

Estimate Std. Error t value Pr(>|t|)

(Intercept) -1.3587 0.5402 -2.515 0.0184 *

------------------------------------------------------------------

No. of observations in the fit: 31

Degrees of Freedom for the fit: 5

Residual Deg. of Freedom: 26

at cycle: 4

Global Deviance: 109.0023

AIC: 119.0023

SBC: 126.1722

******************************************************************

##### Zero Altered Negative Binomial

###### summary(Diversity.Dogs.ZANBI)

******************************************************************

Family: c(“ZANBI”, “Zero Altered Negative Binomial type I”)

Call: gamlss(formula = Motor.Diversity ~ Object * Persistence + Age,

family = "ZANBI", data = na.omit(dogs), control = con, random = ~1 | Individual)

Fitting method: RS()

------------------------------------------------------------------

Mu link function: log

Mu Coefficients:

Estimate Std. Error t value Pr(>|t|)

(Intercept) 0.317742 0.596072 0.533 0.599

ObjectPipe 0.747607 0.534575 1.399 0.174

Persistence 0.003239 0.003607 0.898 0.377

Age 0.036956 0.126409 0.292 0.772

ObjectPipe:Persistence -0.002434 0.003660 -0.665 0.512

------------------------------------------------------------------

Sigma link function: log

Sigma Coefficients:

Estimate Std. Error t value Pr(>|t|)

(Intercept) -36.04 17960.53 -0.002 0.998

------------------------------------------------------------------

Nu link function: logit

Nu Coefficients:

Estimate Std. Error t value Pr(>|t|)

(Intercept) -1.0561 0.4105 -2.573 0.0153 *

---

Signif. codes: 0 ‘***’ 0.001 ‘**’ 0.01 ‘*’ 0.05 ‘.’ 0.1 ‘ ’ 1

------------------------------------------------------------------

No. of observations in the fit: 31

Degrees of Freedom for the fit: 7

Residual Deg. of Freedom: 24

at cycle: 3

Global Deviance: 105.2878

AIC: 119.2878

SBC: 129.3258

******************************************************************

###### dropterm(Diversity.Dogs.ZANBI, test = "Chisq")

Single term deletions for mu

Model: Motor.Diversity ~ Object * Persistence + Age

Df AIC LRT Pr(Chi)

<none> 119.29

Age 1 117.51 0.21815 0.64046

Object:Persistence 1 120.01 2.71950 0.09913 .

---

Signif. codes: 0 ‘***’ 0.001 ‘**’ 0.01 ‘*’ 0.05 ‘.’ 0.1 ‘ ’ 1

###### summary(Diversity.Dogs.ZANBI.2)

******************************************************************

Family: c(“ZANBI”, “Zero Altered Negative Binomial type I”)

Call: gamlss(formula = Motor.Diversity ~ Object + Persistence + Age,

family = "ZANBI", data = na.omit(dogs), control = con, random = ~1 | Individual)

Fitting method: RS()

------------------------------------------------------------------

Mu link function: log

Mu Coefficients:

Estimate Std. Error t value Pr(>|t|)

(Intercept) 0.4643207 0.5176760 0.897 0.378

ObjectPipe 0.5215666 0.4585333 1.137 0.265

Persistence 0.0008724 0.0005106 1.709 0.099 .

Age 0.0481085 0.1122336 0.429 0.672

---

Signif. codes: 0 ‘***’ 0.001 ‘**’ 0.01 ‘*’ 0.05 ‘.’ 0.1 ‘ ’ 1

------------------------------------------------------------------

Sigma link function: log

Sigma Coefficients:

Estimate Std. Error t value Pr(>|t|)

(Intercept) -36.04 17960.53 -0.002 0.998

------------------------------------------------------------------

Nu link function: logit

Nu Coefficients:

Estimate Std. Error t value Pr(>|t|)

(Intercept) -1.0561 0.4105 -2.573 0.0153 *

------------------------------------------------------------------

No. of observations in the fit: 31

Degrees of Freedom for the fit: 6

Residual Deg. of Freedom: 25

at cycle: 4

Global Deviance: 108.0073

AIC: 120.0073

SBC: 128.6113

******************************************************************

##### Zero Inflated Negative Binomial

###### summary(Diversity.Dogs.ZINBI)

******************************************************************

Family: c(“ZINBI”, “Zero Inflated Negative Binomial type I”)

Call: gamlss(formula = Motor.Diversity ~ Object * Persistence + Age,

family = "ZINBI", data = na.omit(dogs), control = con, random = ~1 | Individual)

Fitting method: RS()

------------------------------------------------------------------

Mu link function: log

Mu Coefficients:

Estimate Std. Error t value Pr(>|t|)

(Intercept) 0.451127 0.518061 0.871 0.392

ObjectPipe 0.514607 0.488324 1.054 0.302

Persistence 0.002886 0.003560 0.810 0.425

Age 0.041361 0.110586 0.374 0.711

ObjectPipe:Persistence -0.002010 0.003602 -0.558 0.582

------------------------------------------------------------------

Sigma link function: log

Sigma Coefficients:

Estimate Std. Error t value Pr(>|t|)

(Intercept) -36.06855 0.01724 -2092 <2e-16 ***

---

Signif. codes: 0 ‘***’ 0.001 ‘**’ 0.01 ‘*’ 0.05 ‘.’ 0.1 ‘ ’ 1

------------------------------------------------------------------

Nu link function: logit

Nu Coefficients:

Estimate Std. Error t value Pr(>|t|)

(Intercept) -1.3647 0.5133 -2.659 0.0125 *

------------------------------------------------------------------

No. of observations in the fit: 31

Degrees of Freedom for the fit: 7

Residual Deg. of Freedom: 24

at cycle: 4

Global Deviance: 106.9416

AIC: 120.9416

SBC: 130.9795

******************************************************************

###### dropterm(Diversity.Dogs.ZINBI, test = "Chisq")

Single term deletions for mu

Model: Motor.Diversity ~ Object * Persistence + Age

Df AIC LRT Pr(Chi)

<none> 120.94

Age 1 119.22 0.27629 0.5991

Object:Persistence 1 121.00 2.06025 0.1512

###### summary(Diversity.Dogs.ZINBI.2)

******************************************************************

Family: c(“ZINBI”, “Zero Inflated Negative Binomial type I”)

Call: gamlss(formula = Motor.Diversity ~ Object + Persistence + Age,

family = "ZINBI", data = na.omit(dogs), control = con, random = ~1 | Individual)

Fitting method: RS()

------------------------------------------------------------------

Mu link function: log

Mu Coefficients:

Estimate Std. Error t value Pr(>|t|)

(Intercept) 0.5623289 0.4602342 1.222 0.2323

ObjectPipe 0.3433202 0.3993139 0.860 0.3975

Persistence 0.0009447 0.0004307 2.193 0.0371 *

Age 0.0467424 0.0987681 0.473 0.6398

---

Signif. codes: 0 ‘***’ 0.001 ‘**’ 0.01 ‘*’ 0.05 ‘.’ 0.1 ‘ ’ 1

------------------------------------------------------------------

Sigma link function: log

Sigma Coefficients:

Estimate Std. Error t value Pr(>|t|)

(Intercept) -36.06932 0.01701 -2121 <2e-16 ***

------------------------------------------------------------------

Nu link function: logit

Nu Coefficients:

Estimate Std. Error t value Pr(>|t|)

(Intercept) -1.360 0.515 -2.64 0.013 *

------------------------------------------------------------------

No. of observations in the fit: 31

Degrees of Freedom for the fit: 6

Residual Deg. of Freedom: 25

at cycle: 5

Global Deviance: 109.0018

AIC: 121.0018

SBC: 129.6057

******************************************************************

##### Model AIC Values & Plots

|  | df | AIC |
| --- | --- | --- |
| **ZAP Models** |  |  |
| Diversity.Dogs.ZAP | 6 | 117.2878 |
| Diversity.Dogs.ZAP.2 | 5 | 118.007357 |
| **ZIP Models** |  |  |
| Diversity.Dogs.ZIP | 6 | 118.9416379 |
| Diversity.Dogs.ZIP.2 | 5 | 119.0022906 |
| **ZANBI Models** |  |  |
| Diversity.Dogs.ZANBI | 7 | 119.2878429 |
| Diversity.Dogs.ZANBI.2 | 6 | 120.007344 |
| **ZINBI Models** |  |  |
| Diversity.Dogs.ZINBI | 7 | 120.941578 |
| Diversity.Dogs.ZINBI.2 | 6 | 121.0018255 |

#### Plots


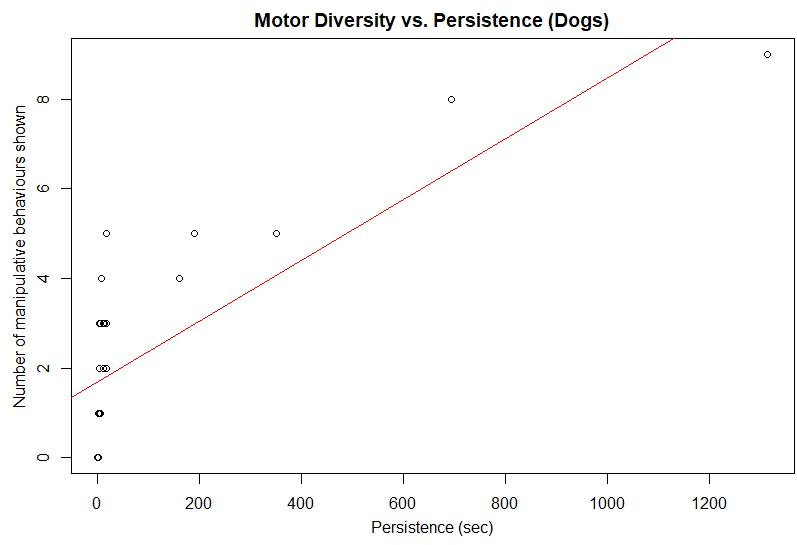


### Wolves

#### Response Variable Distribution

###### Wolves.Diversity.Distribution$fit

| Distribution | AIC |
| --- | --- |
| PO | 111.857751 |
| DPO | 113.200737 |
| NBII | 113.656754 |
| NBI | 113.656754 |
| GPO | 113.669782 |
| PIG | 113.69866 |
| ZAP | 113.70139 |
| ZIP2 | 113.857751 |
| ZIP | 113.857751 |
| ZANBI | 115.407244 |
| ZAPIG | 115.474817 |
| NBF | 115.656754 |
| SI | 115.656754 |
| DEL | 115.656754 |
| ZINBI | 115.656754 |
| SICHEL | 115.656754 |
| BNB | 115.681199 |
| ZIPIG | 115.69866 |

##### Density plots of distributions used in final models


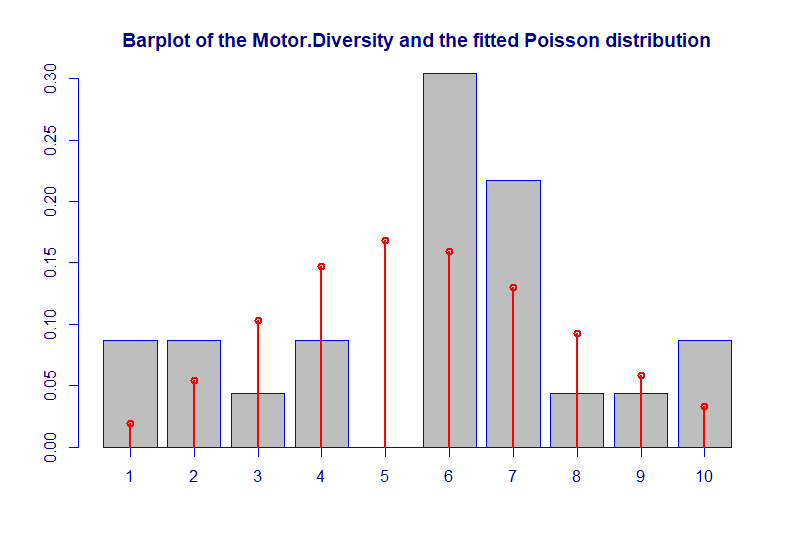

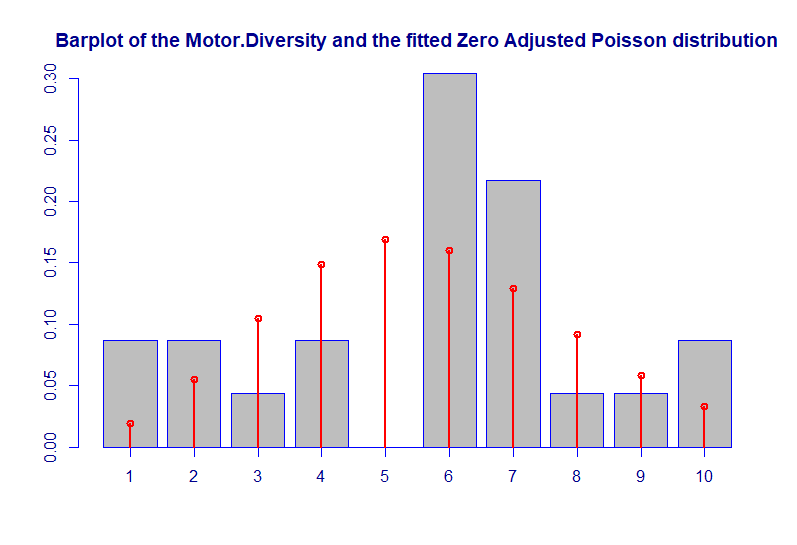


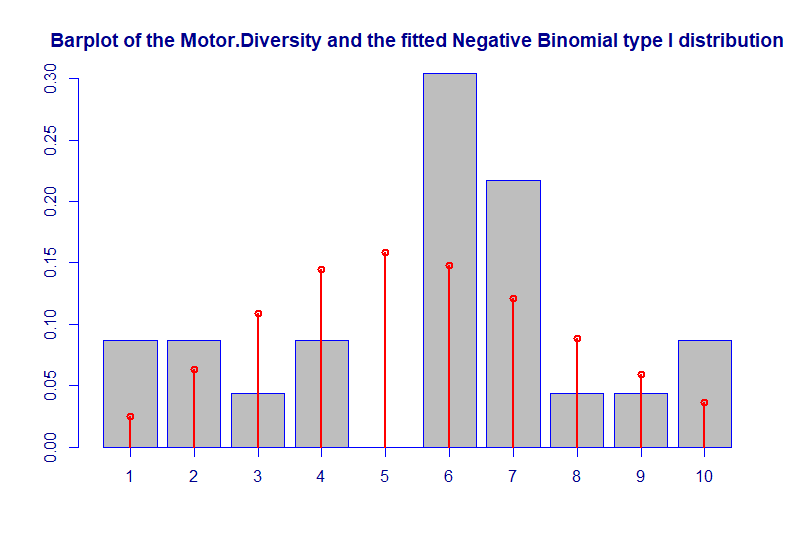

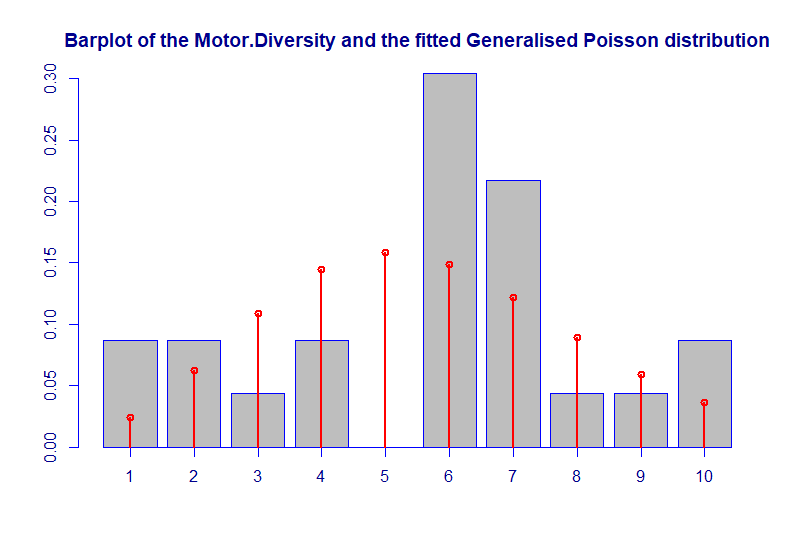


#### Model Distribution Selection

Diversity.Wolves.DISTRIBUTION <- gamlss(Motor.Diversity ~ Object*Persistence + Age,

random = ~1|Individual,

family = "DISTRIBUTION", data = wolves)

| Model | df | AIC |
| --- | --- | --- |
| Diversity.Wolves.DPO | 6 | 101.744393 |
| Diversity.Wolves.PO | 5 | 103.02143 |
| Diversity.Wolves.ZAP | 6 | 104.368964 |
| Diversity.Wolves.NBI | 6 | 105.02143 |
| Diversity.Wolves.GPO | 6 | 105.021491 |
| Diversity.Wolves.ZIP | 6 | 105.022016 |
| Diversity.Wolves.ZANBI | 7 | 106.369167 |
| Diversity.Wolves.DEL | 7 | 107.021477 |
| Diversity.Wolves.ZIPIG | 7 | 107.021944 |
| Diversity.Wolves.NBF | 7 | 107.022231 |
| Diversity.Wolves.ZINBI | 7 | 107.022231 |

##### Model Plots

###### plot(Diversity.Wolves.DPO) & wp(Diversity.Wolves.DPO)

###### plot(Diversity.Wolves.PO) & wp(Diversity.Wolves.PO)

###### plot(Diversity.Wolves.ZAP) & wp(Diversity.Wolves.ZAP)

###### plot(Diversity.Wolves.NBI) & wp(Diversity.Wolves.NBI)

###### plot(Diversity.Wolves.GPO) & wp(Diversity.Wolves.GPO)

###### plot(Diversity.Wolves.ZIP) & wp(Diversity.Wolves.ZIP)

###### plot(Diversity.Wolves.ZANBI) & wp(Diversity.Wolves.ZANBI)

###### plot(Diversity.Wolves.DEL) & wp(Diversity.Wolves.DEL)

###### plot(Diversity.Wolves.ZIPIG) & wp(Diversity.Wolves.ZIPIG)

###### plot(Diversity.Wolves.NBF) & wp(Diversity.Wolves.NBF)

###### plot(Diversity.Wolves.ZINBI) & wp(Diversity.Wolves.ZINBI)

#### Model Reduction and Validation

##### Poisson

###### summary(Diversity.Wolves.PO)

******************************************************************

Family: c(“PO”, “Poisson”)

Call: gamlss(formula = Motor.Diversity ~ Object * Persistence + Age,

family = "PO", data = na.omit(wolves), control = con, random = ~1 | Individual)

Fitting method: RS()

------------------------------------------------------------------

Mu link function: log

Mu Coefficients:

Estimate Std. Error t value Pr(>|t|)

(Intercept) 1.4140292 0.3902138 3.624 0.00194 **

ObjectPipe -0.5530735 0.2559170 -2.161 0.04441 *

Persistence 0.0003478 0.0003086 1.127 0.27455

Age 0.0545874 0.0512440 1.065 0.30085

ObjectPipe:Persistence 0.0006448 0.0003970 1.624 0.12173

---

Signif. codes: 0 ‘***’ 0.001 ‘**’ 0.01 ‘*’ 0.05 ‘.’ 0.1 ‘ ’ 1

------------------------------------------------------------------

No. of observations in the fit: 23

Degrees of Freedom for the fit: 5

Residual Deg. of Freedom: 18

at cycle: 2

Global Deviance: 93.02143

AIC: 103.0214

SBC: 108.6989

******************************************************************

###### dropterm(Diversity.Wolves.PO, test = "Chisq")

Single term deletions for mu

Model: Motor.Diversity ~ Object * Persistence + Age

Df AIC LRT Pr(Chi)

<none> 103.02

Age 1 102.17 1.1459 0.2844

Object:Persistence 1 102.94 1.9148 0.1664

###### summary(Diversity.Wolves.PO.2)

******************************************************************

Family: c(“PO”, “Poisson”)

Call: gamlss(formula = Motor.Diversity ~ Object + Persistence + Age,

family = "PO", data = na.omit(wolves), control = con, random = ~1 | Individual)

Fitting method: RS()

------------------------------------------------------------------

Mu link function: log

Mu Coefficients:

Estimate Std. Error t value Pr(>|t|)

(Intercept) 1.2142231 0.3675870 3.303 0.00374 **

ObjectPipe -0.2832031 0.1764174 -1.605 0.12492

Persistence 0.0007218 0.0001938 3.724 0.00144 **

Age 0.0610446 0.0507405 1.203 0.24373

---

Signif. codes: 0 ‘***’ 0.001 ‘**’ 0.01 ‘*’ 0.05 ‘.’ 0.1 ‘ ’ 1

------------------------------------------------------------------

No. of observations in the fit: 23

Degrees of Freedom for the fit: 4

Residual Deg. of Freedom: 19

at cycle: 2

Global Deviance: 94.93626

AIC: 102.9363

SBC: 107.4782

******************************************************************

##### Zero Adjusted Poisson

###### summary(Diversity.Wolves.ZAP)

******************************************************************

Family: c(“ZAP”, “Zero Adjusted Poisson”)

Call: gamlss(formula = Motor.Diversity ~ Object * Persistence + Age,

family = “ZAP”, data = na.omit(wolves), control = con, random = ~1 | Individual)

Fitting method: RS()

------------------------------------------------------------------

Mu link function: log

Mu Coefficients:

Estimate Std. Error t value Pr(>|t|)

(Intercept) 1.4060427 0.3955977 3.554 0.00244 **

ObjectPipe -0.5893984 0.2642837 -2.230 0.03950 *

Persistence 0.0003497 0.0003100 1.128 0.27492

Age 0.0558551 0.0520286 1.074 0.29803

ObjectPipe:Persistence 0.0006804 0.0004022 1.692 0.10896

---

Signif. codes: 0 ‘***’ 0.001 ‘**’ 0.01 ‘*’ 0.05 ‘.’ 0.1 ‘ ’ 1

------------------------------------------------------------------

Sigma link function: logit

Sigma Coefficients:

Estimate Std. Error t value Pr(>|t|)

(Intercept) -12.43 104.39 -0.119 0.907

------------------------------------------------------------------

No. of observations in the fit: 23

Degrees of Freedom for the fit: 6

Residual Deg. of Freedom: 17

at cycle: 2

Global Deviance: 92.36896

AIC: 104.369

SBC: 111.1819

******************************************************************

###### dropterm(Diversity.Wolves.ZAP, test = "Chisq")

Single term deletions for mu

Model: Motor.Diversity ~ Object * Persistence + Age

Df AIC LRT Pr(Chi)

<none> 104.37

Age 1 103.55 1.1825 0.2769

Object:Persistence 1 104.44 2.0692 0.1503

###### summary(Diversity.Wolves.ZAP.2)

******************************************************************

Family: c(“ZAP”, “Zero Adjusted Poisson”)

Call: gamlss(formula = Motor.Diversity ~ Object + Persistence + Age,

family = “ZAP”, data = na.omit(wolves), control = con, random = ~1 | Individual)

Fitting method: RS()

------------------------------------------------------------------

Mu link function: log

Mu Coefficients:

Estimate Std. Error t value Pr(>|t|)

(Intercept) 1.1914525 0.3752959 3.175 0.00525 **

ObjectPipe -0.3068588 0.1801701 -1.703 0.10574

Persistence 0.0007108 0.0001977 3.596 0.00207 **

Age 0.0651915 0.0516929 1.261 0.22336

---

Signif. codes: 0 ‘***’ 0.001 ‘**’ 0.01 ‘*’ 0.05 ‘.’ 0.1 ‘ ’ 1

------------------------------------------------------------------

Sigma link function: logit

Sigma Coefficients:

Estimate Std. Error t value Pr(>|t|)

(Intercept) -12.43 104.39 -0.119 0.907

------------------------------------------------------------------

No. of observations in the fit: 23

Degrees of Freedom for the fit: 5

Residual Deg. of Freedom: 18

at cycle: 2

Global Deviance: 94.43815

AIC: 104.4381

SBC: 110.1156

******************************************************************

##### Negative Binomial type I

###### summary(Diversity.Wolves.NBI)

******************************************************************

Family: c(“NBI”, “Negative Binomial type I”)

Call: gamlss(formula = Motor.Diversity ~ Object * Persistence + Age,

family = “NBI”, data = na.omit(wolves), control = con, random = ~1 | Individual)

Fitting method: RS()

------------------------------------------------------------------

Mu link function: log

Mu Coefficients:

Estimate Std. Error t value Pr(>|t|)

(Intercept) 1.4140292 0.3911258 3.615 0.00198 **

ObjectPipe -0.5530735 0.2629323 -2.103 0.04975 *

Persistence 0.0003478 0.0003595 0.968 0.34607

Age 0.0545874 0.0512004 1.066 0.30045

ObjectPipe:Persistence 0.0006448 0.0004669 1.381 0.18420

---

Signif. codes: 0 ‘***’ 0.001 ‘**’ 0.01 ‘*’ 0.05 ‘.’ 0.1 ‘ ’ 1

------------------------------------------------------------------

Sigma link function: log

Sigma Coefficients:

Estimate Std. Error t value Pr(>|t|)

(Intercept) -36.08189 0.01004 -3592 <2e-16 ***

------------------------------------------------------------------

No. of observations in the fit: 23

Degrees of Freedom for the fit: 6

Residual Deg. of Freedom: 17

at cycle: 3

Global Deviance: 93.02143

AIC: 105.0214

SBC: 111.8344

******************************************************************

###### dropterm(Diversity.Wolves.NBI, test = "Chisq")

Single term deletions for mu

Model: Motor.Diversity ~ Object * Persistence + Age

Df AIC LRT Pr(Chi)

<none> 105.02

Age 1 104.17 1.1459 0.2844

Object:Persistence 1 104.94 1.9148 0.1664

###### summary(Diversity.Wolves.NBI.2)

******************************************************************

Family: c(“NBI”, “Negative Binomial type I”)

Call: gamlss(formula = Motor.Diversity ~ Object + Persistence + Age,

family = “NBI”, data = na.omit(wolves), control = con, random = ~1 | Individual)

Fitting method: RS()

------------------------------------------------------------------

Mu link function: log

Mu Coefficients:

Estimate Std. Error t value Pr(>|t|)

(Intercept) 1.2142231 0.3683111 3.297 0.00379 **

ObjectPipe -0.2832031 0.1766578 -1.603 0.12540

Persistence 0.0007218 0.0002297 3.142 0.00537 **

Age 0.0610446 0.0507599 1.203 0.24390

---

Signif. codes: 0 ‘***’ 0.001 ‘**’ 0.01 ‘*’ 0.05 ‘.’ 0.1 ‘ ’ 1

------------------------------------------------------------------

Sigma link function: log

Sigma Coefficients:

Estimate Std. Error t value Pr(>|t|)

(Intercept) -36.08192 0.01004 -3595 <2e-16 ***

------------------------------------------------------------------

No. of observations in the fit: 23

Degrees of Freedom for the fit: 5

Residual Deg. of Freedom: 18

at cycle: 3

Global Deviance: 94.93626

AIC: 104.9363

SBC: 110.6137

******************************************************************

##### Generalised Poisson

###### summary(Diversity.Wolves.GPO)

******************************************************************

Family: c(“GPO”, “Generalised Poisson”)

Call: gamlss(formula = Motor.Diversity ~ Object * Persistence + Age,

family = “GPO”, data = na.omit(wolves), control = con, random = ~1 | Individual)

Fitting method: RS()

------------------------------------------------------------------

Mu link function: log

Mu Coefficients:

Estimate Std. Error t value Pr(>|t|)

(Intercept) 1.4101449 0.7615412 1.852 0.0805 .

ObjectPipe -0.5505080 0.4935132 -1.115 0.2793

Persistence 0.0003530 0.0009641 0.366 0.7185

Age 0.0546569 0.0911584 0.600 0.5563

ObjectPipe:Persistence 0.0006471 0.0010982 0.589 0.5630

---

Signif. codes: 0 ‘***’ 0.001 ‘**’ 0.01 ‘*’ 0.05 ‘.’ 0.1 ‘ ’ 1

------------------------------------------------------------------

Sigma link function: log

Sigma Coefficients:

Estimate Std. Error t value Pr(>|t|)

(Intercept) -25.83 20851.44 -0.001 0.999

------------------------------------------------------------------

No. of observations in the fit: 23

Degrees of Freedom for the fit: 6

Residual Deg. of Freedom: 17

at cycle: 3

Global Deviance: 93.02149

AIC: 105.0215

SBC: 111.8345

******************************************************************

###### dropterm(Diversity.Wolves.GPO, test = "Chisq")

Single term deletions for mu

Model: Motor.Diversity ~ Object * Persistence + Age

Df AIC LRT Pr(Chi)

<none> 105.02

Age 1 104.17 1.1468 0.2842

Object:Persistence 1 104.94 1.9169 0.1662

###### summary(Diversity.Wolves.GPO.2)

******************************************************************

Family: c(“GPO”, “Generalised Poisson”)

Call: gamlss(formula = Motor.Diversity ~ Object + Persistence + Age,

family = “GPO”, data = na.omit(wolves), control = con, random = ~1 | Individual)

Fitting method: RS()

------------------------------------------------------------------

Mu link function: log

Mu Coefficients:

Estimate Std. Error t value Pr(>|t|)

(Intercept) 1.2131412 0.6203147 1.956 0.0654 .

ObjectPipe -0.2791210 0.3151429 -0.886 0.3869

Persistence 0.0007308 0.0005000 1.462 0.1602

Age 0.0604934 0.0824273 0.734 0.4720

---

Signif. codes: 0 ‘***’ 0.001 ‘**’ 0.01 ‘*’ 0.05 ‘.’ 0.1 ‘ ’ 1

------------------------------------------------------------------

Sigma link function: log

Sigma Coefficients:

Estimate Std. Error t value Pr(>|t|)

(Intercept) -36.04 20851.44 -0.002 0.999

------------------------------------------------------------------

No. of observations in the fit: 23

Degrees of Freedom for the fit: 5

Residual Deg. of Freedom: 18

at cycle: 3

Global Deviance: 94.93842

AIC: 104.9384

SBC: 110.6159

******************************************************************

##### Model AIC Values & Plots

|  | df | AIC |
| --- | --- | --- |
| **PO Models** |  |  |
| Diversity.Wolves.PO.2 | 4 | 102.936263 |
| Diversity.Wolves.PO | 5 | 103.02143 |
| **ZAP Models** |  |  |
| Diversity.Wolves.ZAP | 6 | 104.3689642 |
| Diversity.Wolves.ZAP.2 | 5 | 104.4381486 |
| **NBI Models** |  |  |
| Diversity.Wolves.NBI.2 | 5 | 104.936263 |
| Diversity.Wolves.NBI | 6 | 105.02143 |
| **GPO Models** |  |  |
| Diversity.Wolves.GPO.2 | 5 | 104.938424 |
| Diversity.Wolves.GPO | 6 | 105.021491 |

#### Plots

## Individual Consistency Models

### Consistency in Persistence

#### Response Variable Distribution

###### Persistence.Consistency.Distribution$fit

| Distribution | AIC | Errors in Plot? |
| --- | --- | --- |
| LOGITNO | -90.4823 | No |
| GB1 | -88.2188 | No |
| BE | -81.7263 | No |
| BEo | -81.7263 | No |
| BEINF0 | -79.7263 | Yes |
| BEOI | -79.7263 | Yes |
| BEZI | -79.7263 | Yes |
| BEINF1 | -79.7263 | Yes |
| SIMPLEX | -77.8157 | No |
| BEINF | -77.7263 | Yes |

##### Density plots of distributions used in final models

#### Model Distribution Selection

Persistence.Consistency.DISTRIBUTION <- gamlss(persistence ~ Species,

family = "DISTRIBUTION", data = consistency)

| Model | df | AIC | Residuals Outside CI |
| --- | --- | --- | --- |
| Persistence.Consistency.LOGITNO | 3 | -95.2377192 | 1 |
| Persistence.Consistency.GB1 | 5 | -91.8139533 | 0 |
| Persistence.Consistency.BE | 3 | -83.2899177 | 4 |
| Persistence.Consistency.BEo | 3 | -83.0986407 | 3 |
| Persistence.Consistency.SIMPLEX | 3 | -78.5299752 | 8 |

##### Model Plots

###### plot(Persistence.Consistency.LOGITNO) & wp(Persistence.Consistency. LOGITNO)

###### plot(Persistence.Consistency.GB1) & wp(Persistence.Consistency.GB1)

###### plot(Persistence.Consistency.BE) & wp(Persistence.Consistency.BE)

###### plot(Persistence.Consistency.BEo) & wp(Persistence.Consistency.BEo)

###### plot(Persistence.Consistency.SIMPLEX) & wp(Persistence.Consistency.SIMPLEX)

#### Model Validation

##### Generalised Beta type 1

###### summary(Persistence.Consistency.GB1)

******************************************************************

Family: c("GB1", "Generalized beta type 1")

Call: gamlss(formula = persistence ~ Species, family = "GB1",

data = na.omit(consistency), control = con)

Fitting method: RS()

------------------------------------------------------------------

Mu link function: logit

Mu Coefficients:

Estimate Std. Error t value Pr(>|t|)

(Intercept) -0.8357 0.8077 -1.035 0.3120

SpeciesWolf -1.0719 0.4640 -2.310 0.0306 *

---

Signif. codes: 0 ‘***’ 0.001 ‘**’ 0.01 ‘*’ 0.05 ‘.’ 0.1 ‘ ’ 1

------------------------------------------------------------------

Sigma link function: logit

Sigma Coefficients:

Estimate Std. Error t value Pr(>|t|)

(Intercept) 0.4096 0.3414 1.2 0.243

------------------------------------------------------------------

Nu link function: log

Nu Coefficients:

Estimate Std. Error t value Pr(>|t|)

(Intercept) 4.715 1.121 4.205 0.000366 ***

------------------------------------------------------------------

Tau link function: log

Tau Coefficients:

Estimate Std. Error t value Pr(>|t|)

(Intercept) 1.4259 0.7247 1.968 0.0619 .

------------------------------------------------------------------

No. of observations in the fit: 27

Degrees of Freedom for the fit: 5

Residual Deg. of Freedom: 22

at cycle: 79

Global Deviance: -101.814

AIC: -91.81395

SBC: -85.33477

******************************************************************

##### Logit Normal

###### summary(Persistence.Consistency.LOGITNO)

******************************************************************

Family: c("LOGITNO", "Logit Normal")

Call: gamlss(formula = persistence ~ Species, family = "LOGITNO",

data = na.omit(consistency), control = con)

Fitting method: RS()

------------------------------------------------------------------

Mu link function: logit

Mu Coefficients:

Estimate Std. Error t value Pr(>|t|)

(Intercept) 4.8053 0.6005 8.002 3.14e-08 ***

SpeciesWolf -2.4954 0.9007 -2.770 0.0106 *

---

Signif. codes: 0 ‘***’ 0.001 ‘**’ 0.01 ‘*’ 0.05 ‘.’ 0.1 ‘ ’ 1

------------------------------------------------------------------

Sigma link function: log

Sigma Coefficients:

Estimate Std. Error t value Pr(>|t|)

(Intercept) 0.8440 0.1361 6.202 2.08e-06 ***

------------------------------------------------------------------

No. of observations in the fit: 27

Degrees of Freedom for the fit: 3

Residual Deg. of Freedom: 24

at cycle: 2

Global Deviance: -101.2377

AIC: -95.23772

SBC: -91.35021

******************************************************************

### Consistency in Contact Latency

#### Response Variable Distribution

###### Latency.Consistency.Distribution$fit

| Distribution | AIC | Errors in Plot? |
| --- | --- | --- |
| SIMPLEX | -35.8643 | No |
| LOGITNO | -34.118 | No |
| GB1 | -31.593 | Yes |
| BEo | -31.4833 | No |
| BE | -31.4833 | No |
| BEINF0 | -29.4833 | Yes |
| BEINF1 | -29.4833 | Yes |
| BEOI | -29.4833 | Yes |
| BEZI | -29.4833 | Yes |
| BEINF | -27.4833 | Yes |

##### Density plots of distributions used in final models

#### Model Distribution Selection

Latency.Consistency.DISTRIBUTION <- gamlss(latency ~ Species,

family = "DISTRIBUTION", data = consistency)

| Model | df | AIC |
| --- | --- | --- |
| Latency.Consistency.SIMPLEX | 3 | -39.7189678 |
| Latency.Consistency.LOGITNO | 3 | -39.6086058 |
| Latency.Consistency.BEo | 3 | -39.5609832 |
| Latency.Consistency.BE | 3 | -35.9167115 |

##### Model Plots

###### plot(Latency.Consistency.SIMPLEX) & wp(Latency.Consistency.SIMPLEX)

###### plot(Latency.Consistency.LOGITNO) & wp(Latency.Consistency. LOGITNO)

###### plot(Latency.Consistency.BEo) & wp(Latency.Consistency.BEo)

###### plot(Latency.Consistency.BE) & wp(Latency.Consistency.BE)

#### Model Validation

##### Simplex

###### summary(Latency.Consistency.SIMPLEX)

******************************************************************

Family: c("SIMPLEX", "Simplex")

Call: gamlss(formula = latency ~ Species, family = "SIMPLEX",

data = na.omit(consistency), control = con)

Fitting method: RS()

------------------------------------------------------------------

Mu link function: logit

Mu Coefficients:

Estimate Std. Error t value Pr(>|t|)

(Intercept) 2.0691 0.2399 8.624 1.16e-08 ***

SpeciesWolf -0.9757 0.3729 -2.616 0.0154 *

---

Signif. codes: 0 ‘***’ 0.001 ‘**’ 0.01 ‘*’ 0.05 ‘.’ 0.1 ‘ ’ 1

------------------------------------------------------------------

Sigma link function: log

Sigma Coefficients:

Estimate Std. Error t value Pr(>|t|)

(Intercept) 1.2116 0.1387 8.737 9.16e-09 ***

------------------------------------------------------------------

No. of observations in the fit: 26

Degrees of Freedom for the fit: 3

Residual Deg. of Freedom: 23

at cycle: 3

Global Deviance: -45.71897

AIC: -39.71897

SBC: -35.94468

******************************************************************

##### Logit Normal

###### summary(Persistence.Consistency.LOGITNO)

******************************************************************

Family: c("LOGITNO", "Logit Normal")

Call: gamlss(formula = latency ~ Species, family = "LOGITNO",

data = na.omit(consistency), control = con)

Fitting method: RS()

------------------------------------------------------------------

Mu link function: logit

Mu Coefficients:

Estimate Std. Error t value Pr(>|t|)

(Intercept) 2.3858 0.2698 8.844 7.36e-09 ***

SpeciesWolf -1.2220 0.4147 -2.946 0.00725 **

---

Signif. codes: 0 ‘***’ 0.001 ‘**’ 0.01 ‘*’ 0.05 ‘.’ 0.1 ‘ ’ 1

------------------------------------------------------------------

Sigma link function: log

Sigma Coefficients:

Estimate Std. Error t value Pr(>|t|)

(Intercept) 0.0438 0.1387 0.316 0.755

------------------------------------------------------------------

No. of observations in the fit: 26

Degrees of Freedom for the fit: 3

Residual Deg. of Freedom: 23

at cycle: 2

Global Deviance: -45.60861

AIC: -39.60861

SBC: -35.83432

******************************************************************

##### Beta Original

###### summary(Persistence.Consistency.BEo)

******************************************************************

Family: c("BEo", "Beta original")

Call: gamlss(formula = latency ~ Species, family = "BEo",

data = na.omit(consistency), control = con)

Fitting method: RS()

------------------------------------------------------------------

Mu link function: log

Mu Coefficients:

Estimate Std. Error t value Pr(>|t|)

(Intercept) 2.438 0.325 7.504 1.26e-07 ***

SpeciesWolf -1.173 0.343 -3.420 0.00234 **

---

Signif. codes: 0 ‘***’ 0.001 ‘**’ 0.01 ‘*’ 0.05 ‘.’ 0.1 ‘ ’ 1

------------------------------------------------------------------

Sigma link function: log

Sigma Coefficients:

Estimate Std. Error t value Pr(>|t|)

(Intercept) 0.3687 0.2520 1.463 0.157

------------------------------------------------------------------

No. of observations in the fit: 26

Degrees of Freedom for the fit: 3

Residual Deg. of Freedom: 23

at cycle: 10

Global Deviance: -45.56098

AIC: -39.56098

SBC: -35.78669

******************************************************************

##### Beta

###### summary(Persistence.Consistency.BE)

******************************************************************

Family: c("BE", "Beta")

Call: gamlss(formula = latency ~ Species, family = "BE",

data = na.omit(consistency), control = con)

Fitting method: RS()

------------------------------------------------------------------

Mu link function: logit

Mu Coefficients:

Estimate Std. Error t value Pr(>|t|)

(Intercept) 1.8643 0.2463 7.570 1.09e-07 ***

SpeciesWolf -0.8799 0.3241 -2.715 0.0124 *

---

Signif. codes: 0 ‘***’ 0.001 ‘**’ 0.01 ‘*’ 0.05 ‘.’ 0.1 ‘ ’ 1

------------------------------------------------------------------

Sigma link function: logit

Sigma Coefficients:

Estimate Std. Error t value Pr(>|t|)

(Intercept) -0.6192 0.1867 -3.316 0.00301 **

------------------------------------------------------------------

No. of observations in the fit: 26

Degrees of Freedom for the fit: 3

Residual Deg. of Freedom: 23

at cycle: 5

Global Deviance: -41.91671

AIC: -35.91671

SBC: -32.14242

******************************************************************
